# Supplementary material for: Primary and promiscuous functions coexist during evolutionary innovation through whole protein domain acquisitions
Source: eLife. 2020 Dec 15;9:e58061. doi: 10.7554/eLife.58061 (PMC7790495; doi:10.7554/eLife.58061)
Supplement: Supplementary file 1. [file elife-58061-supp1.docx]

Supplementary File 1: Mutations found in enrichment cycles

Mutations found in *intI1_8mut_*

| **Prevalent Clones** | **Mutations** | **Residue changes** |
| --- | --- | --- |
| 21/42 | G357A + T658A + C962G + A+49T | Y220N + A321G |
| 4/42 | T399A + G943A + C986T | H133Q + V315M + T329M |
| 4/42 | Wild type |  |
| 1/42 | A805G |  |
| 1/42 | C989T | T329M |
| 1/42 | T659A | Y220N |
| 1/42 | C837T |  |
| 1/42 | G288C + C516T |  |
| 1/42 | C177G + T947C | S59R + V315A |
| 1/42 | C9A + T423C + G864A + A1003T | T334S |
| 1/42 | G327A + G731T | R243L |
| 1/42 | G39A + A162C + G288T + T328C + T369A | S110P |
| 1/42 | T15A + T166C + G314C | G105A |
| 1/42 | G504A + G961A | D320N |
| 1/42 | G51T + G307A | E103K |
| 1/42 | C206T + T298G | A69V + W100G |

Mutations found in *alt1_8mut_*

| **Prevalent**  **Clones** | **Mutations** | **Residue changes** |
| --- | --- | --- |
| 6/31 | T944C | V315A |
| 3/31 | T519A + A972G | S173R |
| 5/31 | A305G + T519A + A651G | Q102R + S173R |
| 3/31 | G958A | D320N |
| 2/31 | C291T + G307A + G388A + T690C | E103K + E130K |
| 1/31 | C326A + T927A + T944C | P109H + V315A |
| 1/31 | T647G + T944C | L216W + V315A |
| 1/31 | G802T | A268S |
| 1/31 | T647G + T944C | L216W + V315A |
| 1/31 | T92C + G289C | L31P + D96H |
| 1/31 | C661T + T944C | P221S + V315A |
| 1/31 | C912T |  |
| 1/31 | G271A | G91R |
| 1/31 | T298A | W100R |
| 1/31 | G364A + T519G + G620C + G873C | V122I + S173R + R207T |
| 1/31 | C723G + G958A | D241E + D347N |
| 1/31 | G728A + T944C + C1009G | R243K + V315A + R337G |

Mutations found in *alt2_8mut_*

| **Prevalent clones** | **Mutations** | **Residue changes** |
| --- | --- | --- |
| 18/26 | A-4T + T944C | 5’ UTR + V315A |
| 2/26 | C-2T + A308C | 5’UTR + E103A |
| 1/26 | G307A | E103K |
| 5/26 | G958A | D320N |
