# Supplementary material for: Primary and promiscuous functions coexist during evolutionary innovation through whole protein domain acquisitions
Source: eLife. 2020 Dec 15;9:e58061. doi: 10.7554/eLife.58061 (PMC7790495; doi:10.7554/eLife.58061)
Supplement: Supplementary file 2. [file elife-58061-supp2.docx]

Supplementary File 2: bacterial strains used in this work.

**GENERAL STRAINS**

|  | | | |
| --- | --- | --- | --- |
| **Strain** | **Genotype** | **Use** | **Reference** |
| DH5α | *supE44 ΔlacU169* (Φ80*lacZ’ ΔM15) ΔargF hsdR17 recA1 endA1 gyrA96 thi-1 relA1* | General | Lab strain |
| UB5120 | F-pro met *recA56* gyrA [Nal^R^] | General | Martinez and de la Cruz. 1990 |
| β2163 | MG1655:: *Δ*dapA::(erm-pir)RP4-2-T c::Mu [Km^R^] | Donor strain in conjugation | Demarre *et al*. 2005 |
| B36 | MG1655 Δ*dapA* recA269::Tn10 attB::*attI1_WT_*-*attI1_STOP_*-*dapA* [Sp^R^] | Enrichment cycles and recombination tests | Escudero *et al*. 2016 |

**CONJUGATION ASSAYS**

| **Donor strains** | | | | | |
| --- | --- | --- | --- | --- | --- |
| **Strain** | **Genotype** | | | | **Reference** |
| 4137 | β2163/pSW23T::*attC_aadA7_* | | | | Bouvier *et al.* 2005 |
| 2714 | β2163/pSW23T::*attI1* | | | | Demarre *et al*. 2005 |
| H203 | β2163/p6944 (pSW23T:: *attC_aadA7-_*VCR_2/1_) | | | | Strain from this work.  Plasmid p6944 from Bouvier *et al*. 2009 |
| **Receptor strains** | | | | | |
| **Strain** | **Genotype** | **Parental allele** | | **Additional mutations** | **Resident plasmids** |
| 9669 | DH5α |  | | Wild type *intI1* | P929 (pSU38Δ::*attI1*) |
| H294 | “ | *intI1* | | T118S | “ |
| H291 | “ | “ | | D161G | “ |
| H297 | “ | “ | | G320D | “ |
| H306 | “ | “ | | T118S, D161G | “ |
| H303 | “ | “ | | T118S, G320D | “ |
| H300 | “ | “ | | D161G, G320D | “ |
| H309 | “ | “ | | T118S, D161G, G320D | “ |
| B903 | “ | “ | | T118S, D161G, H162Q, K219R, G320D | “ |
| B904 | “ | “ | | T118S, D161G, H162Q, K219R, D299E, G320D, | “ |
| B905 | “ | “ | | T118S, D161G, H162Q, K219R, D299E, G319E, G320D, A329T | “ |
| A283 | “ |  | | Wild type *alt1_l.e._* | “ |
| B506 | “ | *alt1_l.e._* | | D161, G319E | “ |
| B507 | “ | “ | | R12G, 3xSynon (Glu62+Leu113+Pro181) | “ |
| B654 | “ | “ | | K219R | “ |
| B655 | “ | “ | | H162Q | “ |
| B656 | “ | “ | | T96I, G320R, R337C | “ |
| B657 | “ | “ | | V318G | “ |
| B658 | “ | “ | | H48Q, A85V, D97G, M145V, G269E, L316 | “ |
| B965 | “ | “ | | T118S, D161G, G320D, H162Q, K219R, D299E, G319E, A329T | “ |
| A286 | “ |  | | Wild type *alt2_l.e._* | “ |
| B504 | “ | *alt2_l.e._* | | D299E, A329T, R324G, Synon (G417A) | “ |
| B505 | “ | “ | | D299E, A329T | “ |
| B659 | “ | “ | | D299E, A329T, V323A | “ |
| **Recipient strains for *attC* x *attC* excision assays with p6944** | | | | | |
| 3938 | “ | |  | Wild type *intI1* | None |
| H315 | “ | | *intI1* | T118S | None |
| H312 | “ | | “ | D161G | None |
| H318 | “ | | “ | G320D | None |
| 7617 | “ | | “ | T118S, D161G | None |
| H324 | “ | | “ | T118S, G320D | None |
| H321 | “ | | “ | D161G, G320D | None |
| 7618 | “ | | “ | T118S, D161G, G320D | None |
| B877 | “ | | “ | T118S, D161G, H162Q, K219R, G320D | None |
| B901 | “ | | “ | T118S, D161G, H162Q, K219R, D299E, G320D, | None |
| B902 | “ | | “ | T118S, D161G, H162Q, K219R, D299E, G319E, G320D, A329T | None |

**CHROMOSOMAL ASSAYS**

| **Strain** | **Genotype** | **Parental allele** | **Additional mutations** | **Observations** |
| --- | --- | --- | --- | --- |
| C006 | B36 | *intI1* | Wild type allele |  |
| C007 | “ | *alt1_l.e._* | Wild type allele |  |
| C008 | “ | *intI1* | *intI1_8mut_*: contains the same 8 mutations as B905 | Not referred to by strain number but rather as *XXX_8mut_* |
| C009 | “ | *alt1_l.e._* | *alt1_8mut_*: contains the same 8 mutations as B905 |  |
| C010 | “ | *alt2_l.e._* | *alt2_8mut_*: contains the same 8 mutations as B905 |  |
| C325 | “ | *intI1_8mut_* | *intI1_8mut_* + H133Q, V315M,T329M |  |
| C326 | “ | “ | *intI1_8mut_* + Y220N, A321G, 1Syn (G357A), A+49T (3’UTR) |  |
| C492 | “ | “ | *intI1_8mut_* + H133Q |  |
| C493 | “ | “ | *intI1_8mut_* + V315M |  |
| C494 | “ | “ | *intI1_8mut_* + T329M |  |
| C495 | “ | “ | *intI1_8mut_* + Syn (G357A) |  |
| C496 | “ | “ | *intI1_8mut_* + Y220N |  |
| C497 | “ | “ | *intI1_8mut_* + A321G |  |
| C498 | “ | “ | *intI1_8mut_* + A+49 (3’UTR) |  |
| C499 | “ | “ | *intI1_8mut_* + Y220N, Syn (G357A) |  |
| C500 | “ | “ | *intI1_8mut_* + Y220N, A+49 (3’UTR) |  |
| C118 | “ | *alt1_8mut_* | *alt1_8mut_* + V315A |  |
| C119 | “ | “ | *alt1_8mut_* + L216W, V315A |  |
| C120 | “ | “ | *alt1_8mut_* + P221S, V315A |  |
| C121 | “ | “ | *alt1_8mut_* + D241E, D320N |  |
| C122 | “ | “ | *alt1_8mut_* + E103K, E130K, 2 Syn |  |
| C123 | “ | “ | *alt1_8mut_* + S173R |  |
| C124 | “ | “ | *alt1_8mut_* + D320N |  |
| C125 | “ | “ | *alt1_8mut_* + Q102R + S173R |  |
| D472 | “ | “ | *alt1_8mut_* + E103K, 1 Syn |  |
| D473 | “ | “ | *alt1_8mut_* + E130K, 1 Syn |  |
| C126 | “ | *alt2_8mut_* | *alt2_8mut_* + V315A, A-4T (5’UTR) |  |
| C127 | “ | “ | *alt2_8mut_* + E103A, C-2T (5’UTR) |  |
| C128 | “ | “ | *alt2_8mut_* + D320N |  |

**EPISTASIS PURIFICATION**

| **Sequence** | **Strain** | **Genotype** | **Reaction** | **Additional mutations on the basis of the *intI1* allele** |
| --- | --- | --- | --- | --- |
| *intI1_8mut_* | C008 | B36 | *attI* x *attI* | T118S, D161G, H162Q, K219R, D299E, G319E, G320N, A329T |
|  | E450 | B413 | *attC* x *attC* |  |
|  | E458 | B415 | *attI* x *attC* |  |
|  | E526 | B37 | *attI* x *attI*  *inverted orientation (double clivage)* |  |
| Seq 3 | E393 | B36 | *attI* x *attI* | E103K, T118S, H162Q, S173R, K219R, Y220N, G319D, G320S, |
|  | E451 | B413 | *attC* x *attC* |  |
|  | E459 | B415 | *attI* x *attC* |  |
|  | E527 | B37 | *attI* x *attI*  *inverted orientation (double cleavage)* |  |
| Seq 5 | E394 | B36 | *attI* x *attI* | E103K, T118S, H162Q, S173R, K219R, Y220N, G319D, G320N, |
|  | E452 | B413 | *attC* x *attC* |  |
|  | E460 | B415 | *attI* x *attC* |  |
|  | E528 | B37 | *attI* x *attI*  *inverted orientation (double cleavage)* |  |
| Seq 7 | E395 | B36 | *attI* x *attI* | E103K, T118S, H162Q, S173R, K219R, G319E, G320N, A329T |
|  | E453 | B413 | *attC* x *attC* |  |
|  | E461 | B415 | *attI* x *attC* |  |
|  | E529 | B37 | *attI* x *attI*  *inverted orientation (double cleavage)* |  |
| Seq 19 | E396 | B36 | *attI* x *attI* | E103K, T118S, H162Q, S173R, K219R, Y220N, G319D, G320N, A329T |
|  | E530 | B37 | *attI* x *attI*  *inverted orientation (double cleavage)* |  |
| Seq 23 | E397 | “ | *attI* x *attI* | E103K, T118S, D161G, H162Q, S173R, K219R, Y220N, G319D, G320S |
|  | E531 | B37 | *attI* x *attI*  *inverted orientation (double cleavage)* |  |
| Seq 31 | E398 | “ | *attI* x *attI* | E103K, T118S, H162Q, S173R, M179I, K219R, Y220N, V315A, G319D, G320N, A329T |
|  | E532 | B37 | *attI* x *attI*  *inverted orientation (double cleavage)* |  |
