# Supplementary material for: Primary and promiscuous functions coexist during evolutionary innovation through whole protein domain acquisitions
Source: eLife. 2020 Dec 15;9:e58061. doi: 10.7554/eLife.58061 (PMC7790495; doi:10.7554/eLife.58061)
Supplement: Supplementary file 3. [file elife-58061-supp3.docx]

Supplementary File 3: plasmids and oligonuclotides used in this work.

**PLASMIDS**

| **Number** | **Genotype** | **Use** | **Reference** |
| --- | --- | --- | --- |
|  | pBAD18 | Expression vector for all integrase encoding alleles |  |
| p6944 | pSW23T::[P*_tac_*]-*attC_aadA7_lacI^q^-*VCR_2_*-pir116**(BOT) | Suicide vector carrying two *attC* sites, allowing to establish the *attC* x *attC* recombination frequency. | Bouvier *et al*. 2009 |
| p2714 | pSW23T::*attI1* | Suicide vector carrying an *attI1* site | Demarre *et al*. 2005 |
| p929 | pSU38Δ::*attI1* |  | Biskri *et al*. 2005 |

**OLIGOS**

| **Number** | **Name** | **Use** | **Sequence** |
| --- | --- | --- | --- |
| 1440 | pBAD F | Amplification of integrase encoding alleles from the pBAD backbone | AGATTAGCGGATCCTACCTG |
| 1441 | pBAD R |  | TTAATCTGTATCAGGCTGAA |
| **8mut in *intI1*** | | | |
| 366 | D161GsdmFOR | Insertion of D161G | AGGATCTGGATTTCGGTCACGGCACGATCATCGTGCGGGA |
| 367 | D161GsdmBACK |  | ATGATCGTGCCGTGACCGAAATCCAGATCCTTGACCCGCA |
| 3112 | His 162 Gln F | Insertion of H162Q | GGATCTGGATTTCGGTCAAGGCACGATCATCG |
| 3113 | His 162 Gln R |  | CGTGCCTTGACCGAAATCCAGATCCTTGACC |
| 3114 | Lys 219 Arg F | Insertion of K219R | CCTTGAGCGGAGGTATCCGCGCGCCGGGC |
| 3115 | Lys 219 Arg R |  | GCGCGGATACCTCCGCTCAAGGGCGTCGGG |
| 3116 | Asp 299 Glu F | Insertion of D299E | CCGTGCAGGAACTGCTCGGCCATTCCGACG |
| 3117 | Asp 299 Glu R |  | GGCCGAGCAGTTCCTGCACGGTTCGAATGTCG |
| 3118 | 319+329 F | Insertion of G319E, G320D, A329T | GTTGAAGATGCCGGAGTGCGCTCACCGCTTGATACGCTGCCGCCCC |
| 3119 | 319 R |  | GCGCACTCCGGCATCTTCAACTTTCAGCACATG |
| **8mut in *alt1_l.e._*** | | | |
| 3120 | alt1 118 F | Insertion of T118S | TCCCTGTCGTTCTTTCGCCCGACGAGGTTGTGAGA |
| 3121 | alt1 118 R |  | AACCTCGTCGGGCGAAAGAACGACAGGGAGTCTTC |
| 3122 | alt1 161 y 162 F | Insertion of D161G, H162Q | AAGACCTCGACTTTGGCCAAGGGACTATAATAGTCAGAGA |
| 3123 | alt1 161 y 162 R |  | CTATTATAGTCCCTTGGCCAAAGTCGAGGTCTTTCACTC |
| 3124 | alt1 299 F | Insertion of D299E | AGAACGGTTCAAGAACTTTTGGGGCACAGCGATGTG |
| 3125 | alt1 299 R |  | CTGTGCCCCAAAAGTTCTTGAACCGTTCTTATATC |
| 3126 | alt1 319 et 320 R | Insertion of G319E, G320D, A329T | GTCCAAAGGGCTTCTAACGCCTGCGTCCTCCACCTTAAGAACGTGAGTA |
| 3127 | alt1 329 F |  | GCAGGCGTTAGAAGCCCTTTGGACACTTTACCTCCGTTGACGTCTG |
| **8mut in *alt2_l.e._*** | | | |
| 3128 | alt2 118 F | Insertion of T118S | GCTTCCTGTTGTATTATCACCAGACGAGGTTGTGCG |
| 3129 | alt2 118 R |  | CAACCTCGTCTGGTGATAATACAACAGGAAGCCGACG |
| 3130 | alt2 161 y 162 F | Insertion of D161G, H162Q | AAGACTTGGACTTTGGACAGGGTACCATAATAGTACGCG |
| 3131 | alt2 161 y 162 R |  | TACTATTATGGTACCCTGTCCAAAGTCCAAGTCTTTCACG |
| 3132 | alt2 219 F | Insertion of K219R | GCACTGGAAAGAAGGTACCCTCGGGCAGGTCACTCG |
| 3133 | alt2 219 R |  | CCTGCCCGAGGGTACCTTCTTTCCAGTGCATCTGGC |
| 3134 | alt2 299 F | Insertion of D299E | ACGTACAGTCCAAGAATTGCTGGGACACTCG |
| 3135 | alt2 299 R |  | CGAGTGTCCCAGCAATTCTTGGACTGTACGTATATC |
| 3136 | alt2 319 et 320 R | Insertion of G319E, G320D, A329T | GTGTCCAATGGACTCCGGACGCCTGCATCTTCCACCTTTAAAACGTGAGTATATA |
| 3137 | alt2 329 F |  | ATGCAGGCGTCCGGAGTCCATTGGACACATTGCCTCCATTGACGTCGG |
| **E103K/E130K** | | | |
| 3509 | alt18xMut 319-338 | Separating E103K from E130K | CCAAGACCCAGCAGAAGACT |
| 3510 | alt18xMut 338-319 |  | AGTCTTCTGCTGGGTCTTGG |
| **Epistasis Library** | | | |
| 4366 | 320-374 |  | CTCGGCCGTCGCGGCGCTTGCCGGTGGTGCTGWCCCCGGATGAAGTGGTTCGCAT |
| 4367 | 341-287 |  | GGCAAGCGCCGCGACGGCCGAGGTCTTCCGATCTYCTGAAGCCAGGGCAGATCCG |
| 4368 | 470-504 |  | ATCTGGATTTCGRTCAMGGCACGATCATCGTGCGG |
| 4369 | 470-544 |  | ATCTGGATTTCGRTCAMGGCACGATCATCGTGCGGGAGGGCAAGGGCAGWAAGGATCGGGCCTTGATGTTACCCG |
| 4370 | 494-459 |  | ATCGTGCCKTGAYCGAAATCCAGATCCTTGACCCGC |
| 4371 | 647-676 |  | TTGAGCGGARGWATCCGCGCGCCGGGCATT |
| 4372 | 655-634 |  | CGCGGATWCYTCCGCTCAAGGGCGTCGGGAAG |
| 4373 | 908-952 |  | ATTCCGACGTCTCTACGACGATGATTTACACGCATGYGCTGAAAG |
| 4374 | 924-871 |  | CGTAGAGACGTCGGAATGGCCGAGCAGWTCCTGCACGGTTCGAATGTCGTAACC |
| 4375 | 936-908 |  | GTAAATCATCGTCGTAGAGACGTCGGAAT |
| 4376 | 953-997 |  | TTGRMRRTGSCGGAGTGCGCTCACCGCTTGATRCGCTGCCGCCCC |
| 4377 | 967-937 |  | CTCCGSCAYYKYCAACTTTCAGCRCATGCGT |
| 4378 | 978-1006 |  | GCTTGATRCGCTGCCGCCCCTCACTAGTG |
| 4379 | 997-968 |  | GGGGCGGCAGCGYATCAAGCGGTGAGCGCA |
