# Supplementary material for: Primary and promiscuous functions coexist during evolutionary innovation through whole protein domain acquisitions
Source: eLife. 2020 Dec 15;9:e58061. doi: 10.7554/eLife.58061 (PMC7790495; doi:10.7554/eLife.58061)
Supplement: Supplementary file 4. [file elife-58061-supp4.pdf]

Single Mutants

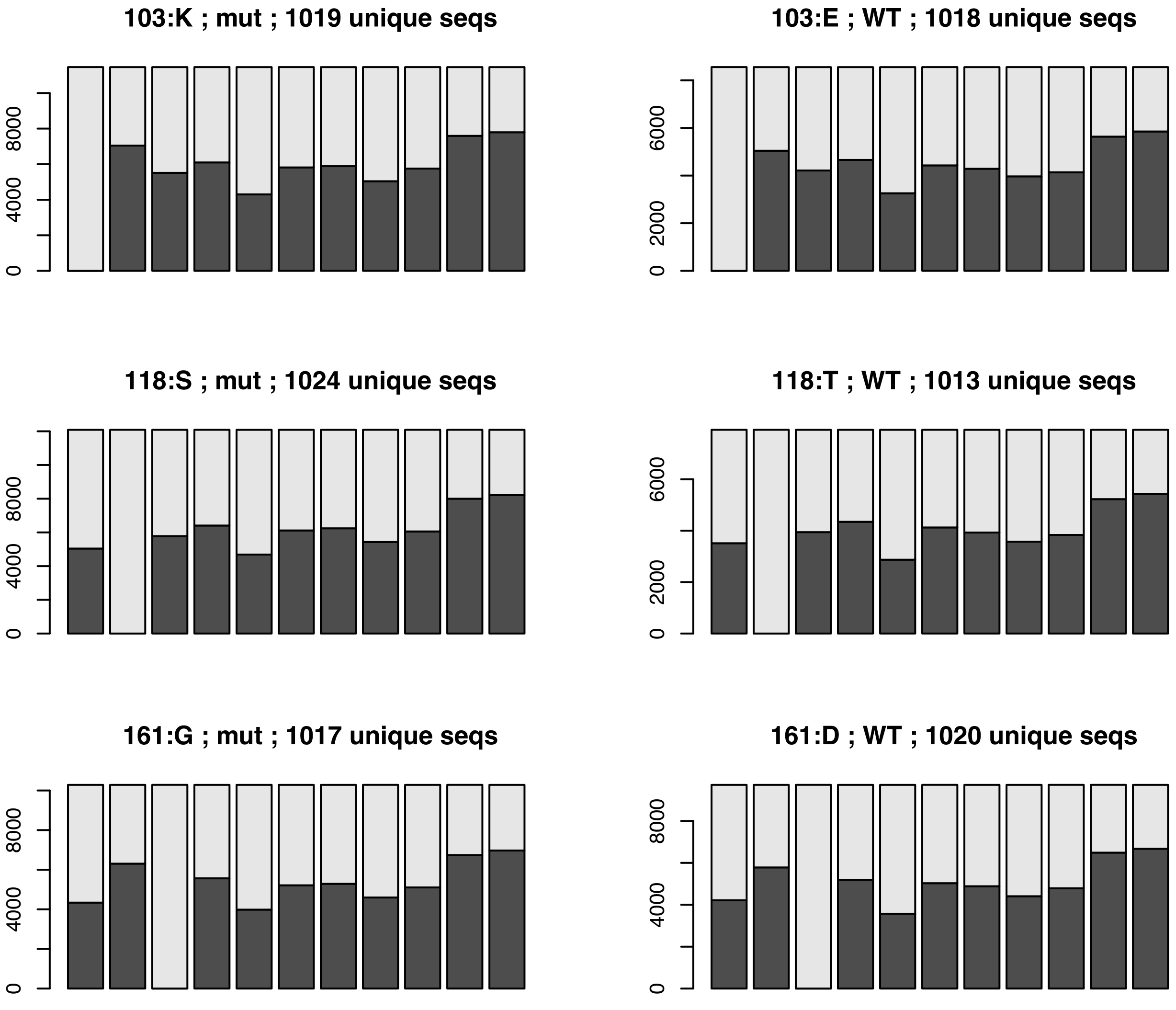

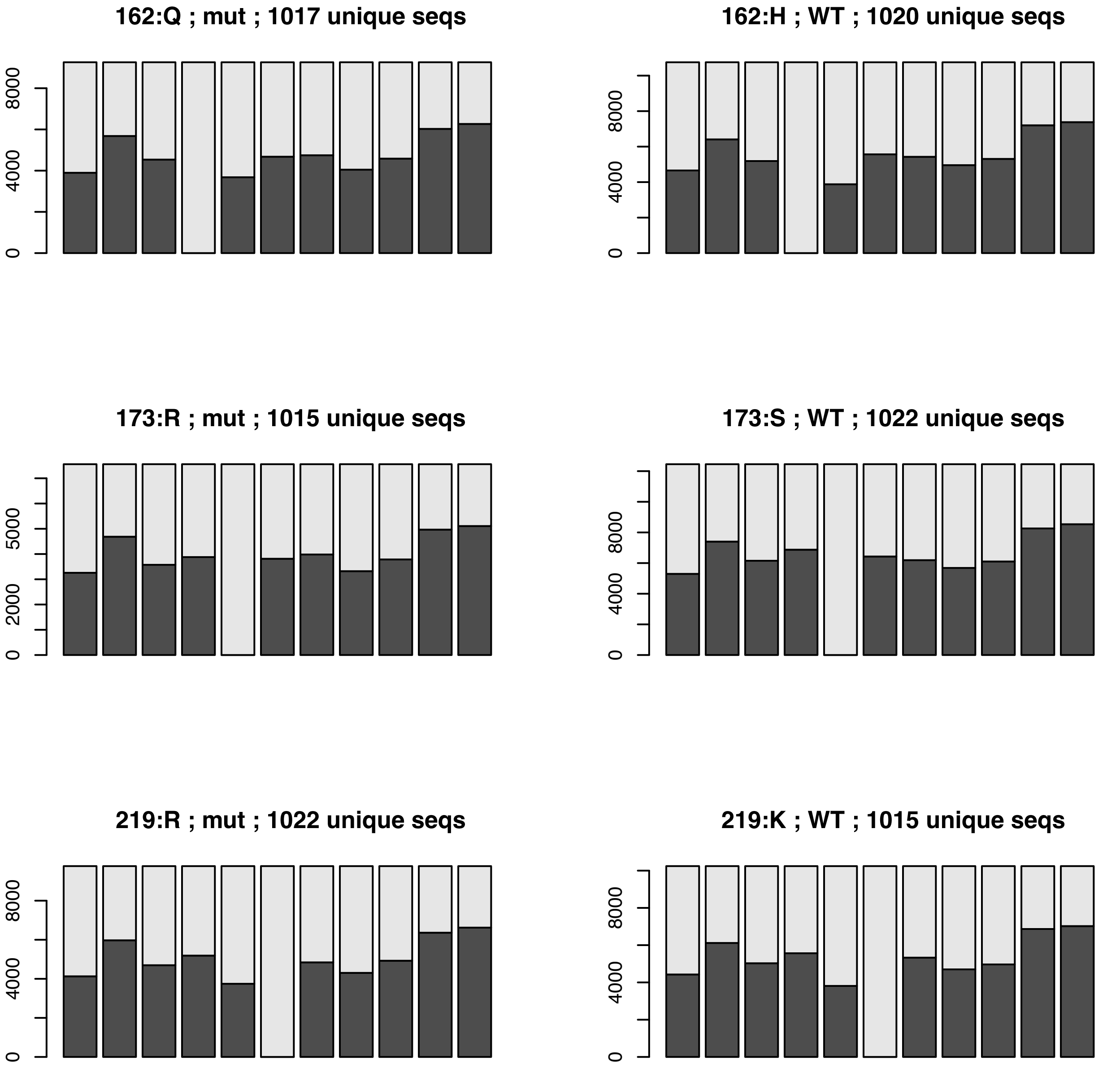

162:Q ; mut ; 1017 unique seqs
0
4000
8000
162:H ; WT ; 1020 unique seqs
0
4000
8000
173:R ; mut ; 1015 unique seqs
0
2000
5000
173:S ; WT ; 1022 unique seqs
0
4000
8000
219:R ; mut ; 1022 unique seqs
0
4000
8000
219:K ; WT ; 1015 unique seqs
0
4000
8000

220:N ; mut ; 1019 unique seqs

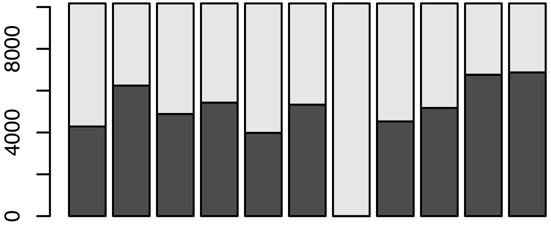

220:Y ; WT ; 1018 unique seqs

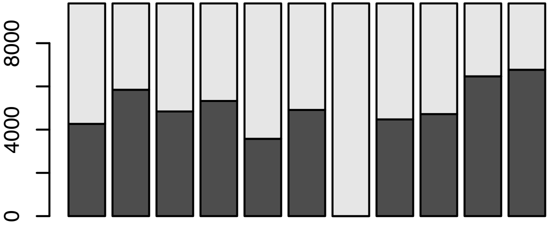

299:E ; mut ; 1018 unique seqs

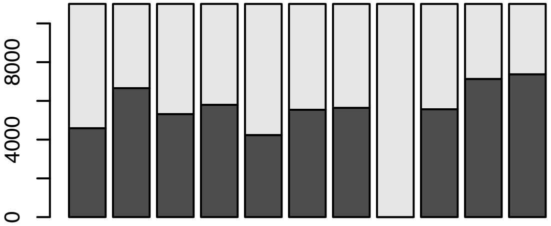

299:D ; WT ; 1019 unique seqs

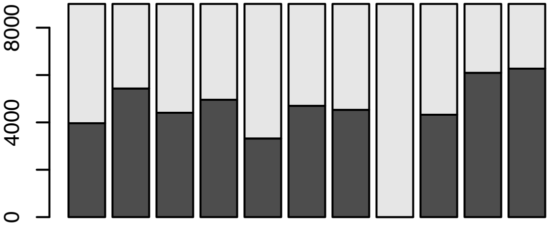

315:A ; mut ; 1019 unique seqs

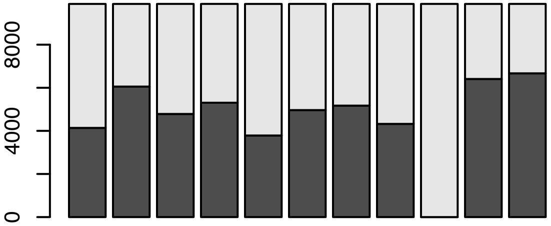

315:V ; WT ; 1018 unique seqs

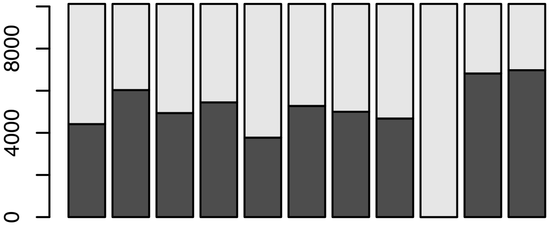

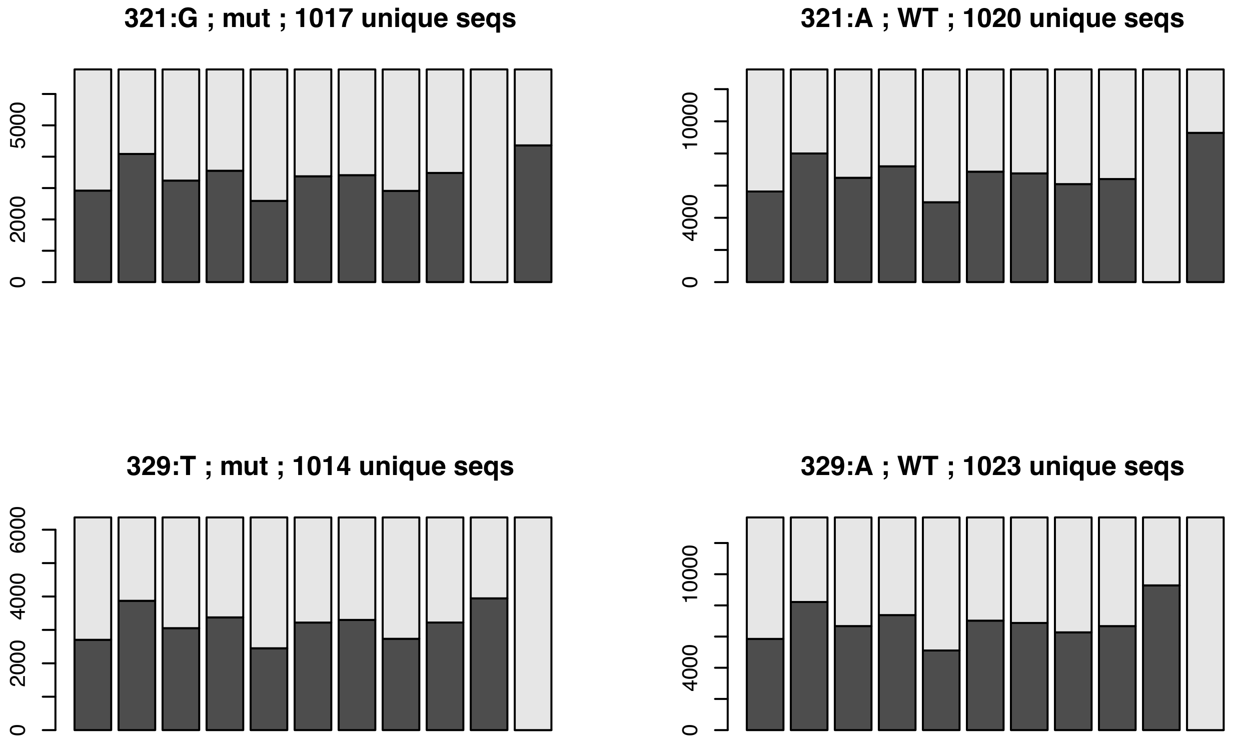
321:G ; mut ; 1017 unique seqs
0
2000
5000
321:A ; WT ; 1020 unique seqs
0
4000
10000
329:T ; mut ; 1014 unique seqs
0
2000
4000
6000
329:A ; WT ; 1023 unique seqs
0
4000
10000

Double Mutants

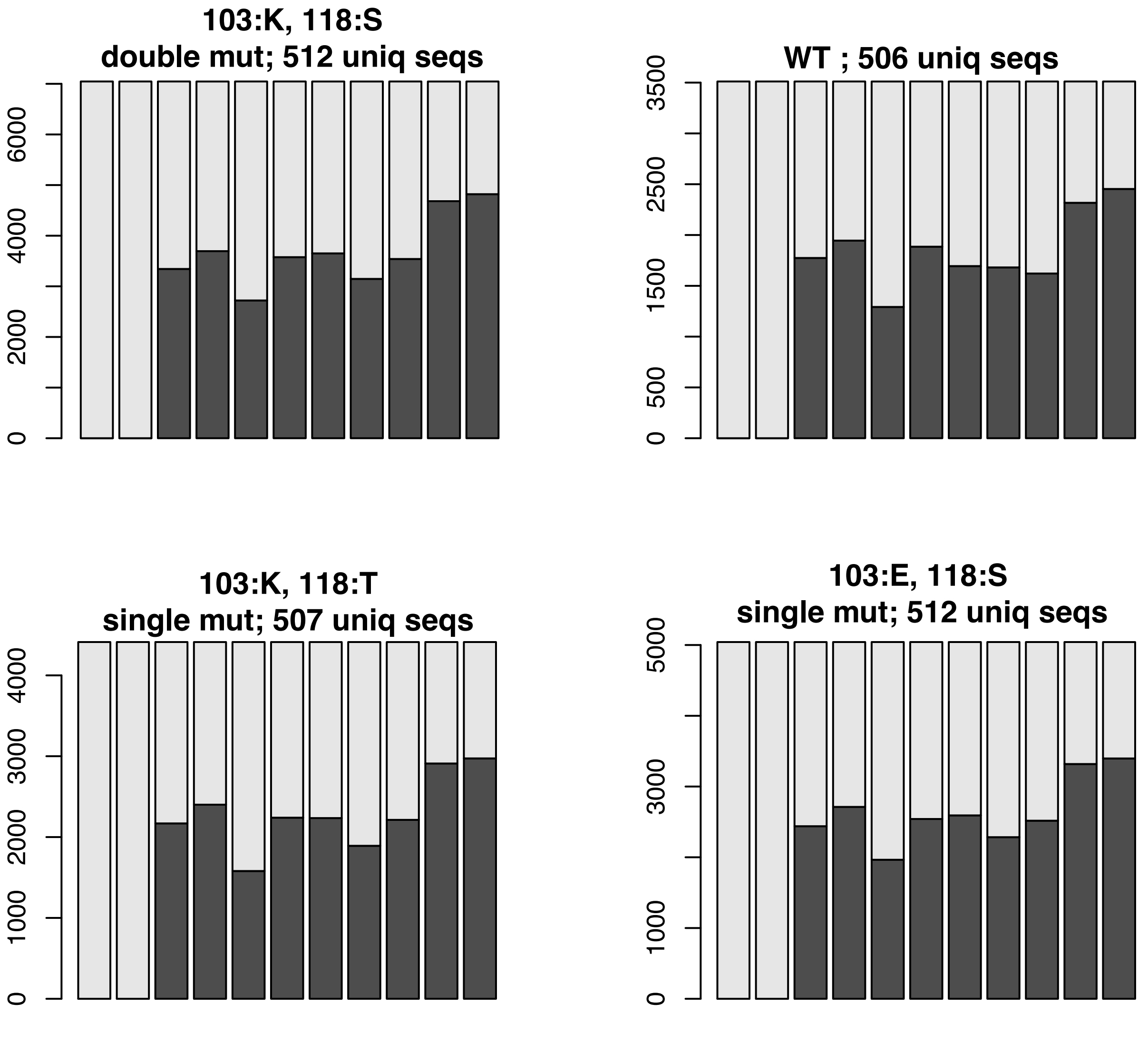

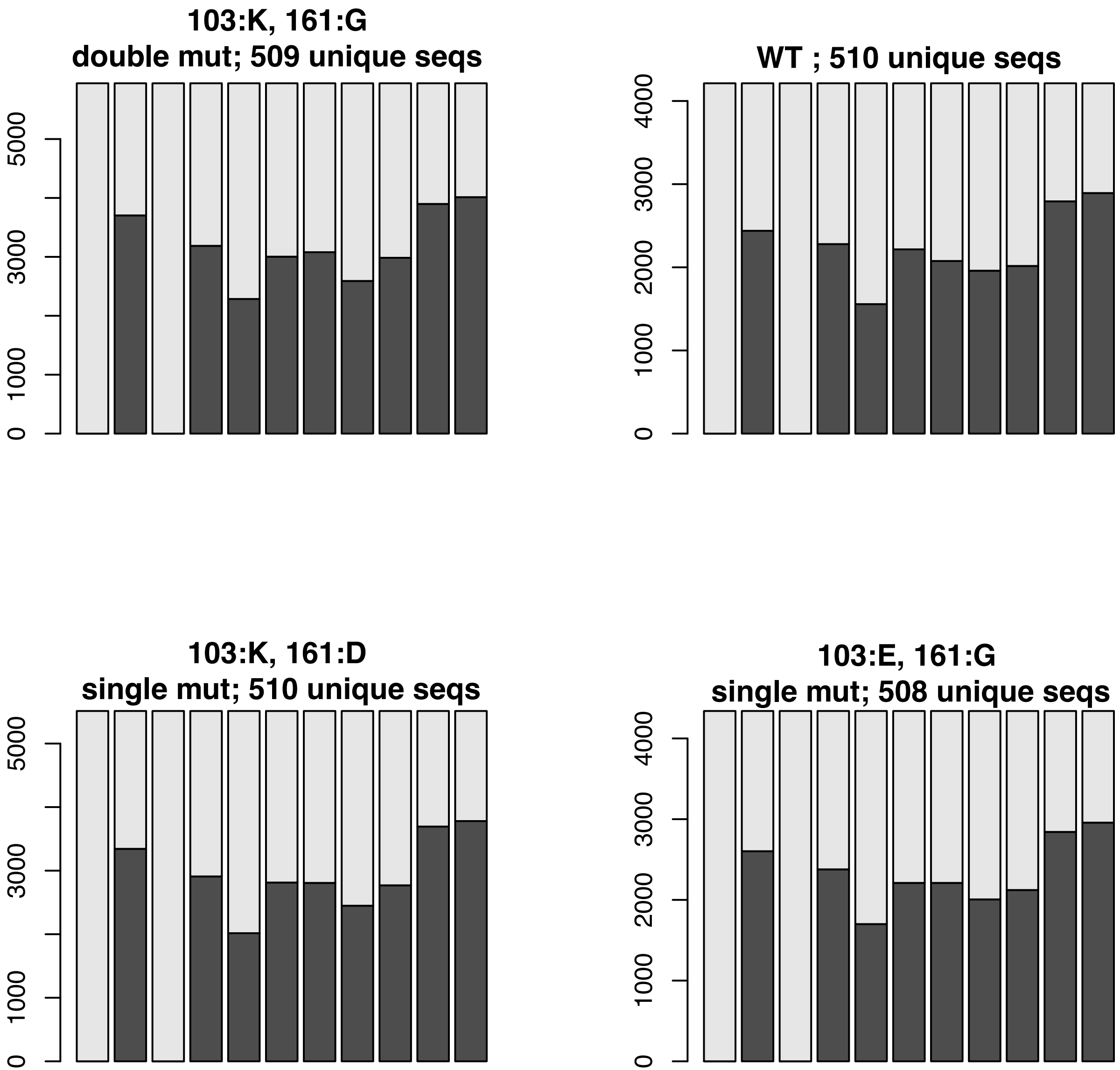
103:K, 161:G
double mut; 509 unique seqs
0
1000
3000
5000
WT ; 510 unique seqs
0
1000
2000
3000
4000
103:K, 161:D
single mut; 510 unique seqs
0
1000
3000
5000
103:E, 161:G
single mut; 508 unique seqs
0
1000
2000
3000
4000

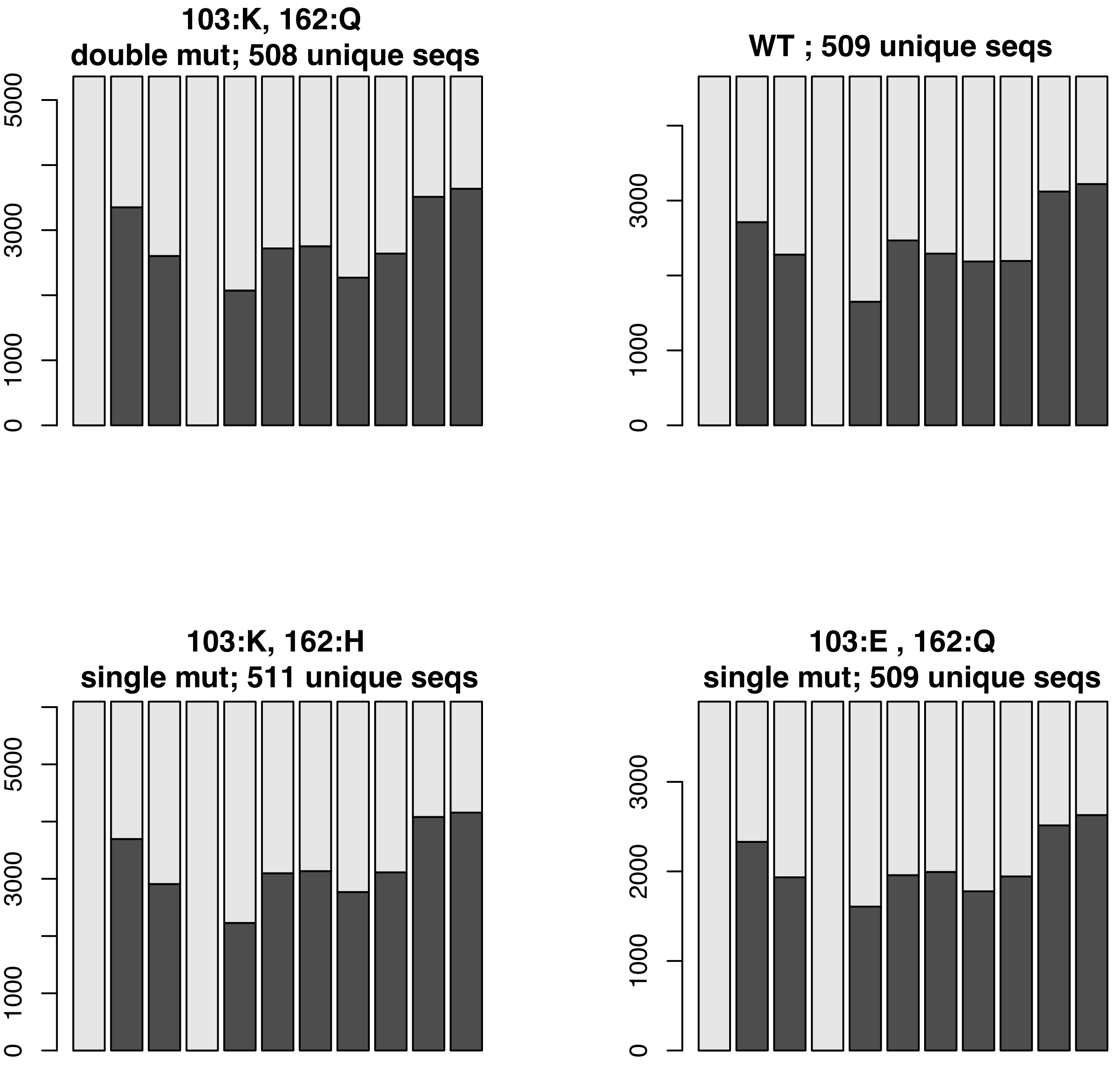
103:K, 162:Q
double mut; 508 unique seqs
0
1000
3000
5000
WT ; 509 unique seqs
0
1000
3000
103:K, 162:H
single mut; 511 unique seqs
0
1000
3000
5000
103:E , 162:Q
single mut; 509 unique seqs
0
1000
2000
3000

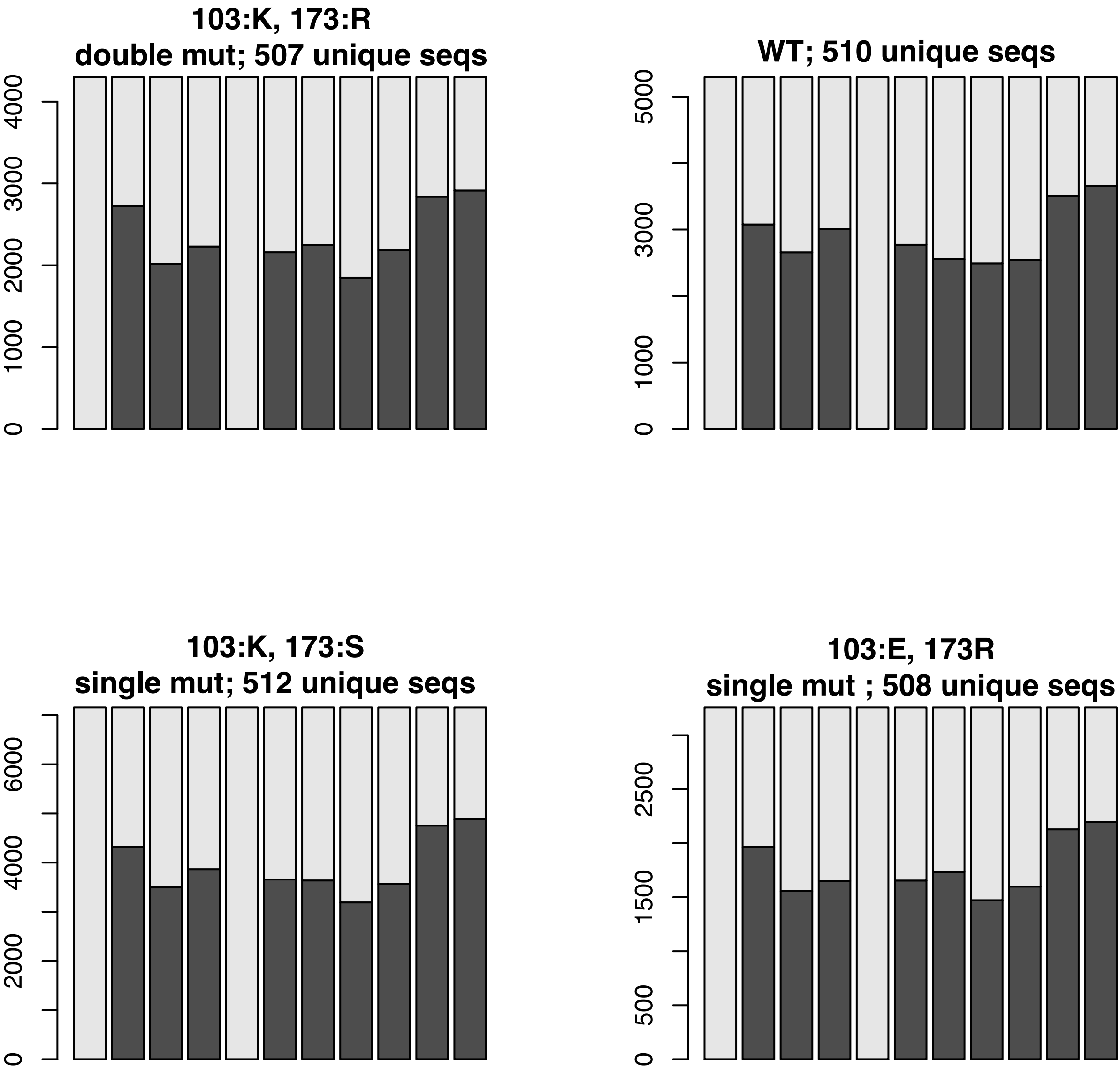
103:K, 173:R
double mut; 507 unique seqs
0
1000
2000
3000
4000
WT; 510 unique seqs
0
1000
3000
5000
103:K, 173:S
single mut; 512 unique seqs
0
2000
4000
6000
103:E, 173R
single mut ; 508 unique seqs
0
500
1500
2500

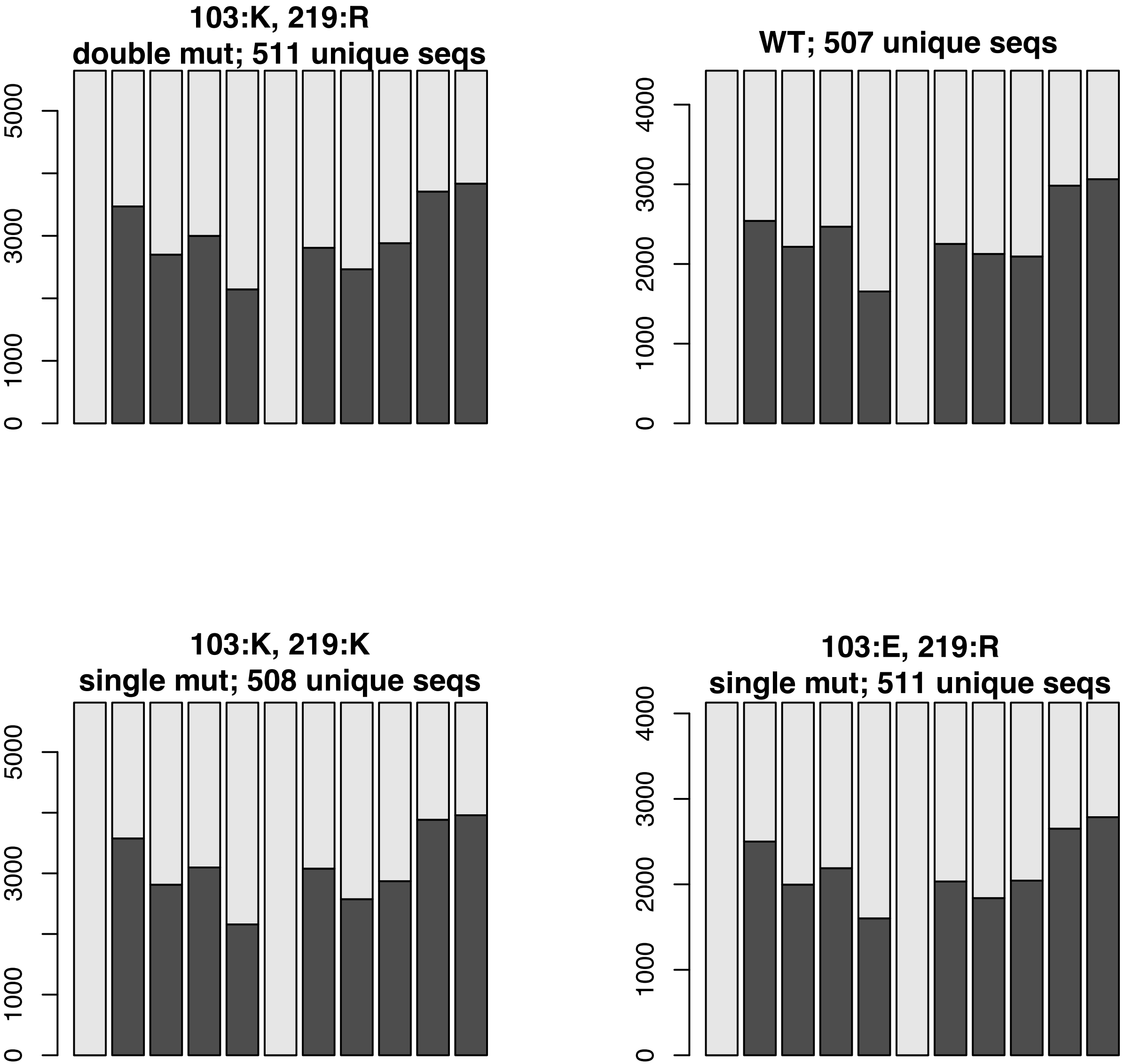
103:K, 219:R
double mut; 511 unique seqs
0
1000
3000
5000
WT; 507 unique seqs
0
1000
2000
3000
4000
103:K, 219:K
single mut; 508 unique seqs
0
1000
3000
5000
103:E, 219:R
single mut; 511 unique seqs
0
1000
2000
3000
4000

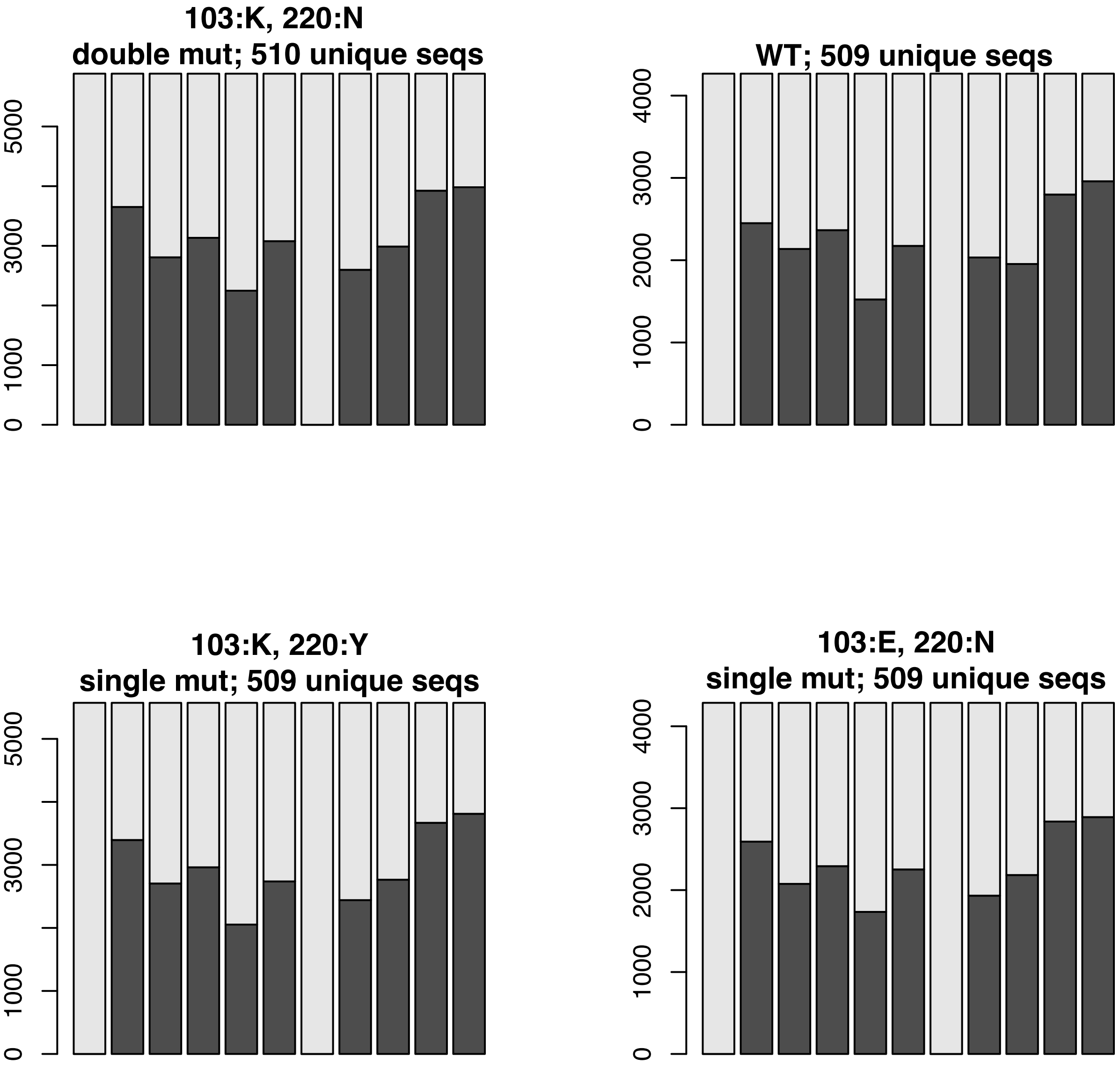
103:K, 220:N
double mut; 510 unique seqs
0
1000
3000
5000
WT; 509 unique seqs
0
1000
2000
3000
4000
103:K, 220:Y
single mut; 509 unique seqs
0
1000
3000
5000
103:E, 220:N
single mut; 509 unique seqs
0
1000
2000
3000
4000

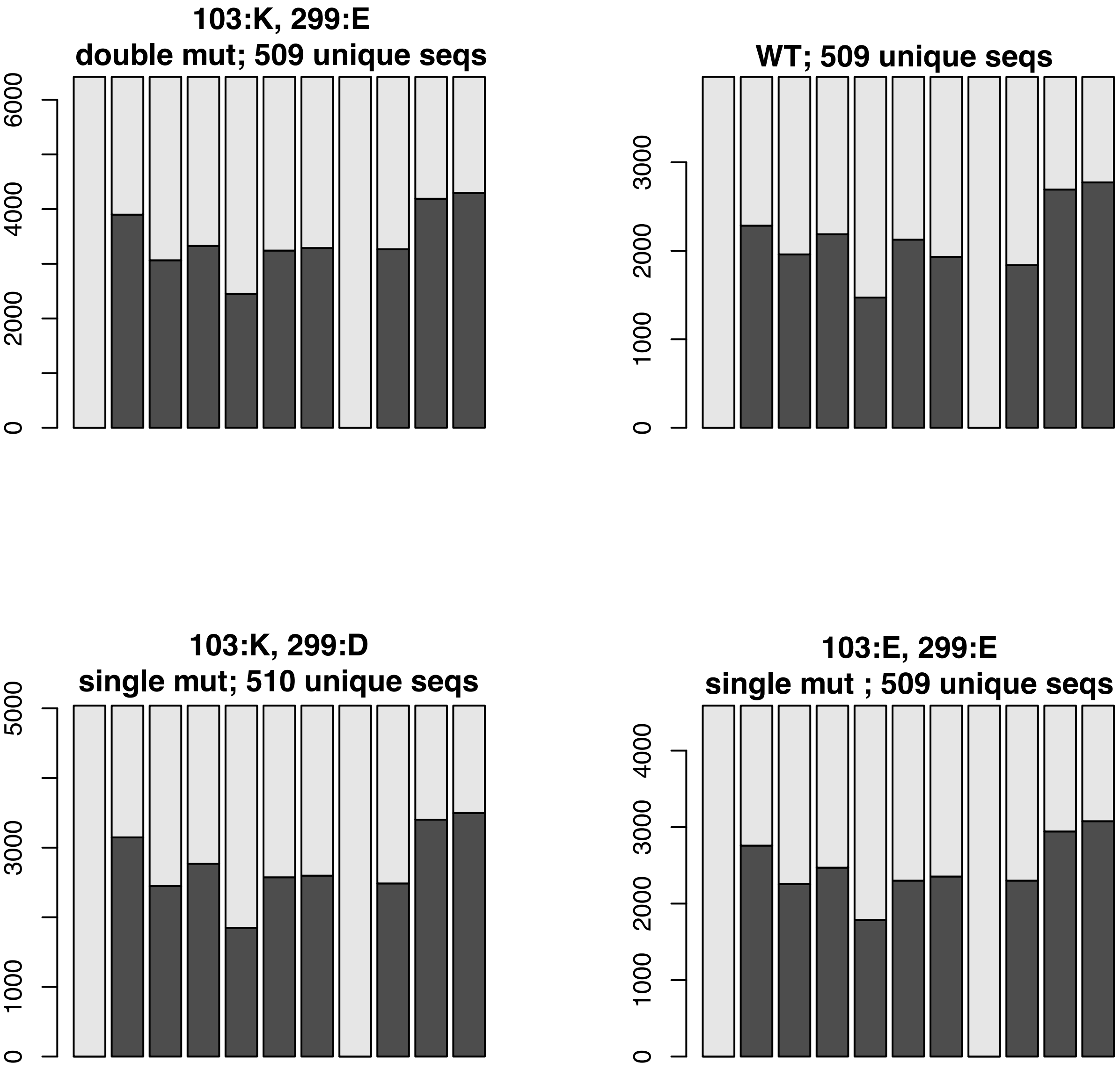

103:K, 299:E
double mut; 509 unique seqs
0
2000
4000
6000
WT; 509 unique seqs
0
1000
2000
3000
103:K, 299:D
single mut; 510 unique seqs
0
1000
3000
5000
103:E, 299:E
single mut ; 509 unique seqs
0
1000
2000
3000
4000

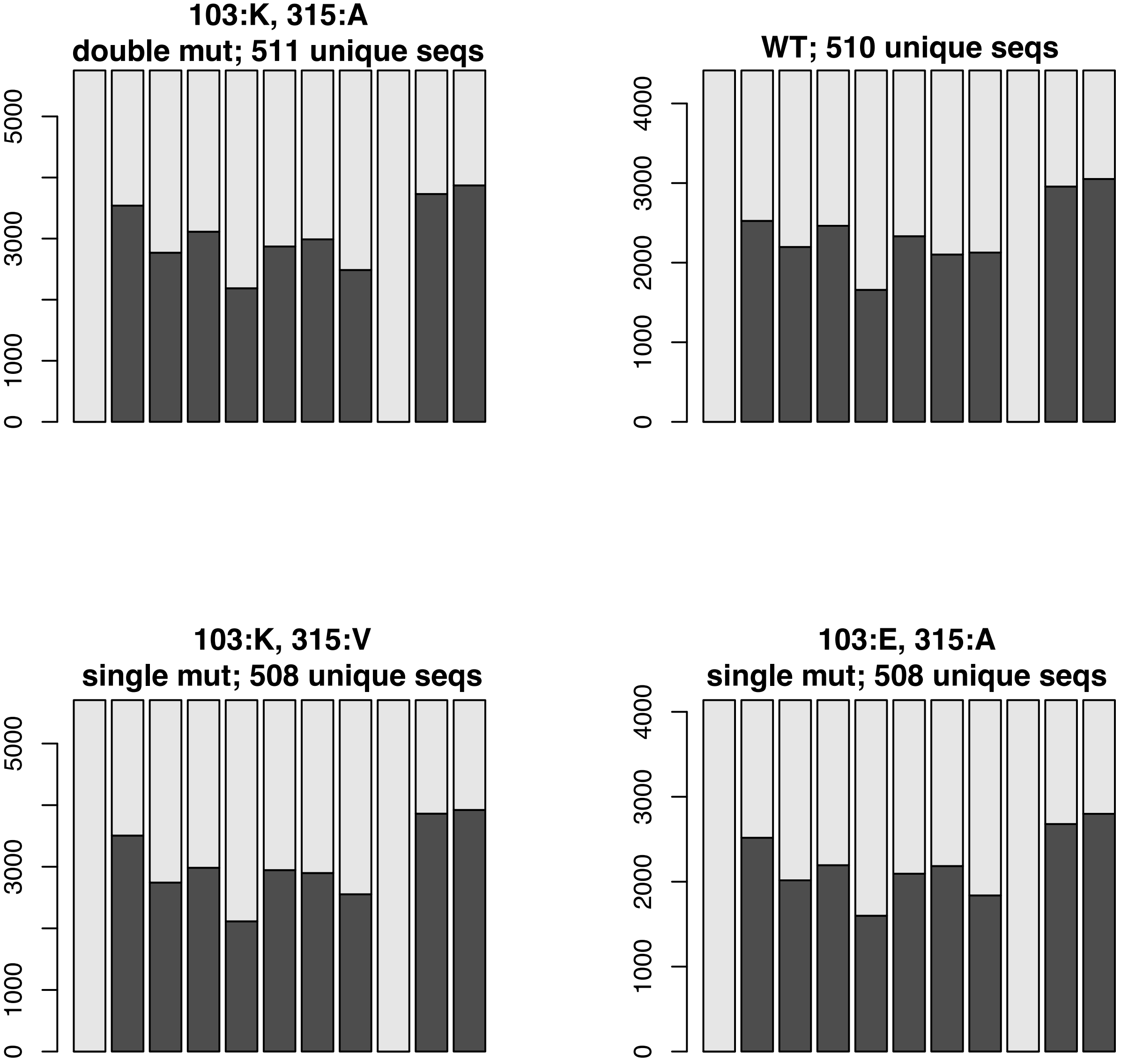
103:K, 315:A
double mut; 511 unique seqs
0
1000
3000
5000
WT; 510 unique seqs
0
1000
2000
3000
4000
103:K, 315:V
single mut; 508 unique seqs
0
1000
3000
5000
103:E, 315:A
single mut; 508 unique seqs
0
1000
2000
3000
4000

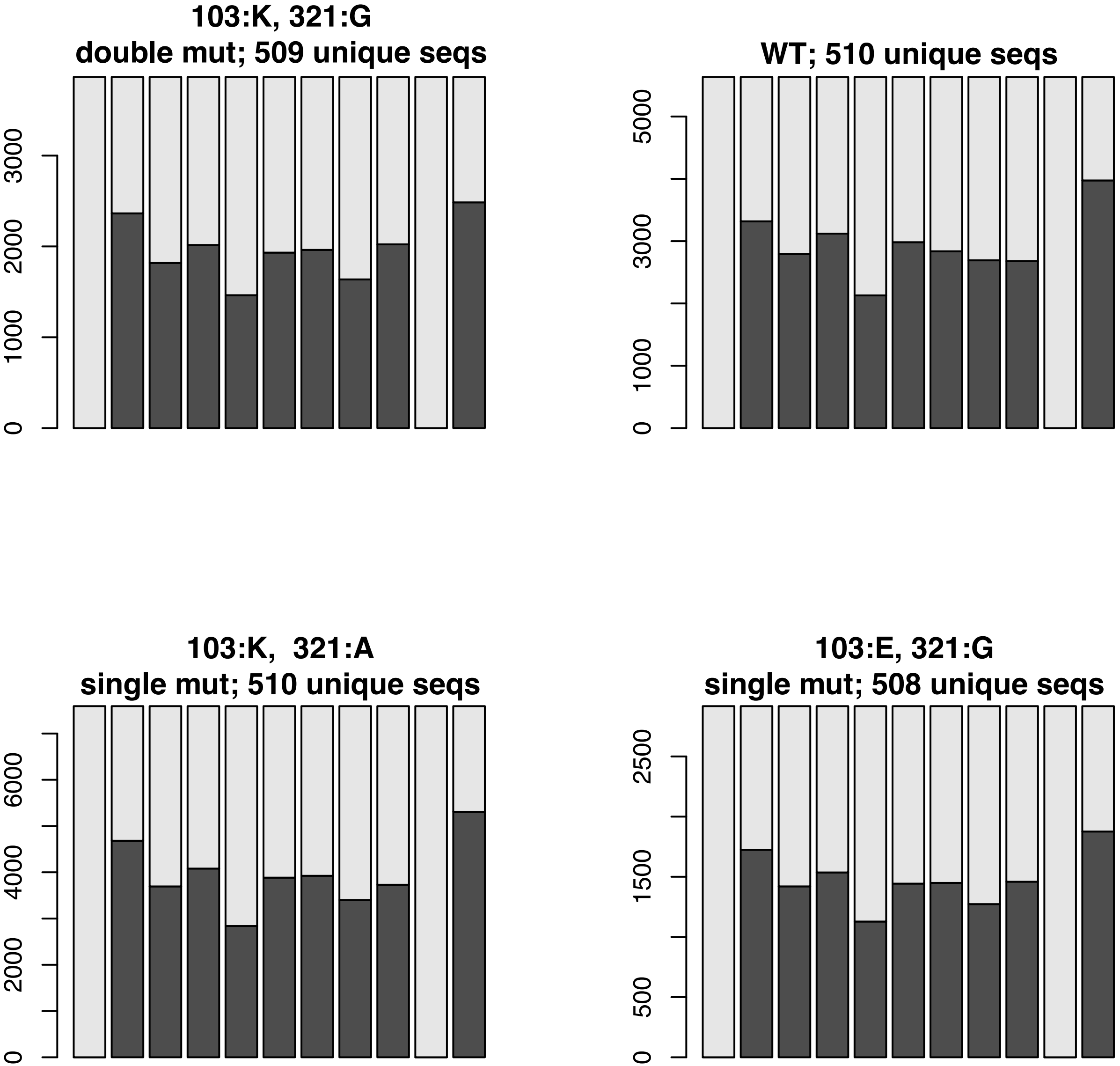
103:K, 321:G
double mut; 509 unique seqs
0
1000
2000
3000
WT; 510 unique seqs
0
1000
3000
5000
103:K, 321:A
single mut; 510 unique seqs
0
2000
4000
6000
103:E, 321:G
single mut; 508 unique seqs
0
500
1500
2500

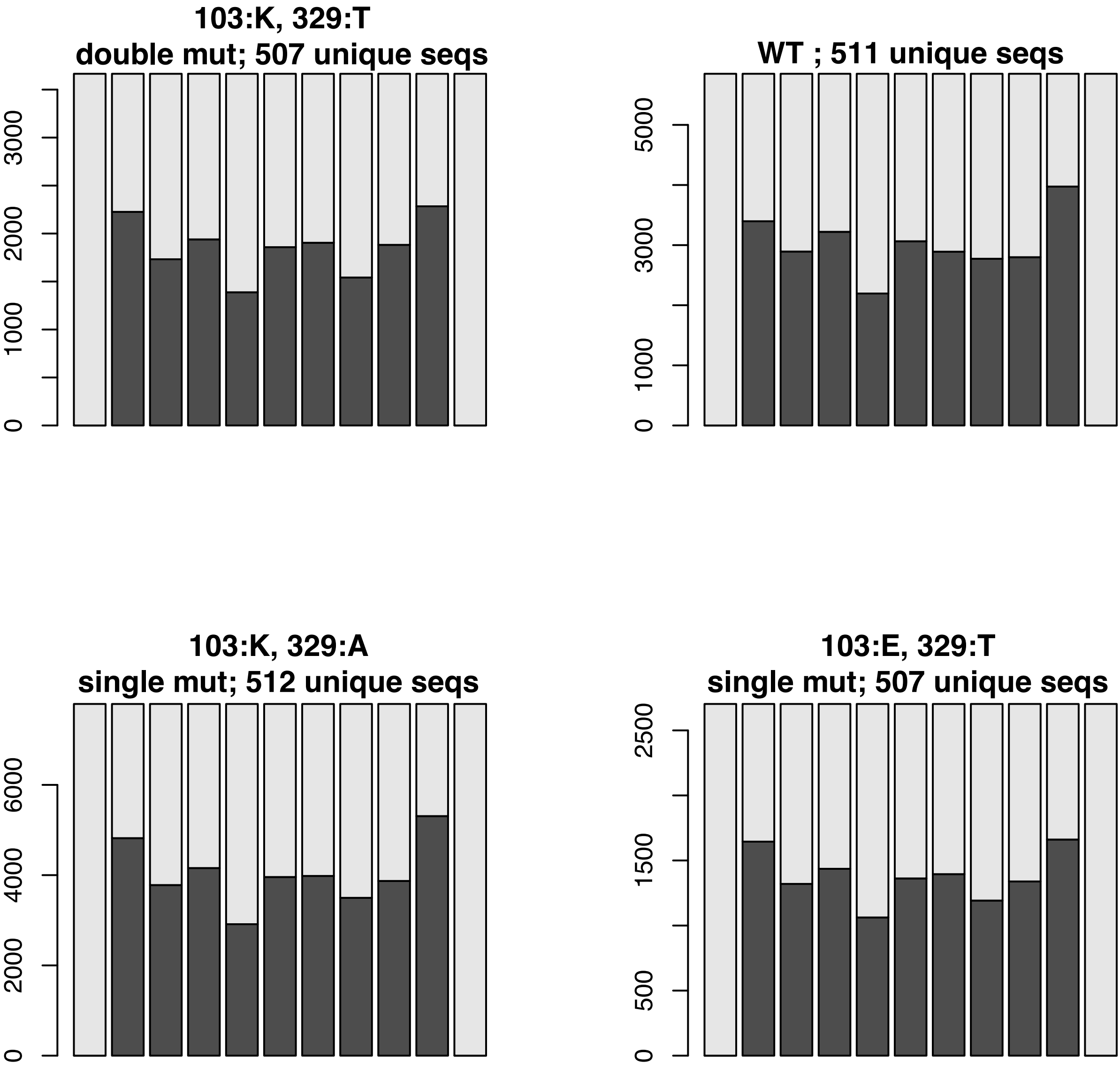
103:K, 329:T
double mut; 507 unique seqs
0
1000
2000
3000
WT ; 511 unique seqs
0
1000
3000
5000
103:K, 329:A
single mut; 512 unique seqs
0
2000
4000
6000
103:E, 329:T
single mut; 507 unique seqs
0
500
1500
2500

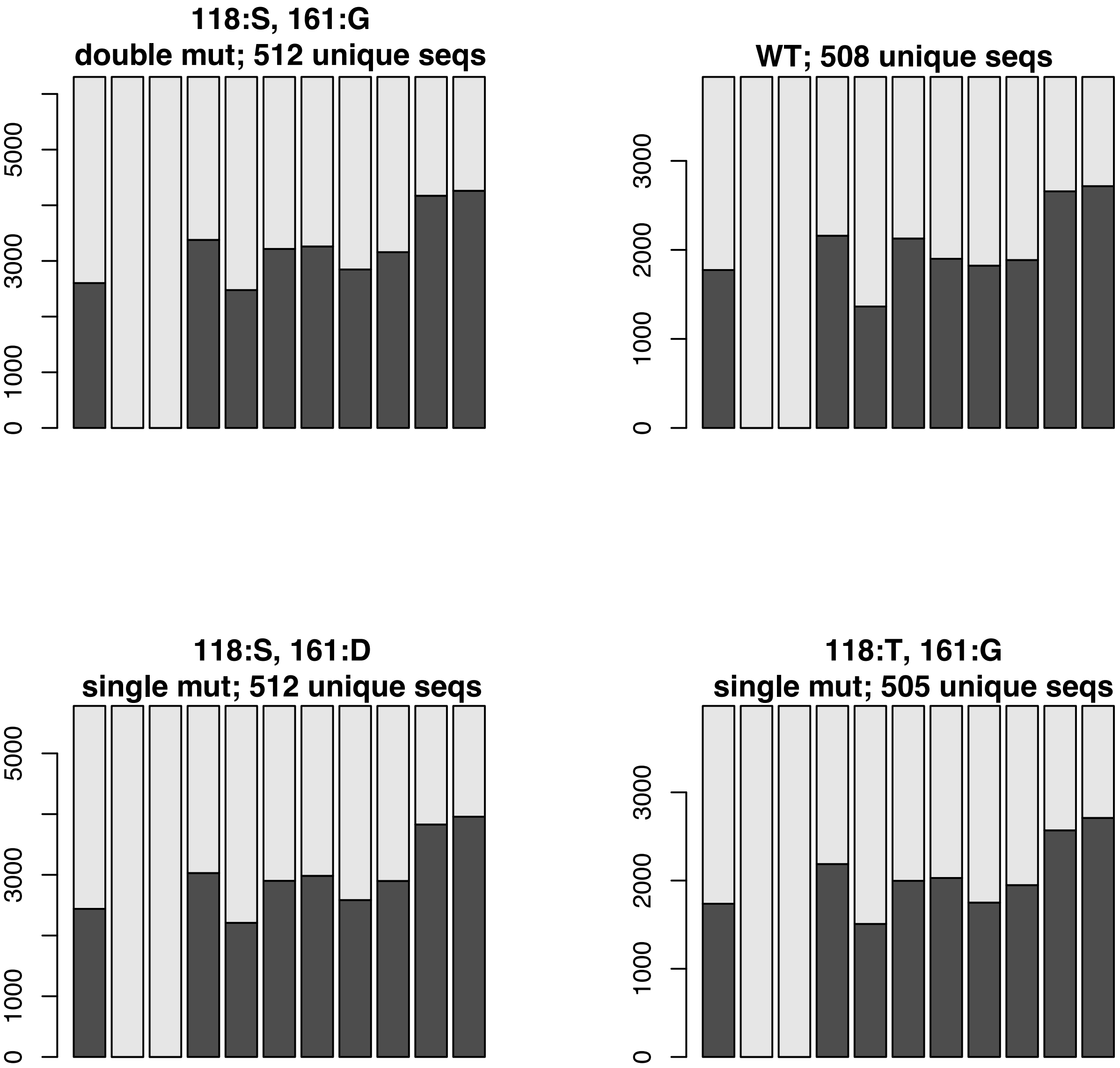

118:S, 161:G
double mut; 512 unique seqs
0
1000
3000
5000
WT; 508 unique seqs
0
1000
2000
3000
118:S, 161:D
single mut; 512 unique seqs
0
1000
3000
5000
118:T, 161:G
single mut; 505 unique seqs
0
1000
2000
3000

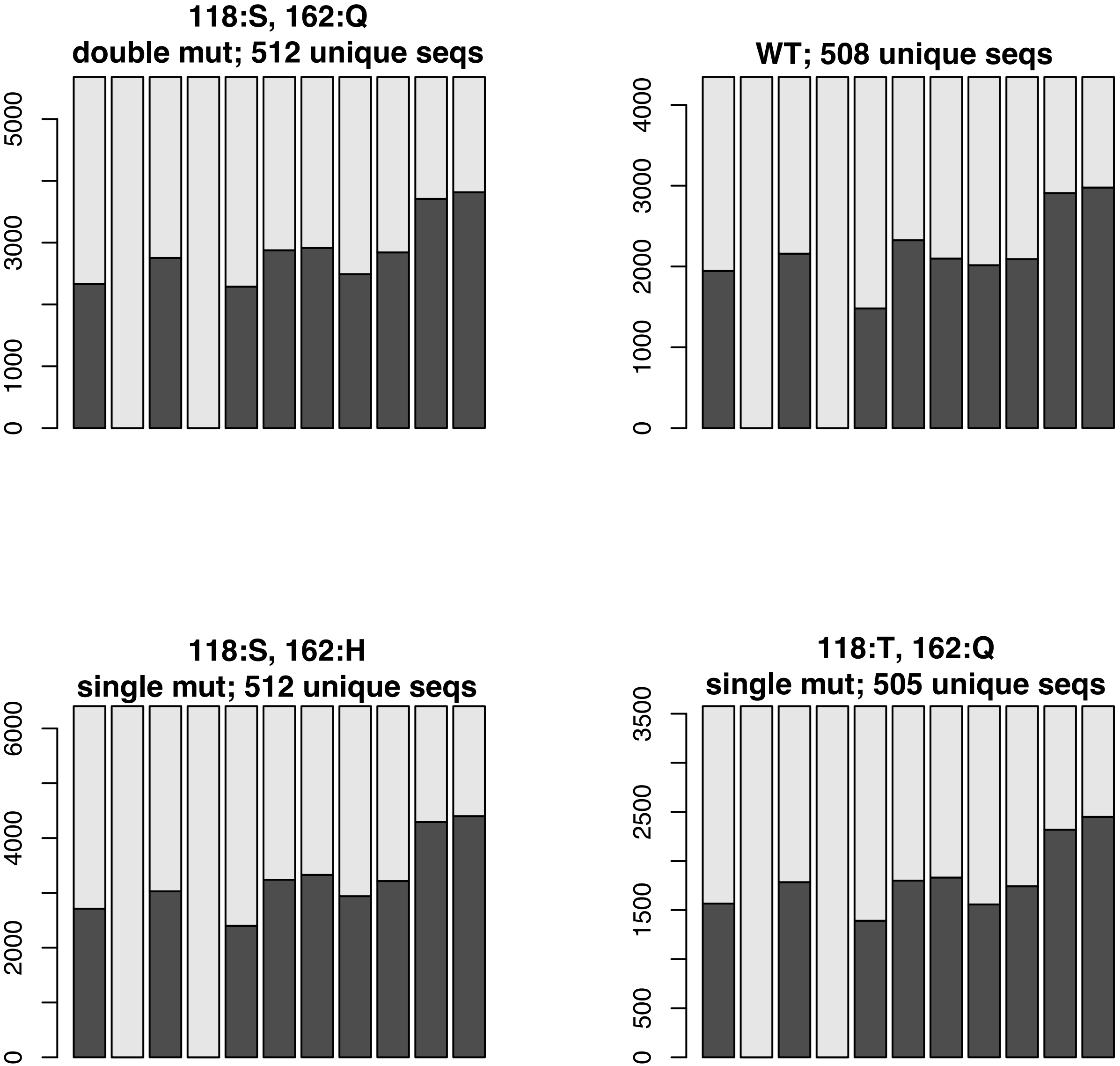

118:S, 162:Q
double mut; 512 unique seqs
0
1000
3000
5000
WT; 508 unique seqs
0
1000
2000
3000
4000
118:S, 162:H
single mut; 512 unique seqs
0
2000
4000
6000
118:T, 162:Q
single mut; 505 unique seqs
0
500
1500
2500
3500

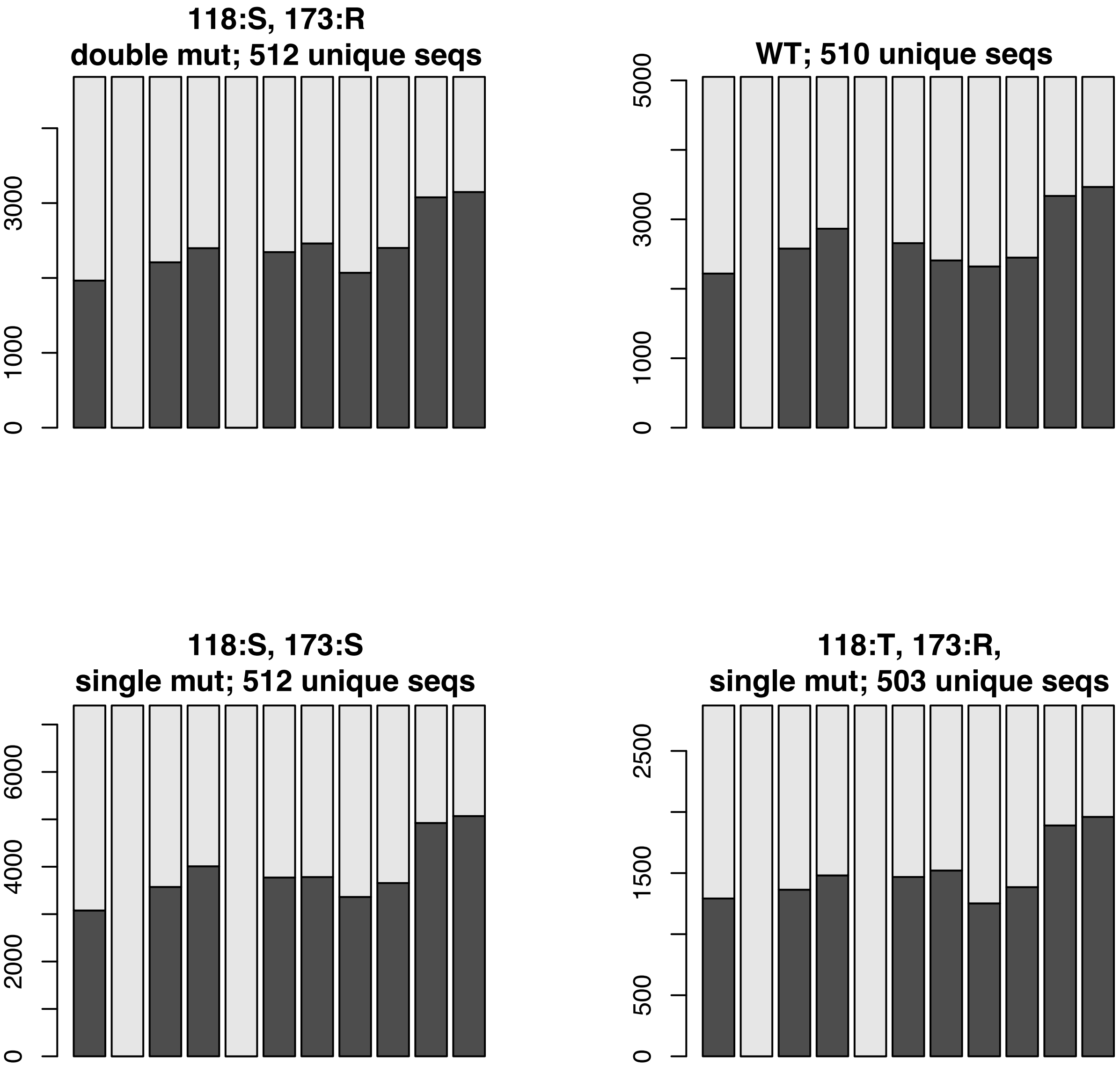
118:S, 173:R
double mut; 512 unique seqs
0
1000
3000
WT; 510 unique seqs
0
1000
3000
5000
118:S, 173:S
single mut; 512 unique seqs
0
2000
4000
6000
118:T, 173:R,
single mut; 503 unique seqs
0
500
1500
2500

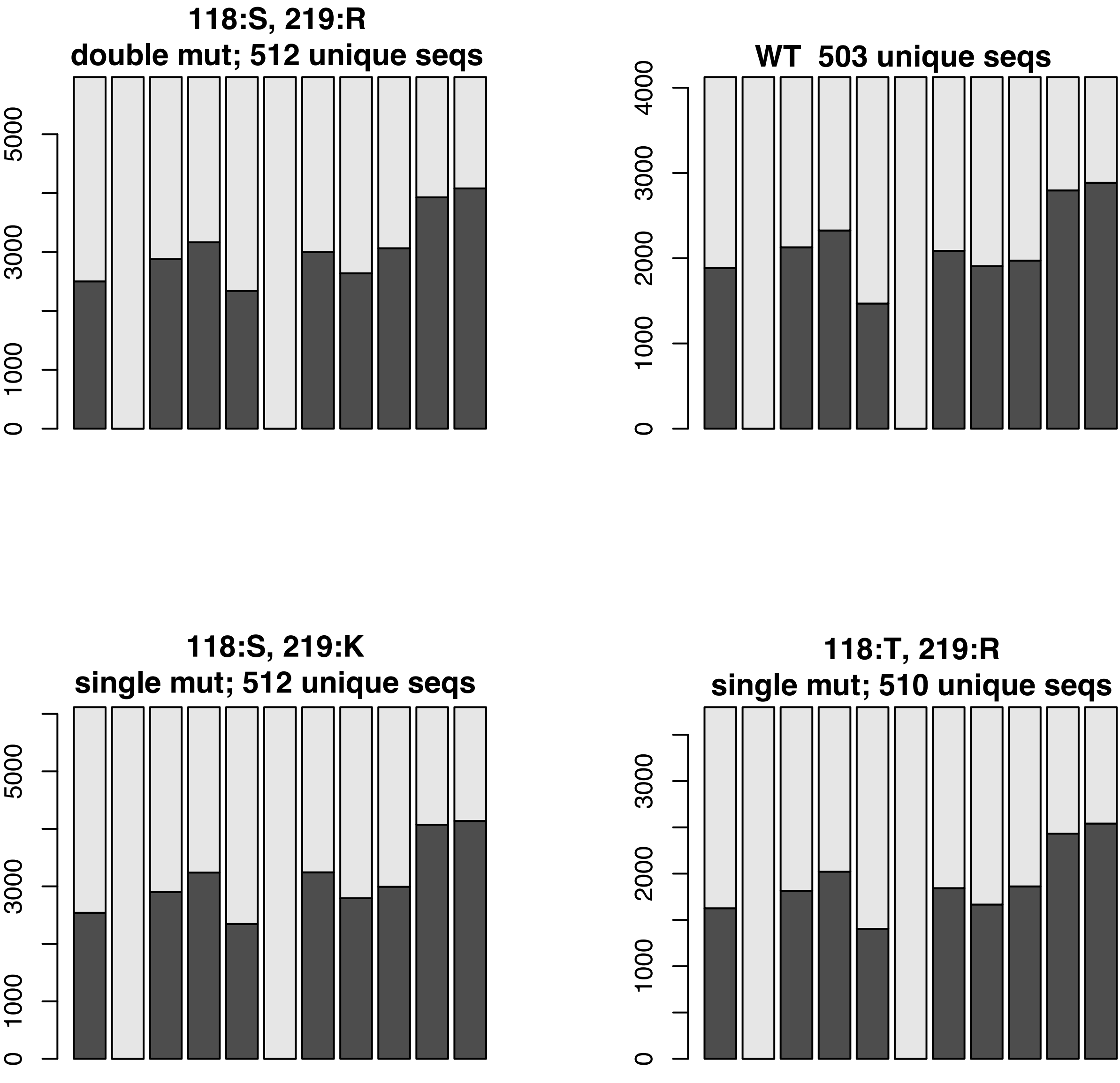
118:S, 219:R
double mut; 512 unique seqs
0
1000
3000
5000
WT 503 unique seqs
0
1000
2000
3000
4000
118:S, 219:K
single mut; 512 unique seqs
0
1000
3000
5000
118:T, 219:R
single mut; 510 unique seqs
0
1000
2000
3000

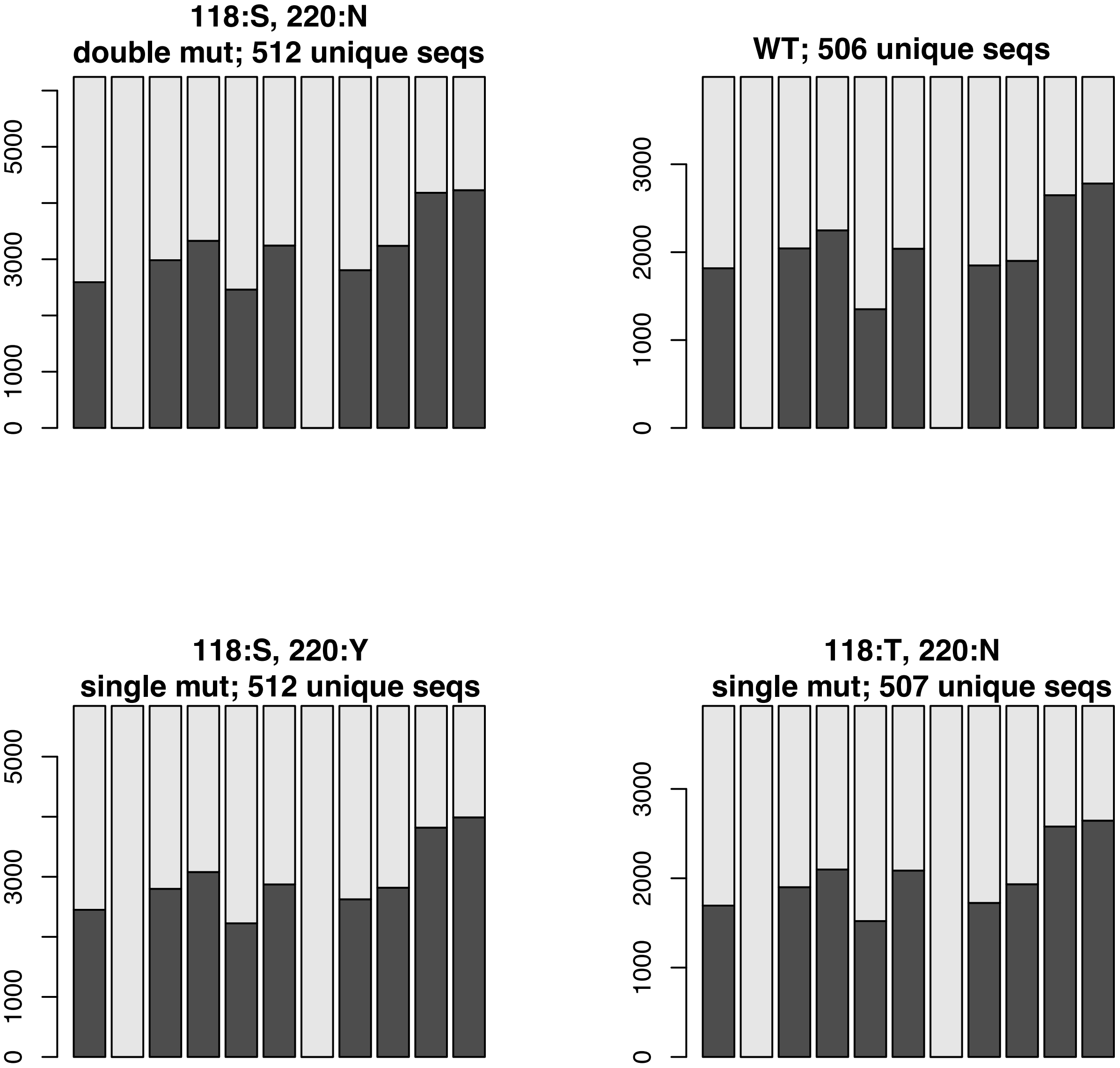

118:S, 220:N
double mut; 512 unique seqs
0
1000
3000
5000
WT; 506 unique seqs
0
1000
2000
3000
118:S, 220:Y
single mut; 512 unique seqs
0
1000
3000
5000
118:T, 220:N
single mut; 507 unique seqs
0
1000
2000
3000

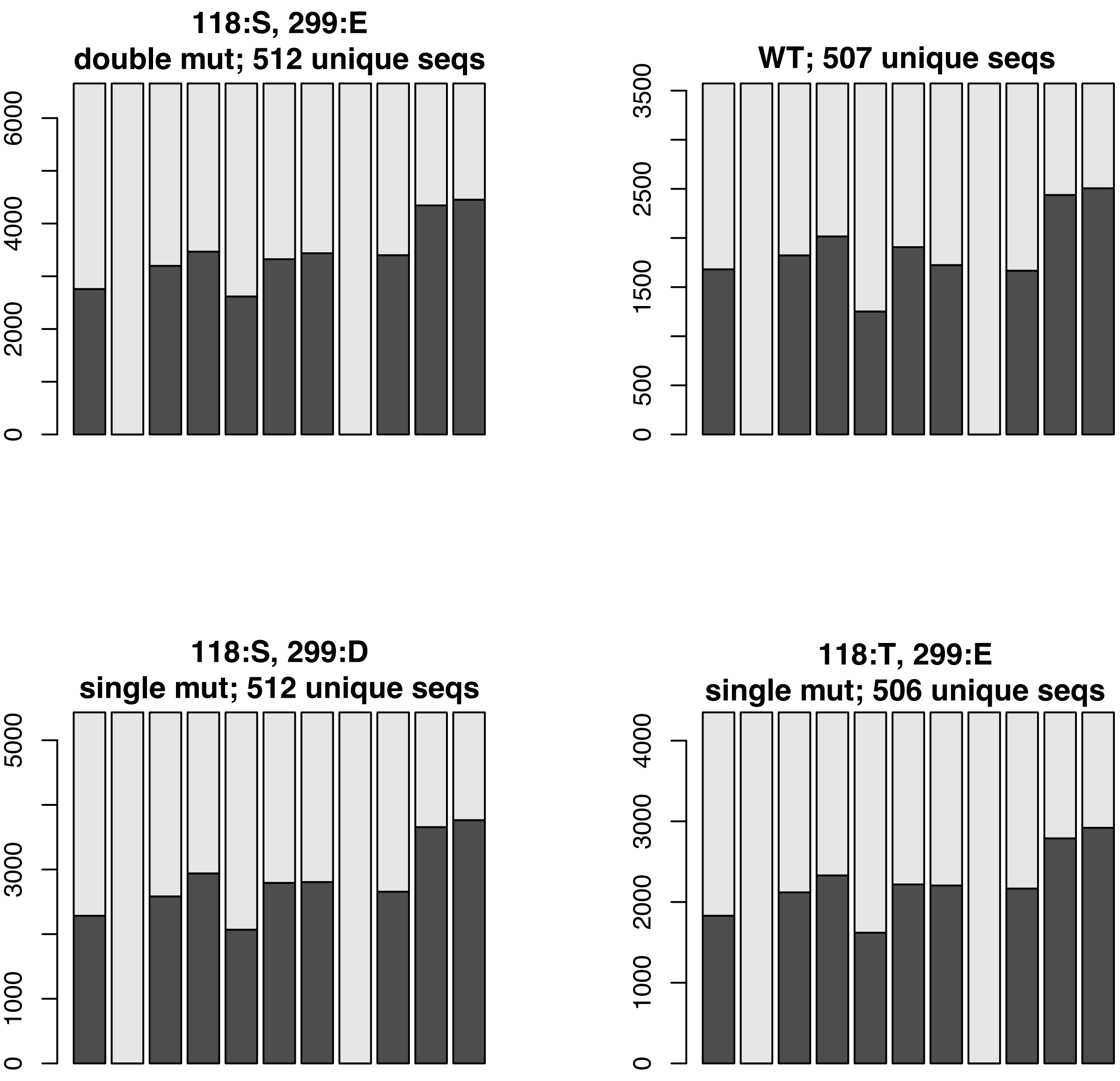
118:S, 299:E
double mut; 512 unique seqs
0
2000
4000
6000
WT; 507 unique seqs
0
500
1500
2500
3500
118:S, 299:D
single mut; 512 unique seqs
0
1000
3000
5000
118:T, 299:E
single mut; 506 unique seqs
0
1000
2000
3000
4000

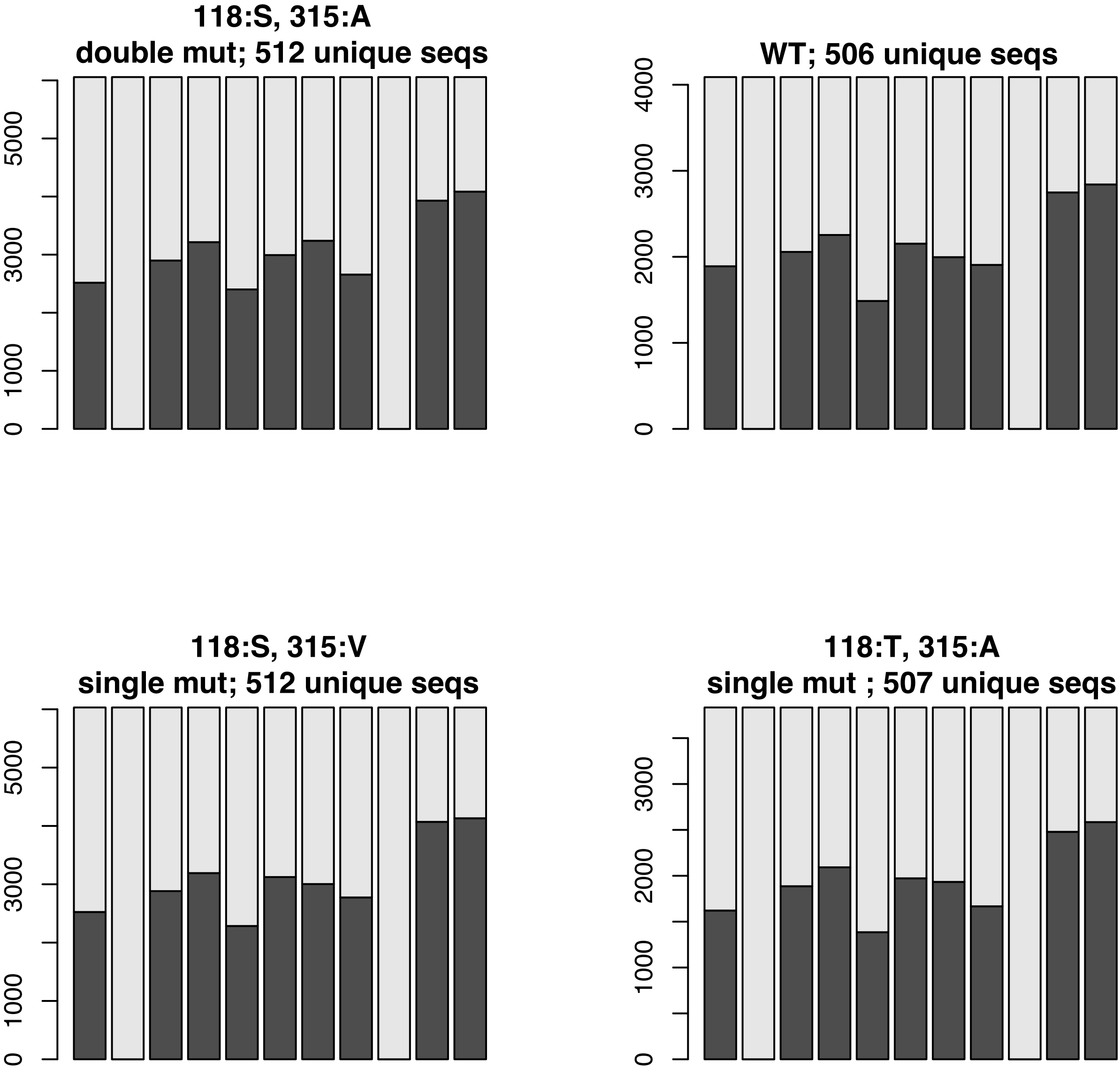
118:S, 315:A
double mut; 512 unique seqs
0
1000
3000
5000
WT; 506 unique seqs
0
1000
2000
3000
4000
118:S, 315:V
single mut; 512 unique seqs
0
1000
3000
5000
118:T, 315:A
single mut ; 507 unique seqs
0
1000
2000
3000

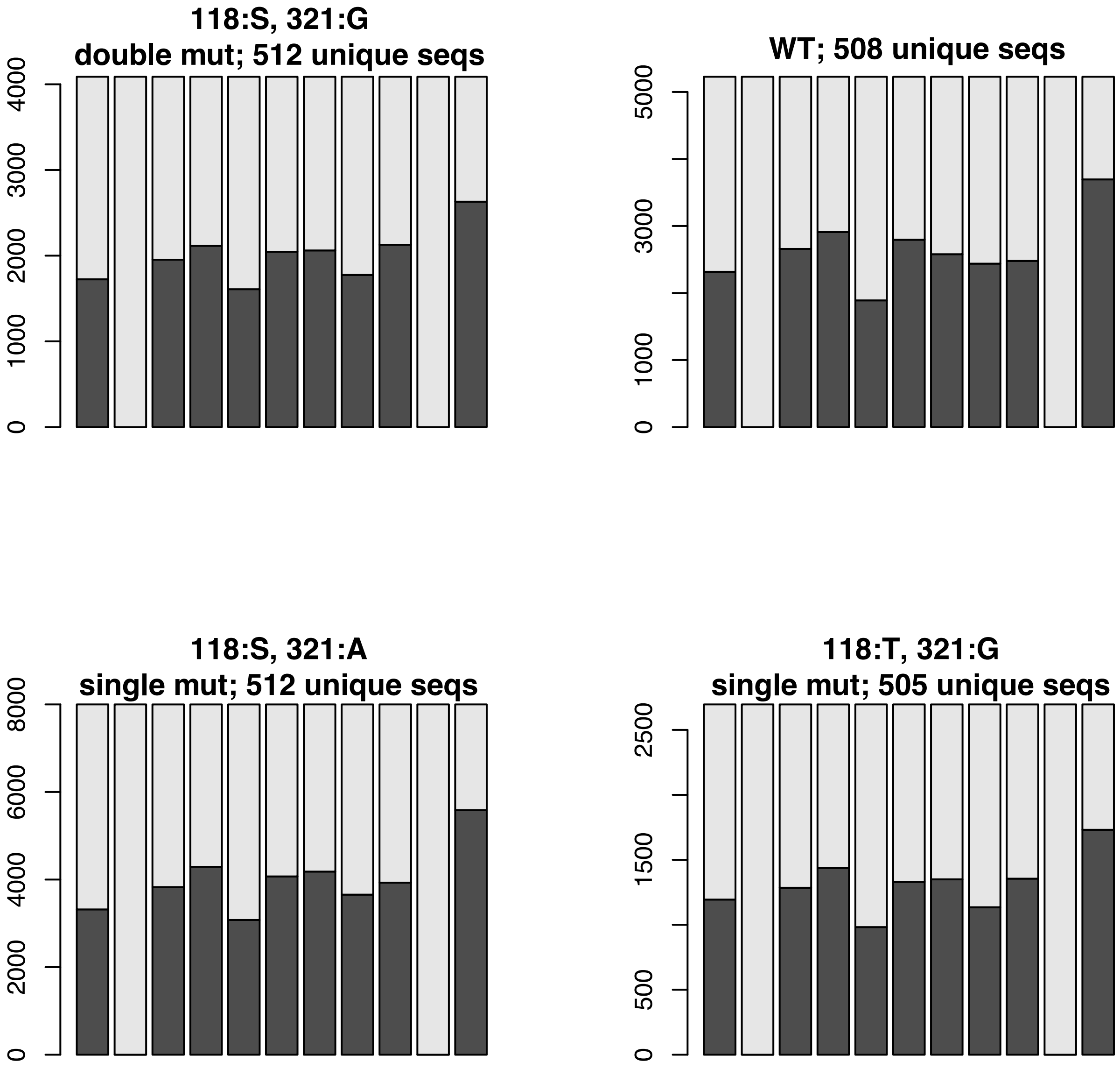
118:S, 321:G
double mut; 512 unique seqs
0
1000
2000
3000
4000
WT; 508 unique seqs
0
1000
3000
5000
118:S, 321:A
single mut; 512 unique seqs
0
2000
4000
6000
8000
118:T, 321:G
single mut; 505 unique seqs
0
500
1500
2500

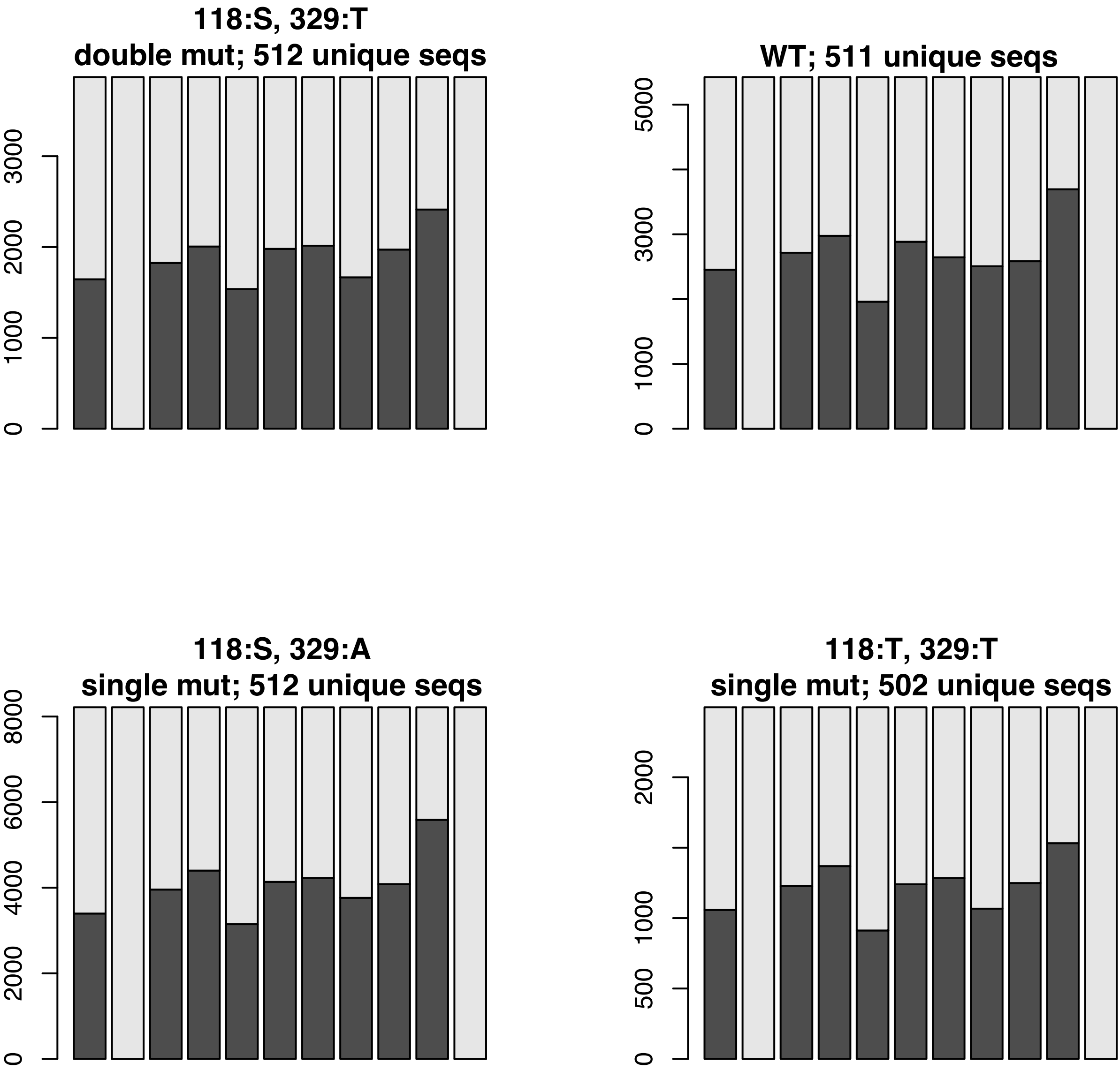
118:S, 329:T
double mut; 512 unique seqs
0
1000
2000
3000
WT; 511 unique seqs
0
1000
3000
5000
118:S, 329:A
single mut; 512 unique seqs
0
2000
4000
6000
8000
118:T, 329:T
single mut; 502 unique seqs
0
500
1000
2000

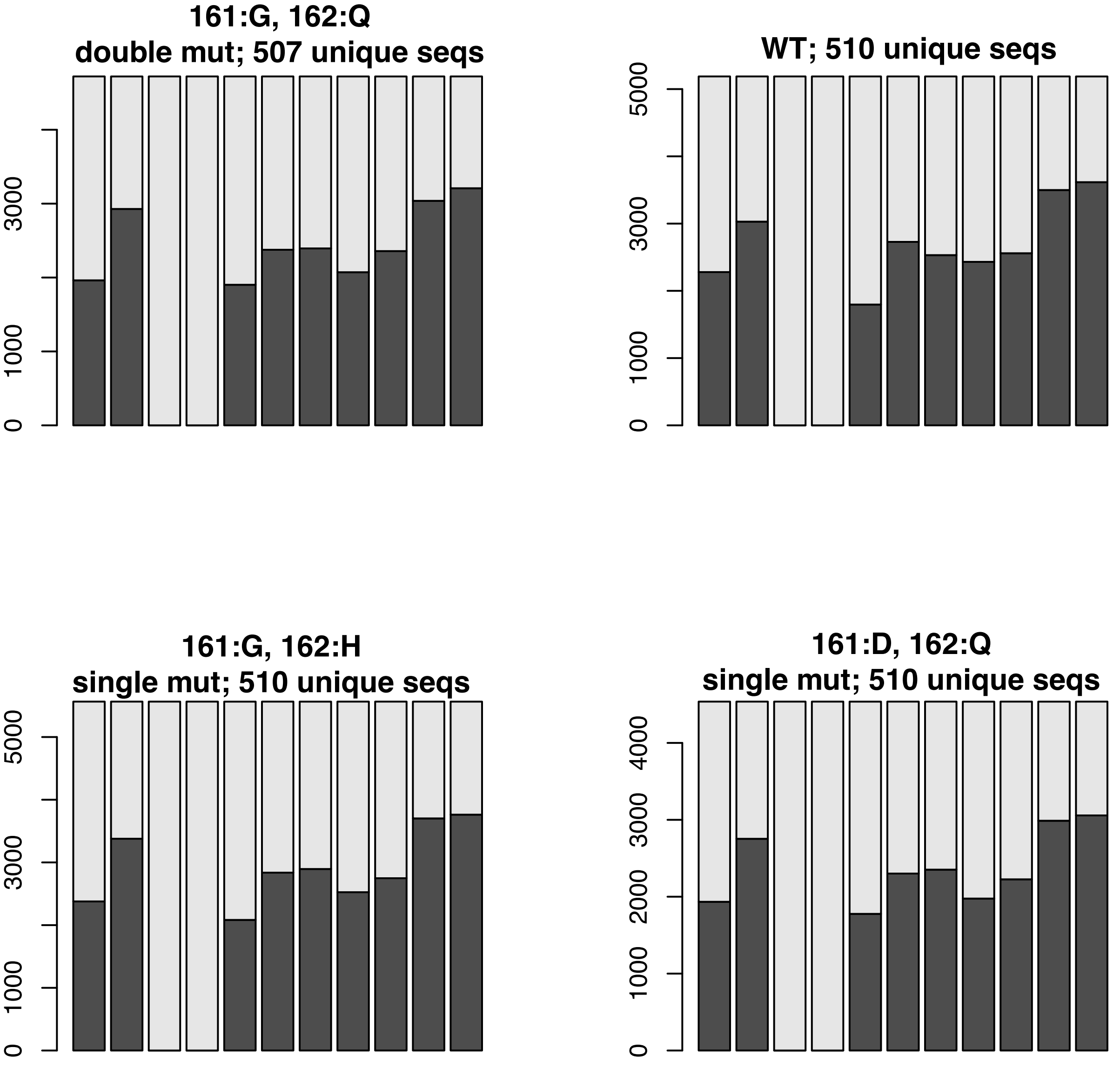
161:G, 162:Q
double mut; 507 unique seqs
0
1000
3000
WT; 510 unique seqs
0
1000
3000
5000
161:G, 162:H
single mut; 510 unique seqs
0
1000
3000
5000
161:D, 162:Q
single mut; 510 unique seqs
0
1000
2000
3000
4000

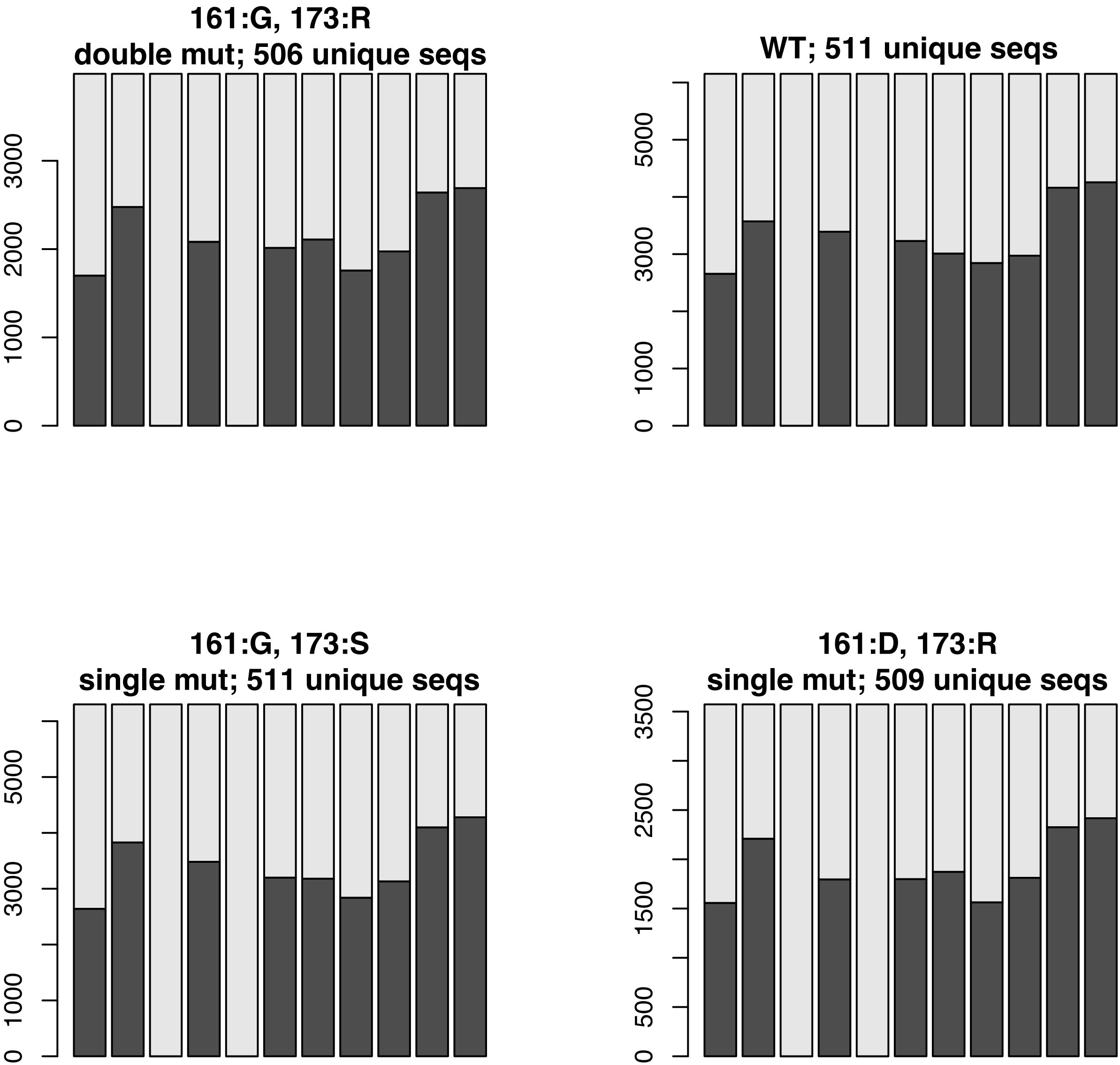
161:G, 173:R
double mut; 506 unique seqs
0
1000
2000
3000
WT; 511 unique seqs
0
1000
3000
5000
161:G, 173:S
single mut; 511 unique seqs
0
1000
3000
5000
161:D, 173:R
single mut; 509 unique seqs
0
500
1500
2500
3500

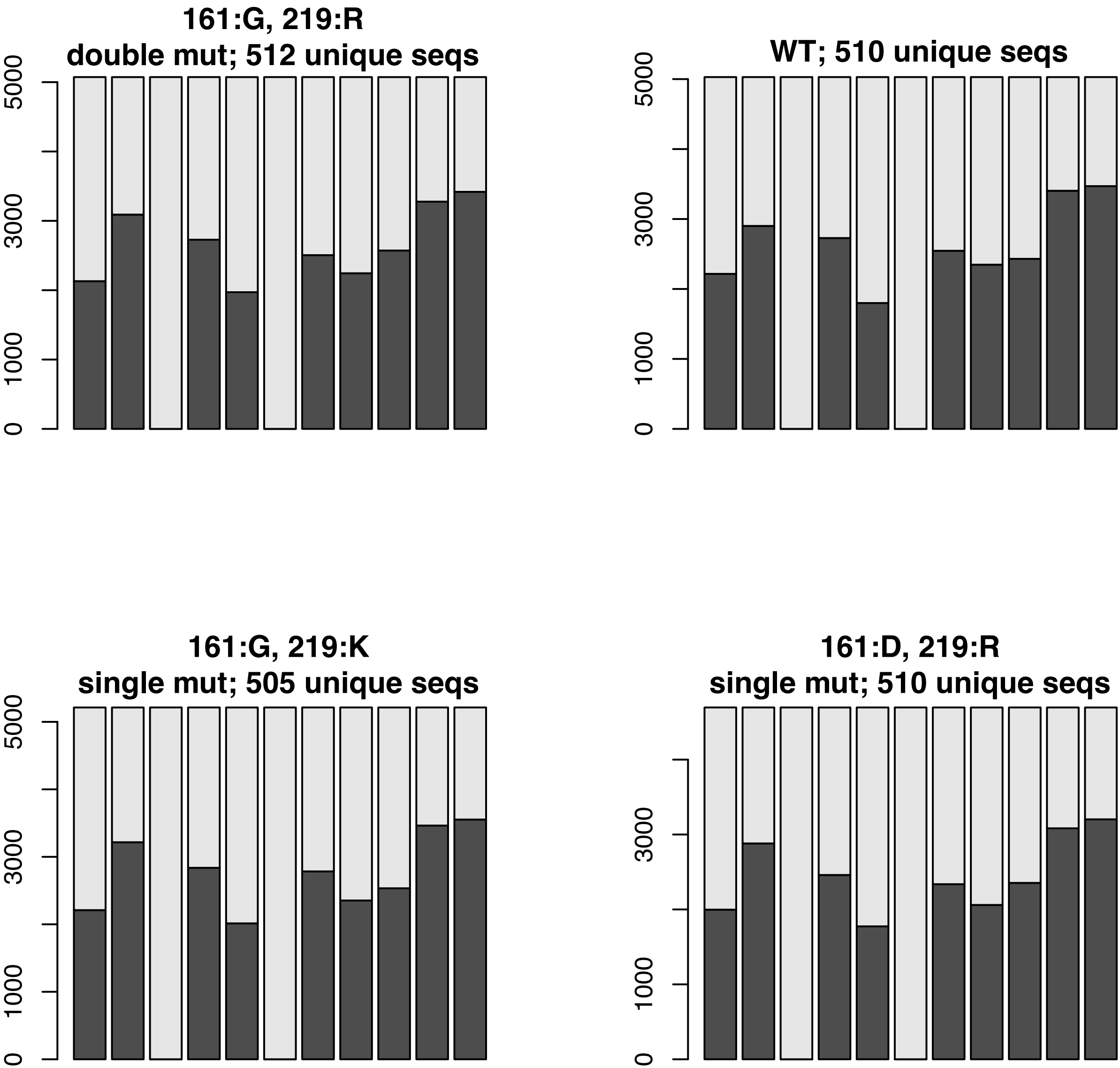

161:G, 219:R
double mut; 512 unique seqs
0
1000
3000
5000
WT; 510 unique seqs
0
1000
3000
5000
161:G, 219:K
single mut; 505 unique seqs
0
1000
3000
5000
161:D, 219:R
single mut; 510 unique seqs
0
1000
3000

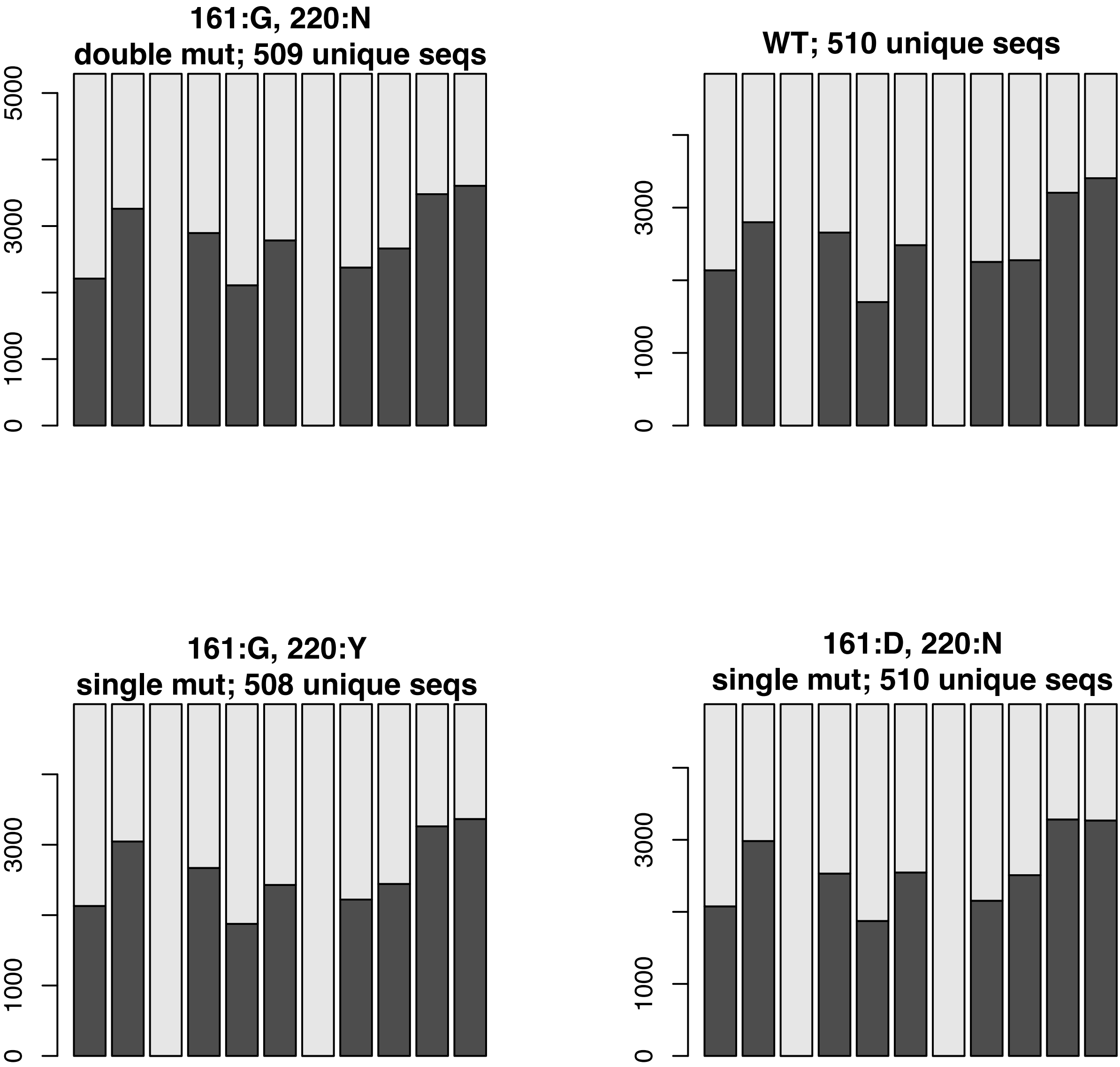

161:G, 220:N
double mut; 509 unique seqs
0
1000
3000
5000
WT; 510 unique seqs
0
1000
3000
161:G, 220:Y
single mut; 508 unique seqs
0
1000
3000
161:D, 220:N
single mut; 510 unique seqs
0
1000
3000

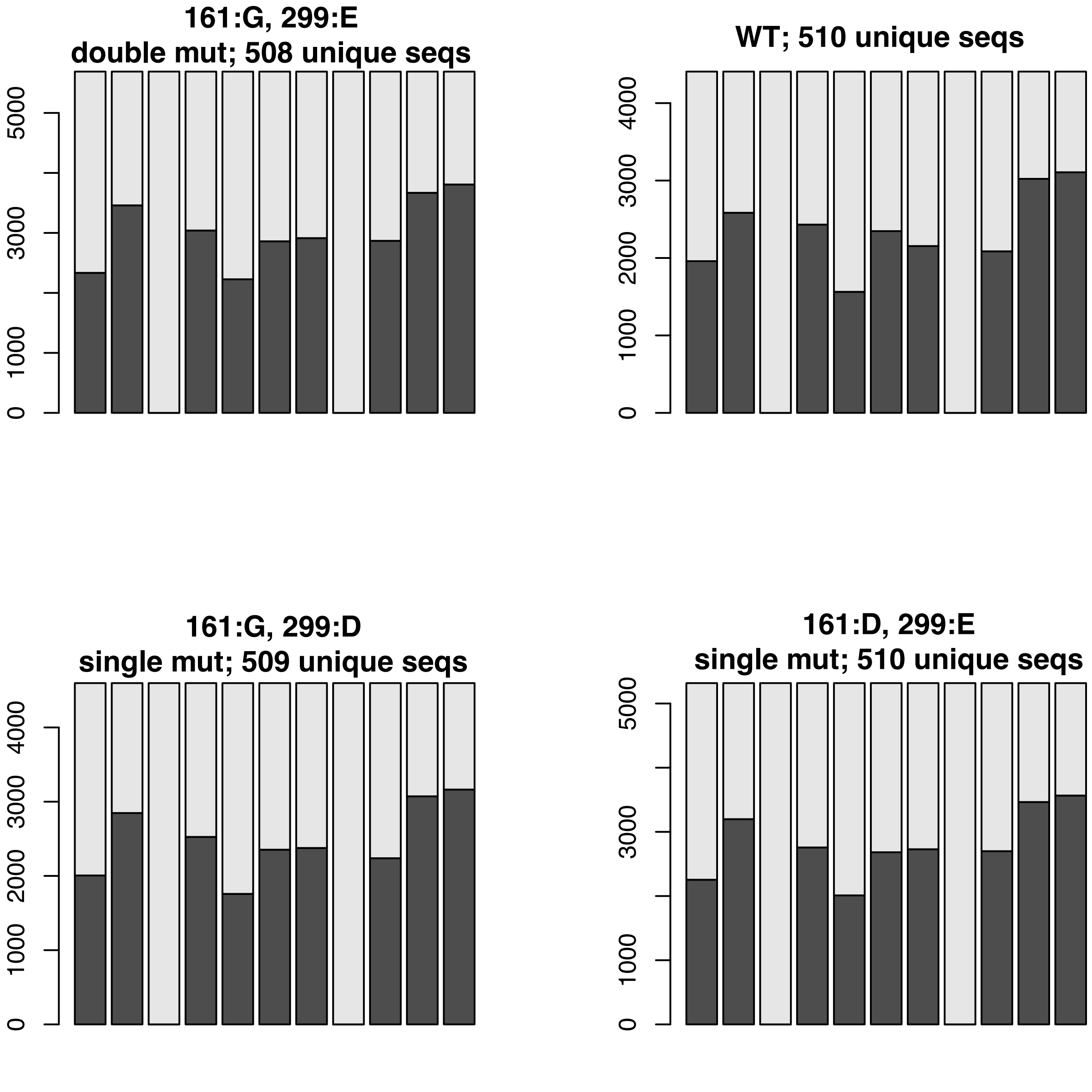
161:G, 299:E
double mut; 508 unique seqs
0
1000
3000
5000
WT; 510 unique seqs
0
1000
2000
3000
4000
161:G, 299:D
single mut; 509 unique seqs
0
1000
2000
3000
4000
161:D, 299:E
single mut; 510 unique seqs
0
1000
3000
5000

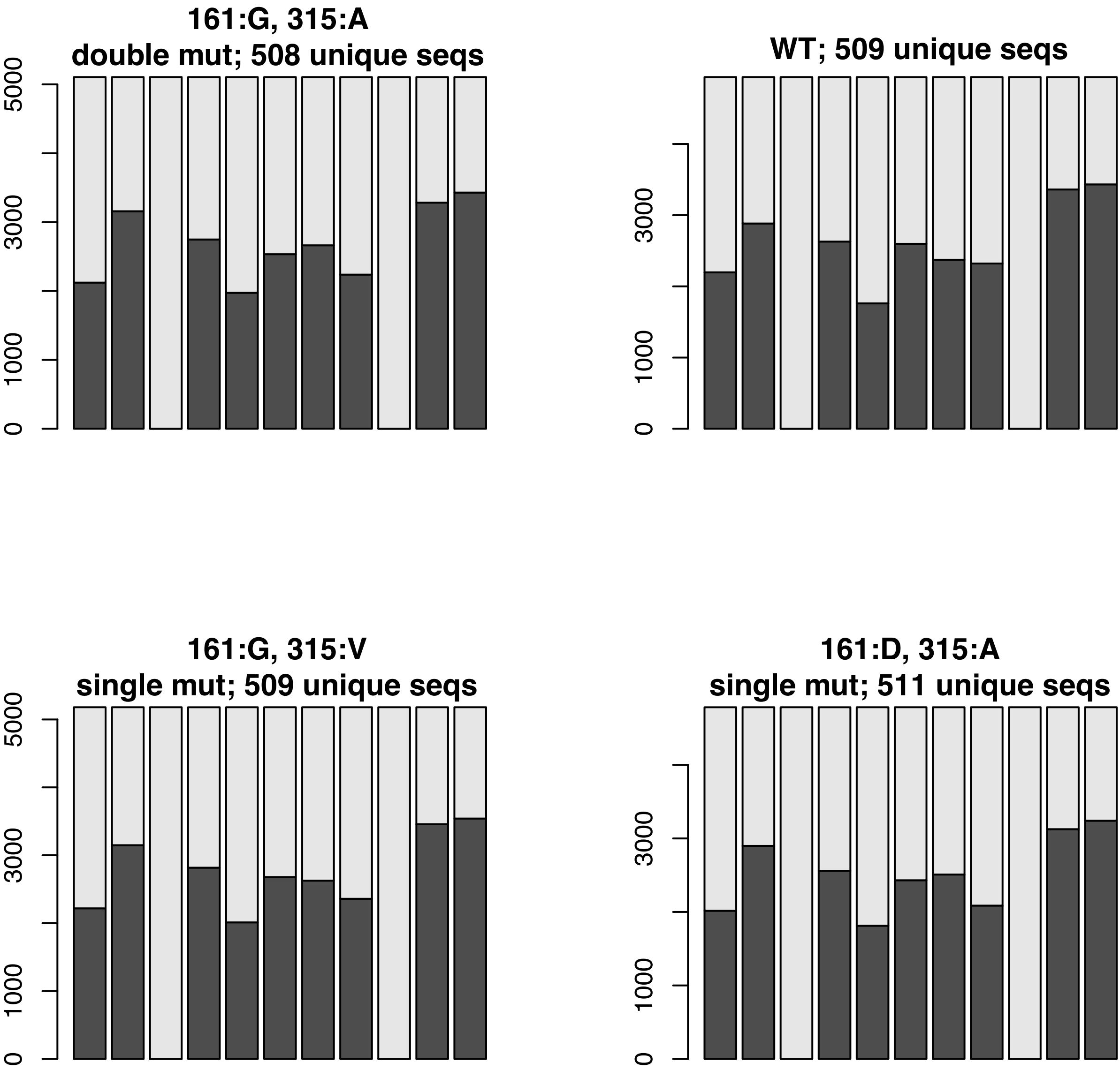
161:G, 315:A
double mut; 508 unique seqs
WT; 509 unique seqs
161:G, 315:V
single mut; 509 unique seqs
161:D, 315:A
single mut; 511 unique seqs
0
1000
3000
5000

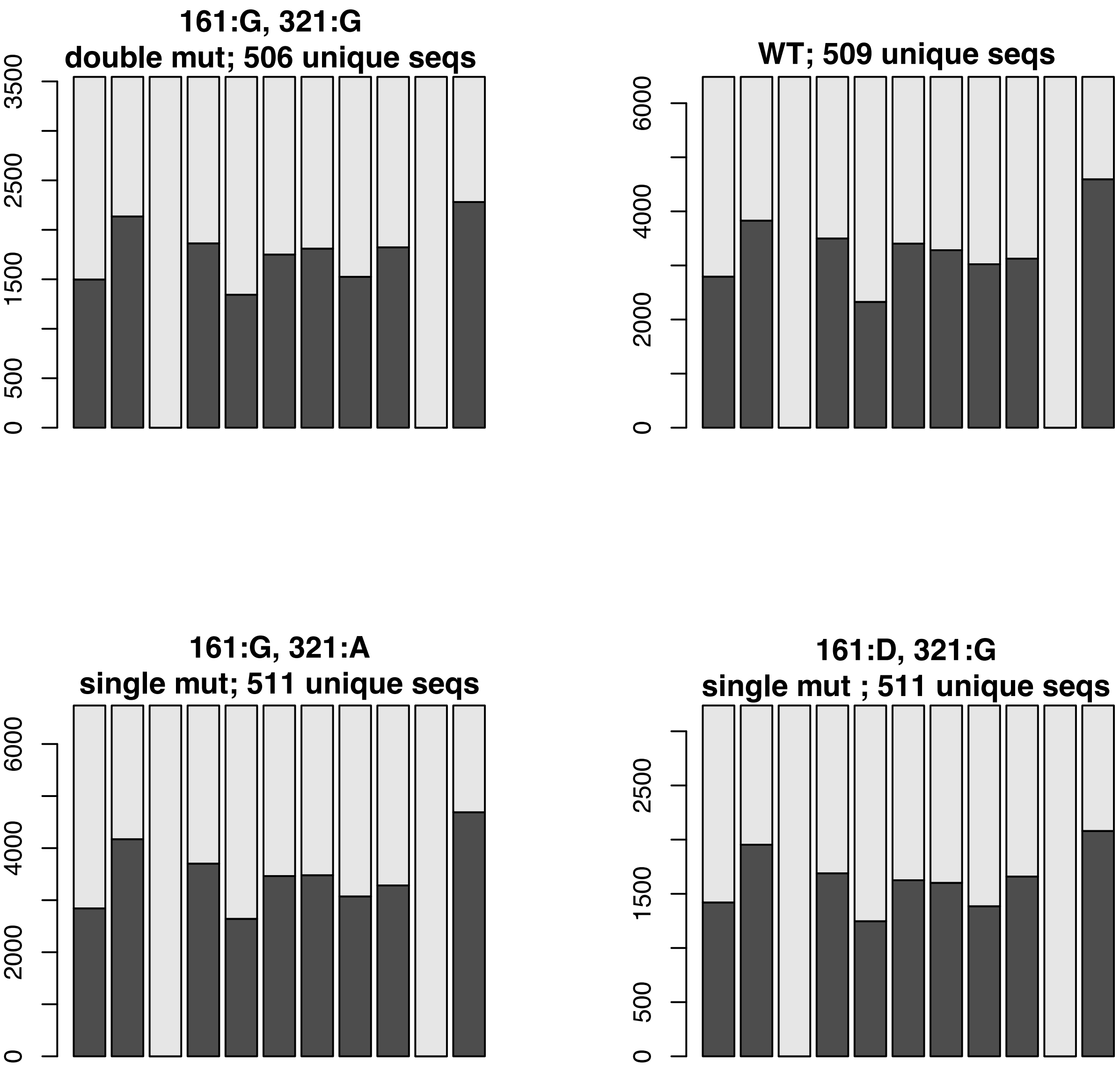
161:G, 321:G
double mut; 506 unique seqs
0
500
1500
2500
3500
WT; 509 unique seqs
0
2000
4000
6000
161:G, 321:A
single mut; 511 unique seqs
0
2000
4000
6000
161:D, 321:G
single mut ; 511 unique seqs
0
500
1500
2500

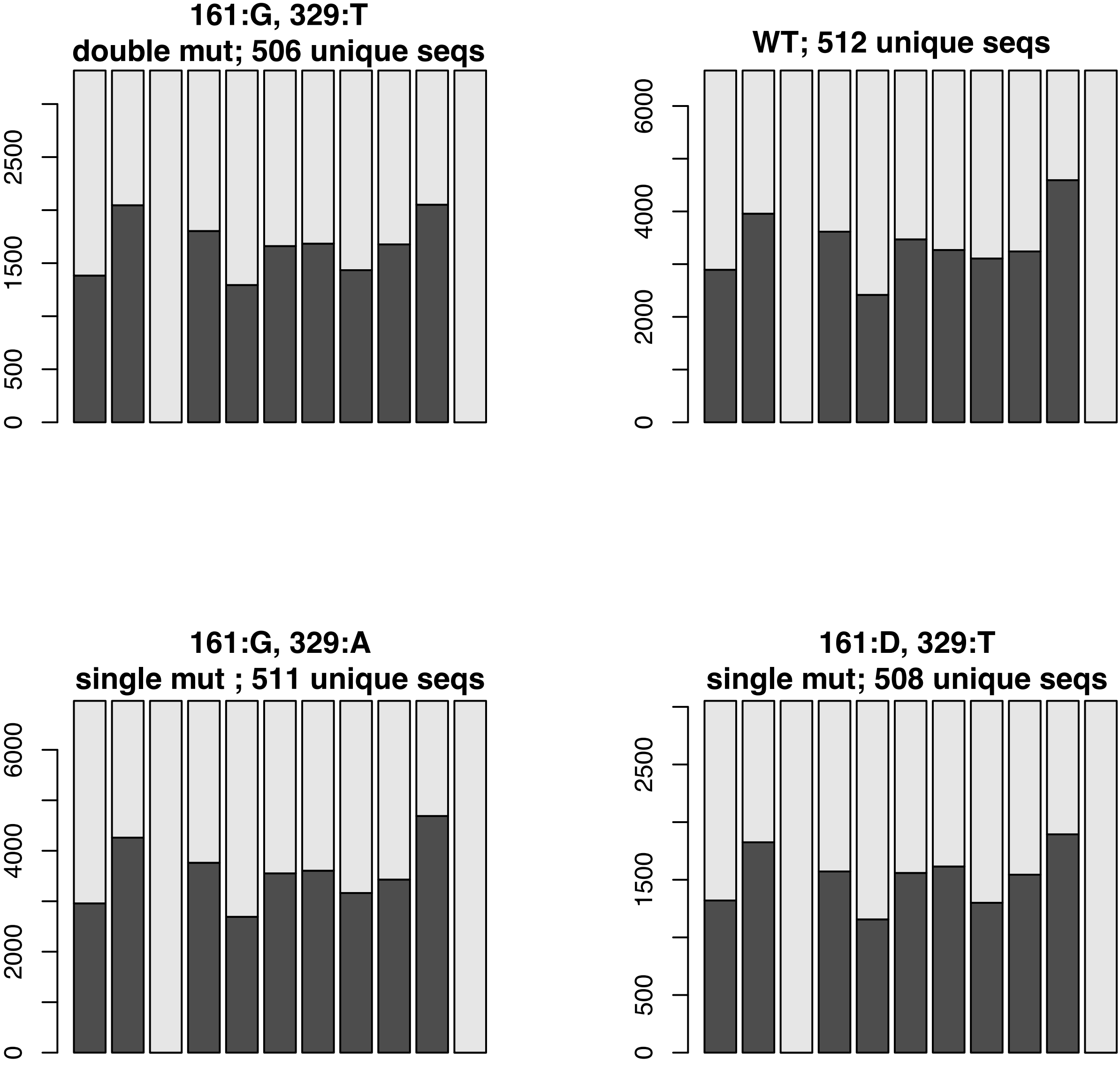
161:G, 329:T
double mut; 506 unique seqs
0
500
1500
2500
WT; 512 unique seqs
0
2000
4000
6000
161:G, 329:A
single mut ; 511 unique seqs
0
2000
4000
6000
161:D, 329:T
single mut; 508 unique seqs
0
500
1500
2500

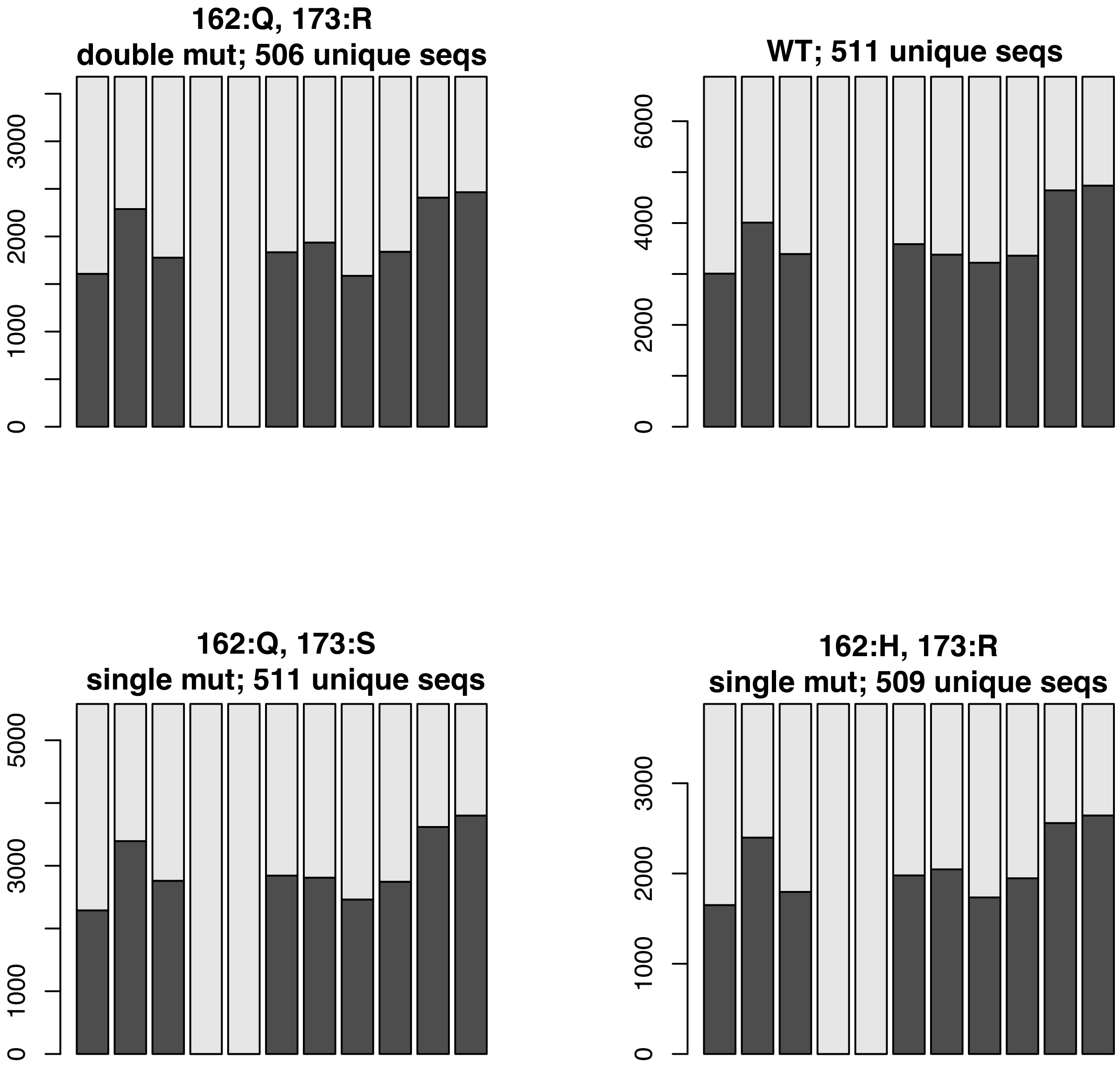

162:Q, 173:R
double mut; 506 unique seqs
0
1000
2000
3000
WT; 511 unique seqs
0
2000
4000
6000
162:Q, 173:S
single mut; 511 unique seqs
0
1000
3000
5000
162:H, 173:R
single mut; 509 unique seqs
0
1000
2000
3000

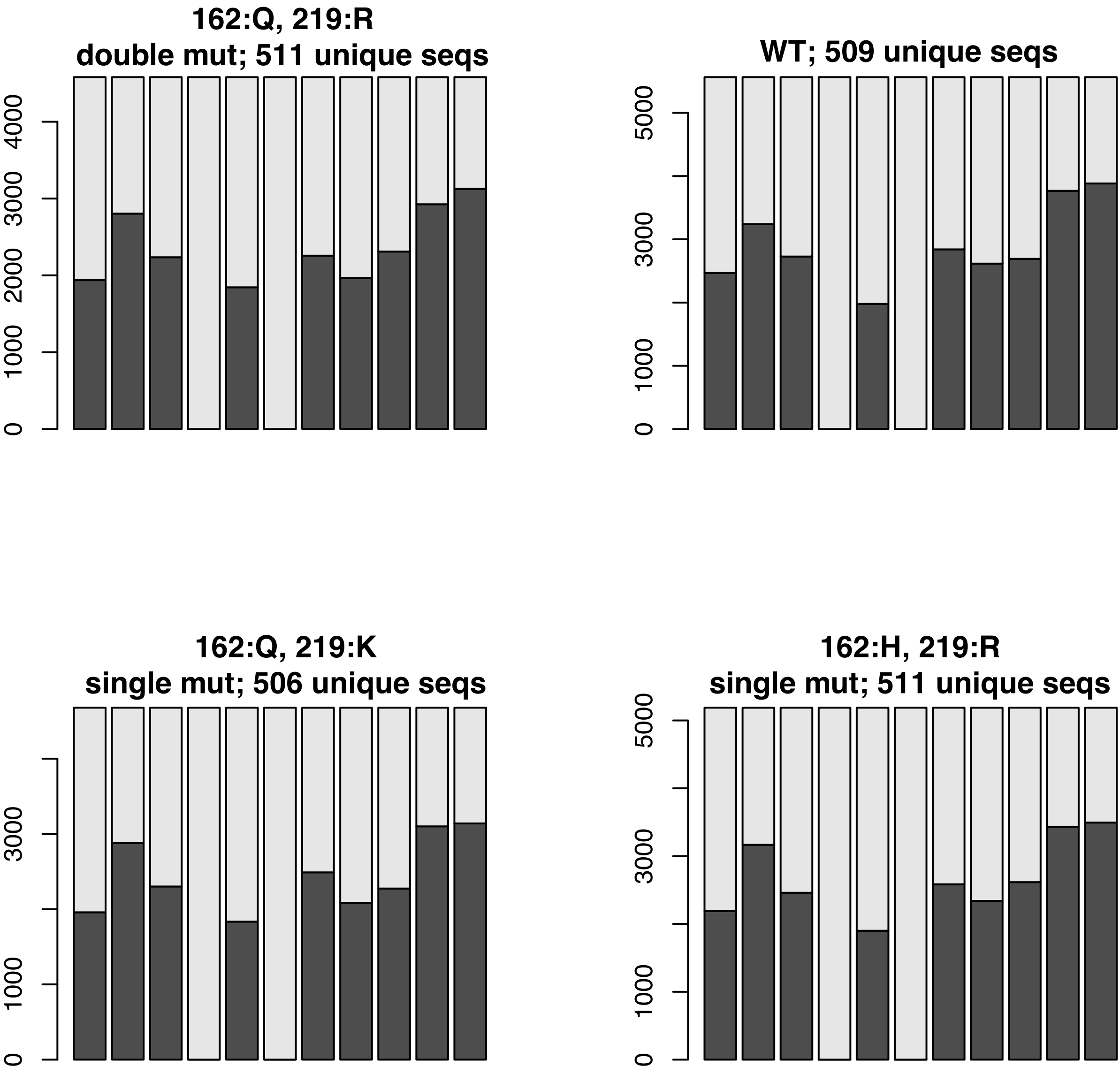
162:Q, 219:R
double mut; 511 unique seqs
0
1000
2000
3000
4000
WT; 509 unique seqs
0
1000
3000
5000
162:Q, 219:K
single mut; 506 unique seqs
0
1000
3000
162:H, 219:R
single mut; 511 unique seqs
0
1000
3000
5000

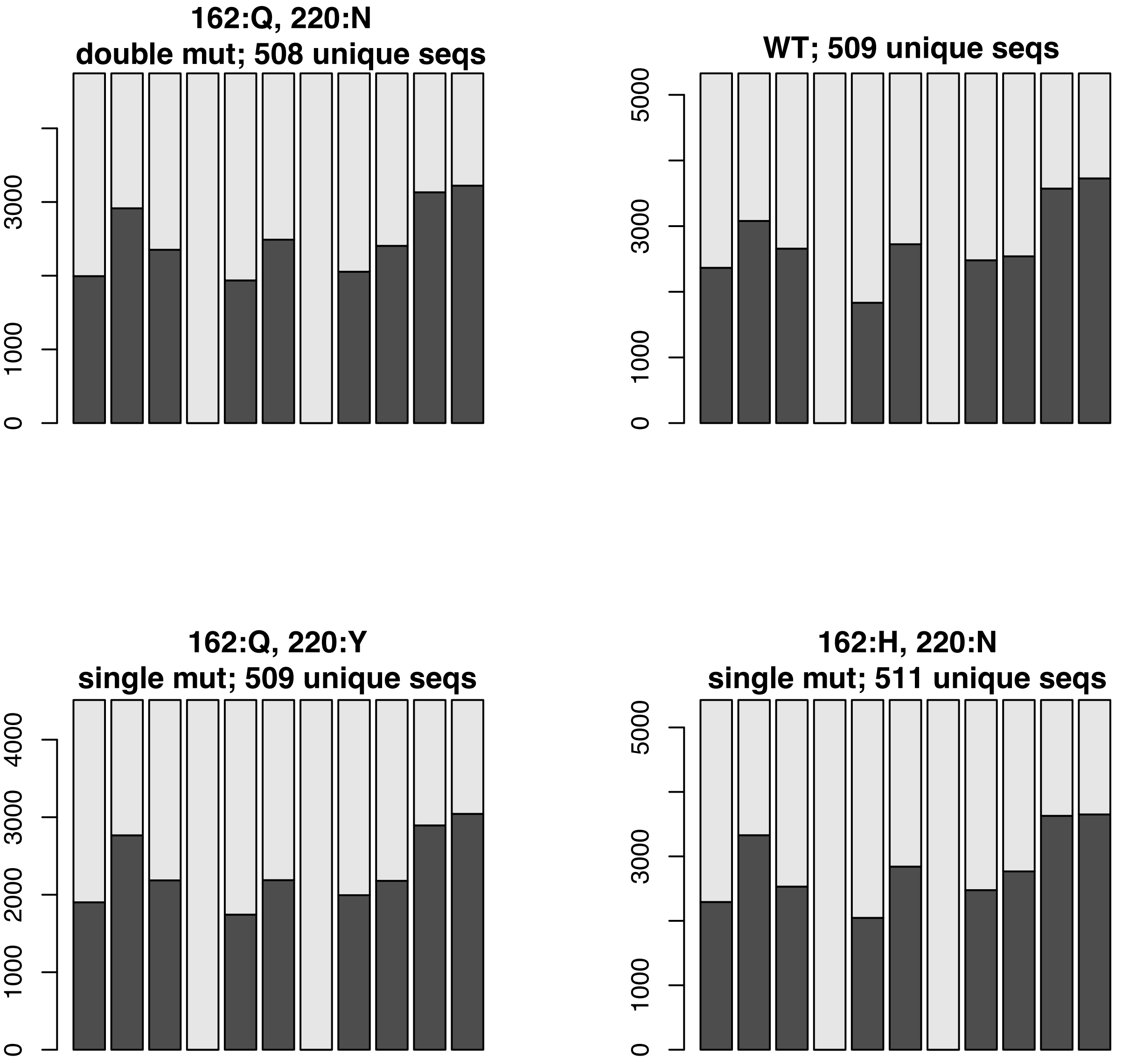

162:Q, 220:N
double mut; 508 unique seqs
0
1000
3000
WT; 509 unique seqs
0
1000
3000
5000
162:Q, 220:Y
single mut; 509 unique seqs
0
1000
2000
3000
4000
162:H, 220:N
single mut; 511 unique seqs
0
1000
3000
5000

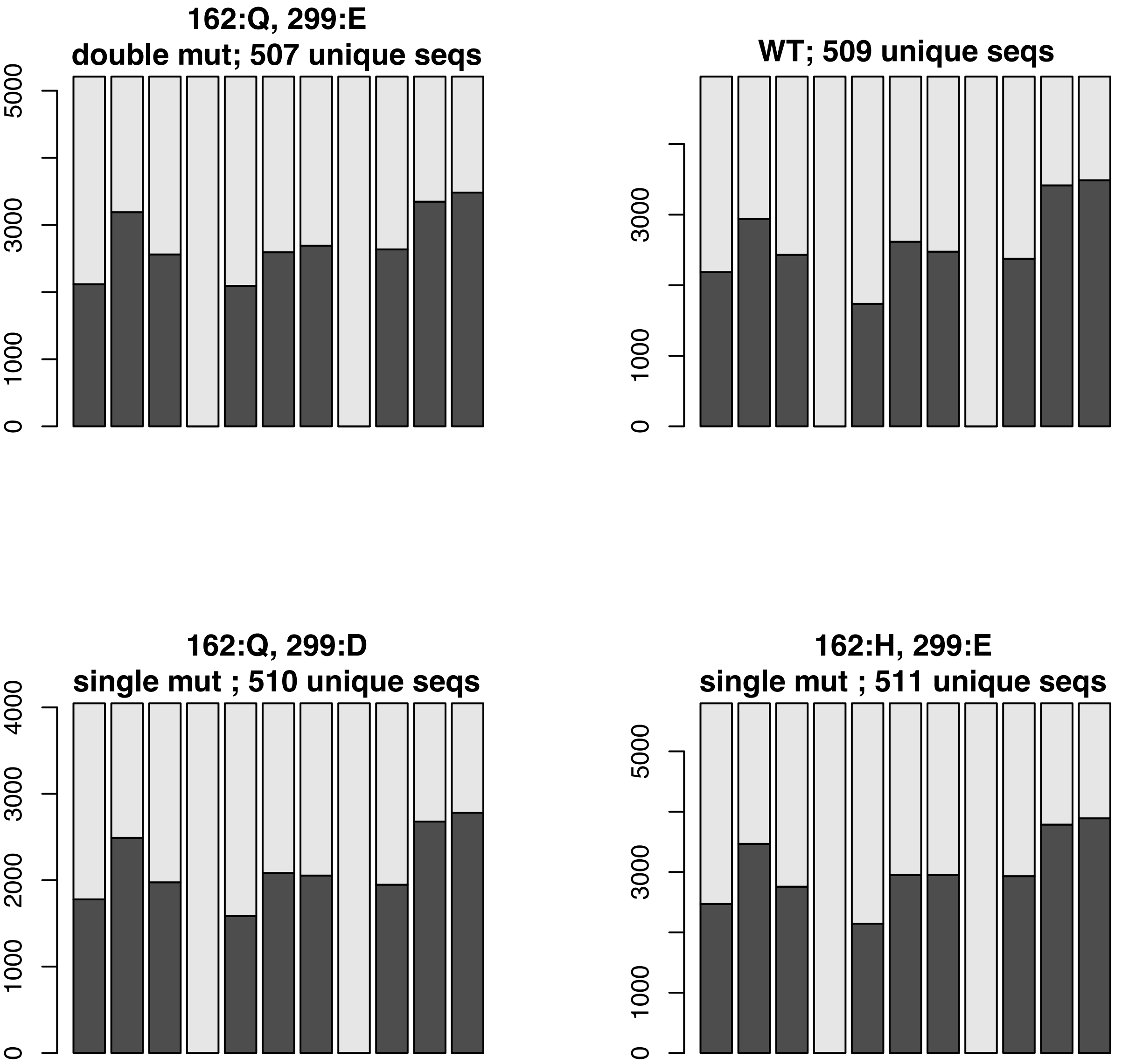
162:Q, 299:E
double mut; 507 unique seqs
0
1000
3000
5000
WT; 509 unique seqs
0
1000
3000
162:Q, 299:D
single mut ; 510 unique seqs
0
1000
2000
3000
4000
162:H, 299:E
single mut ; 511 unique seqs
0
1000
3000
5000

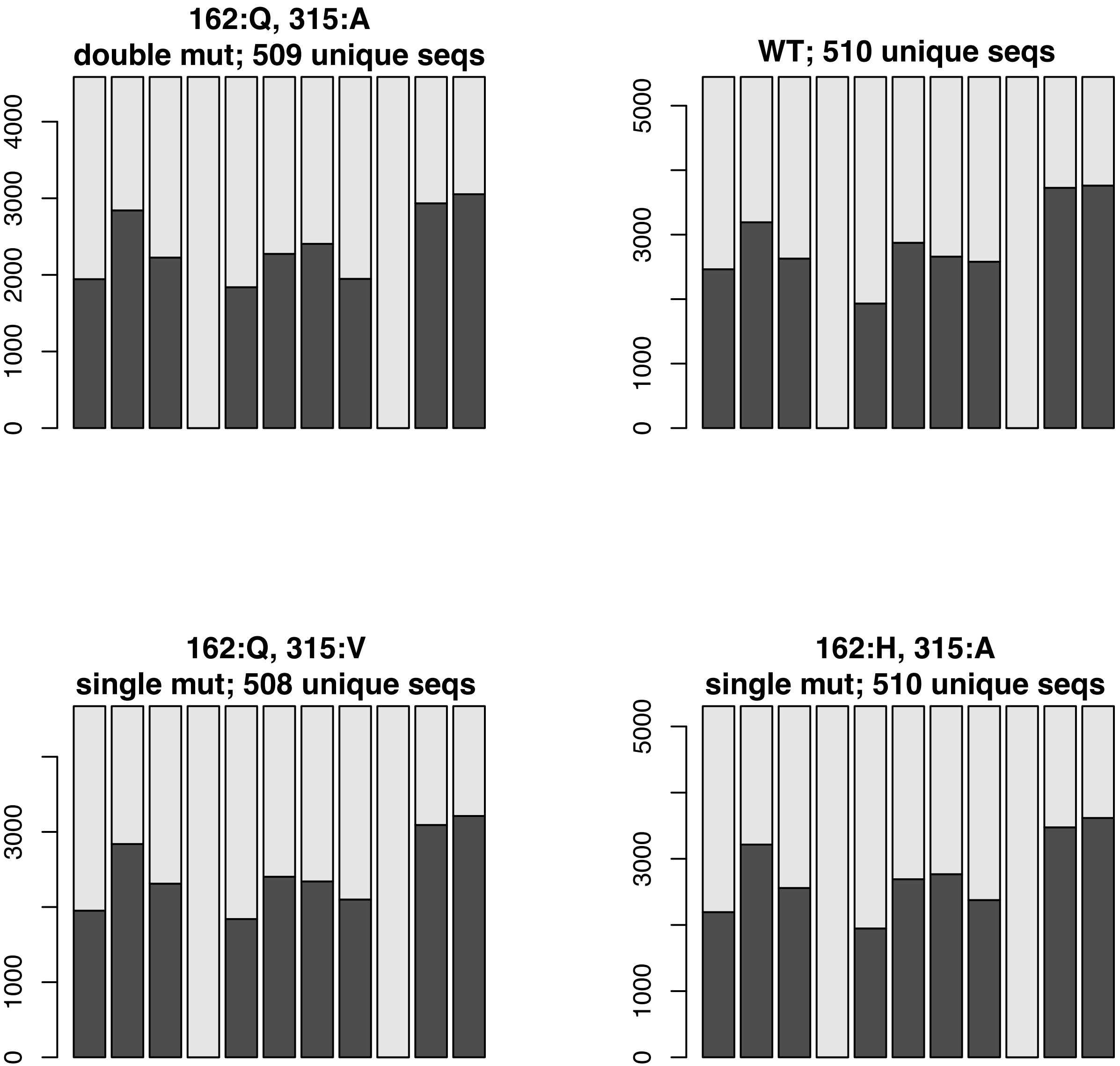

162:Q, 315:A
double mut; 509 unique seqs
0
1000
2000
3000
4000
WT; 510 unique seqs
0
1000
3000
5000
162:Q, 315:V
single mut; 508 unique seqs
0
1000
3000
162:H, 315:A
single mut; 510 unique seqs
0
1000
3000
5000

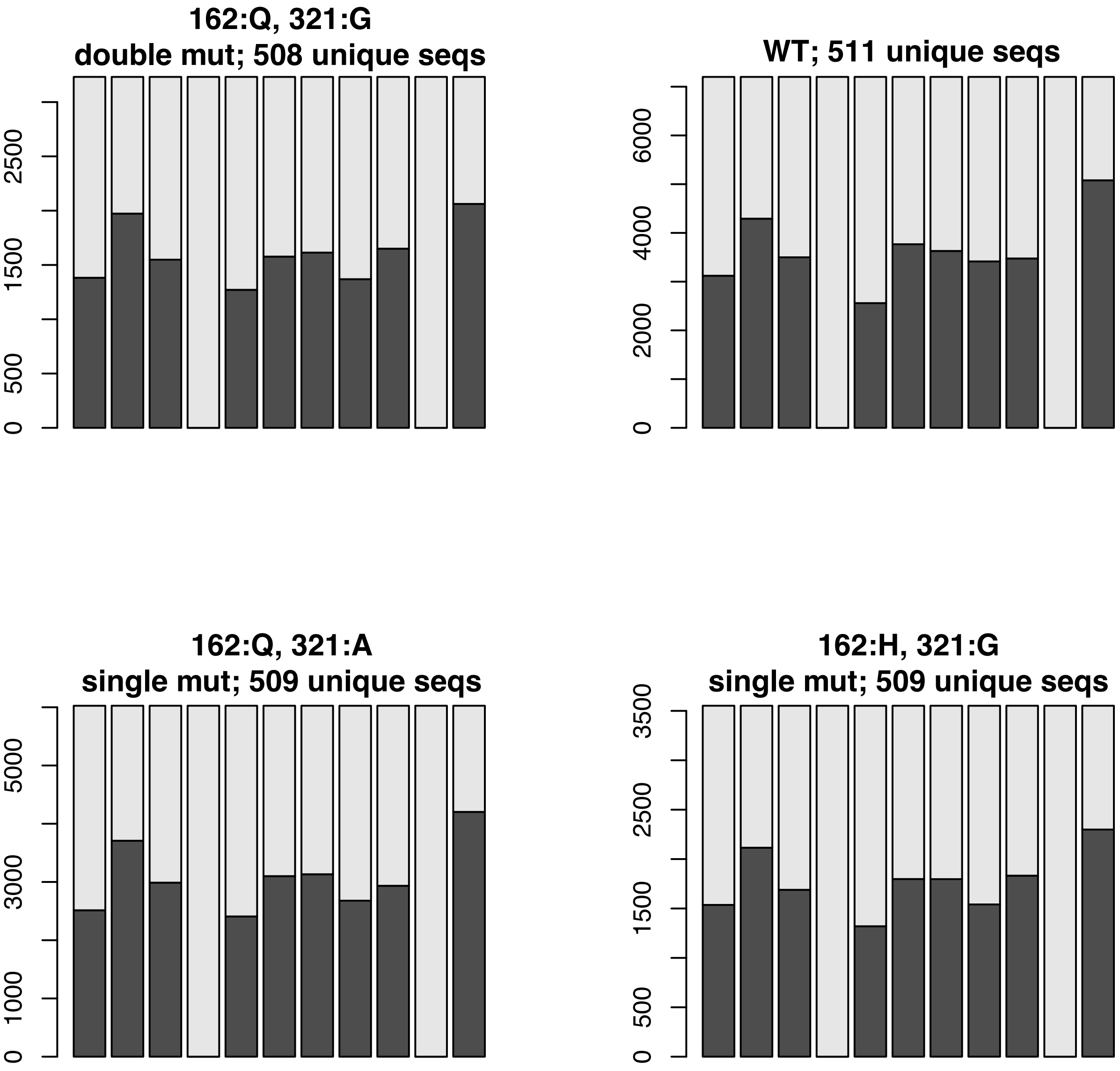
162:Q, 321:G
double mut; 508 unique seqs
0
500
1500
2500
WT; 511 unique seqs
0
2000
4000
6000
162:Q, 321:A
single mut; 509 unique seqs
0
1000
3000
5000
162:H, 321:G
single mut; 509 unique seqs
0
500
1500
2500
3500

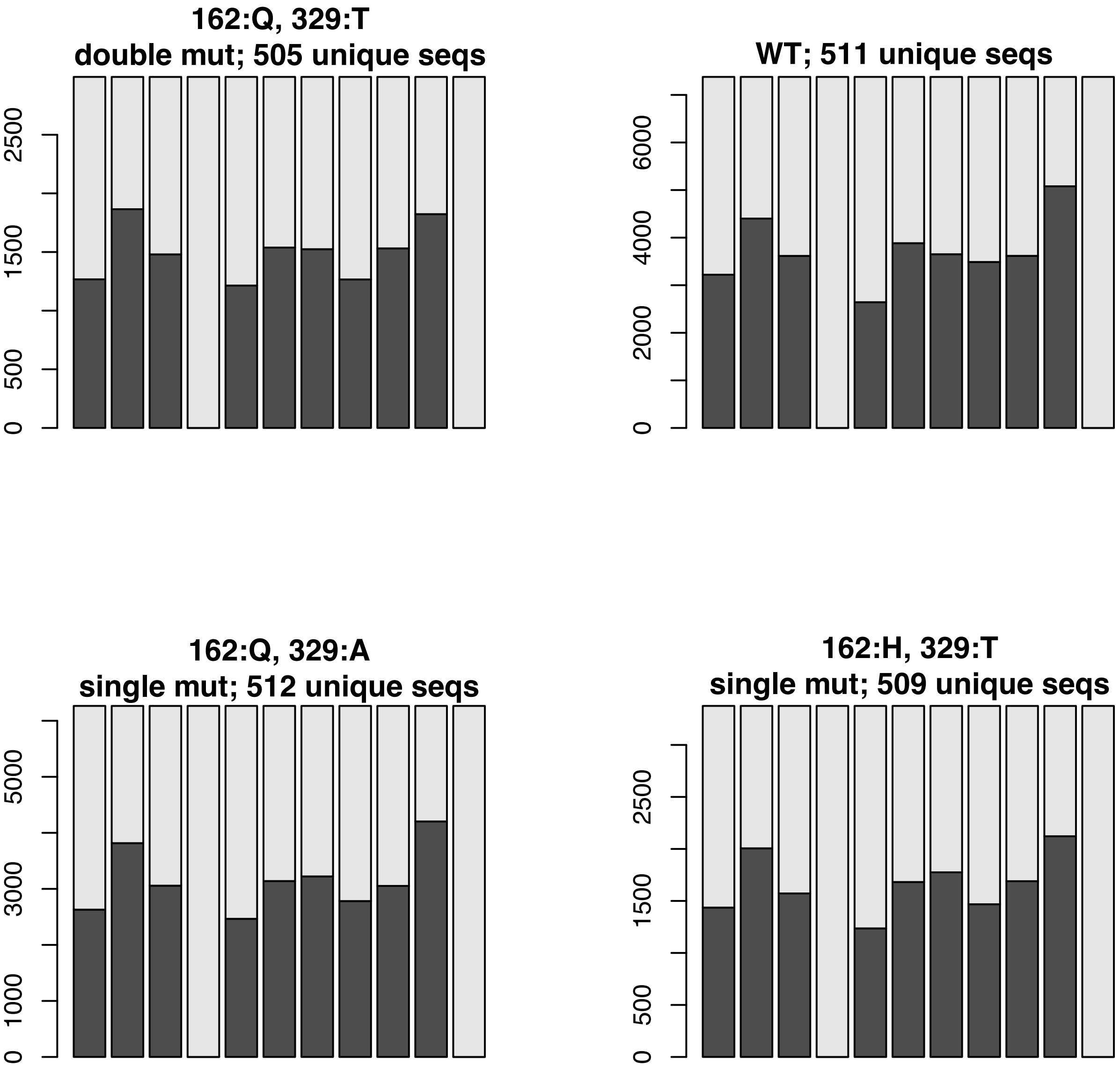
162:Q, 329:T
double mut; 505 unique seqs
0
500
1500
2500
WT; 511 unique seqs
0
2000
4000
6000
162:Q, 329:A
single mut; 512 unique seqs
0
1000
3000
5000
162:H, 329:T
single mut; 509 unique seqs
0
500
1500
2500

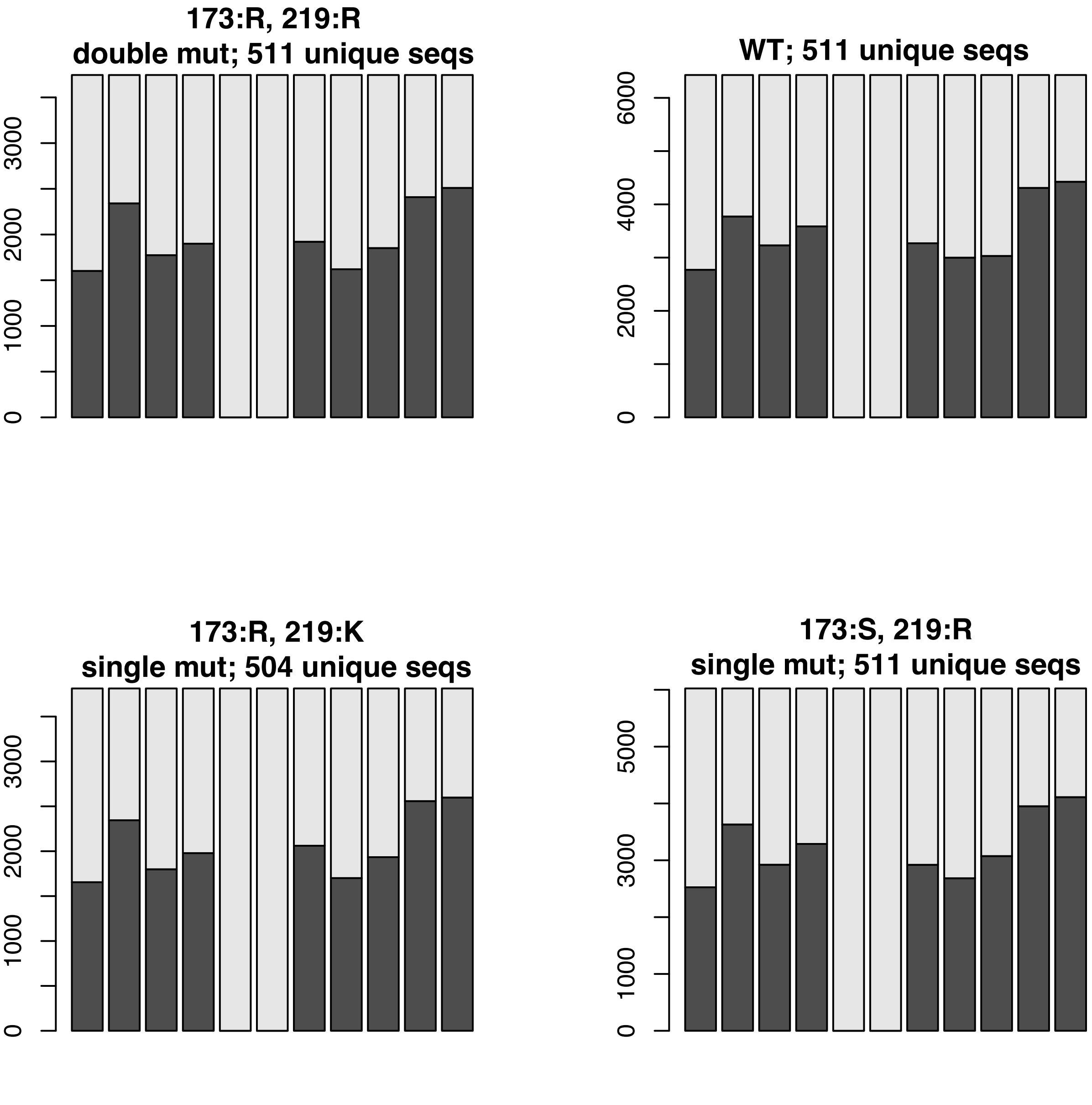

173:R, 219:R
double mut; 511 unique seqs
0
1000
2000
3000
WT; 511 unique seqs
0
2000
4000
6000
173:R, 219:K
single mut; 504 unique seqs
0
1000
2000
3000
173:S, 219:R
single mut; 511 unique seqs
0
1000
3000
5000

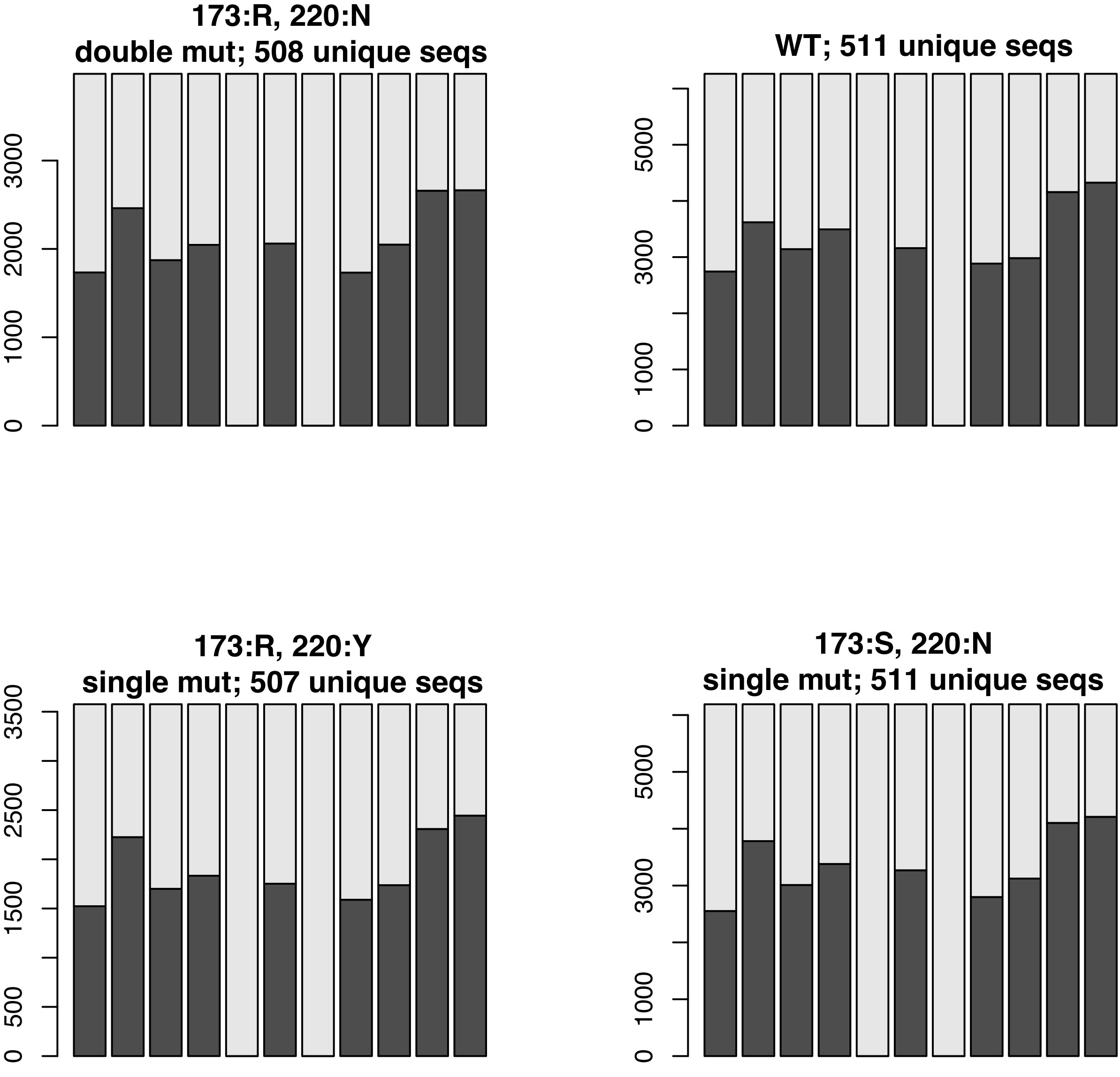
173:R, 220:N
double mut; 508 unique seqs
0
1000
2000
3000
WT; 511 unique seqs
0
1000
3000
5000
173:R, 220:Y
single mut; 507 unique seqs
0
500
1500
2500
3500
173:S, 220:N
single mut; 511 unique seqs
0
1000
3000
5000

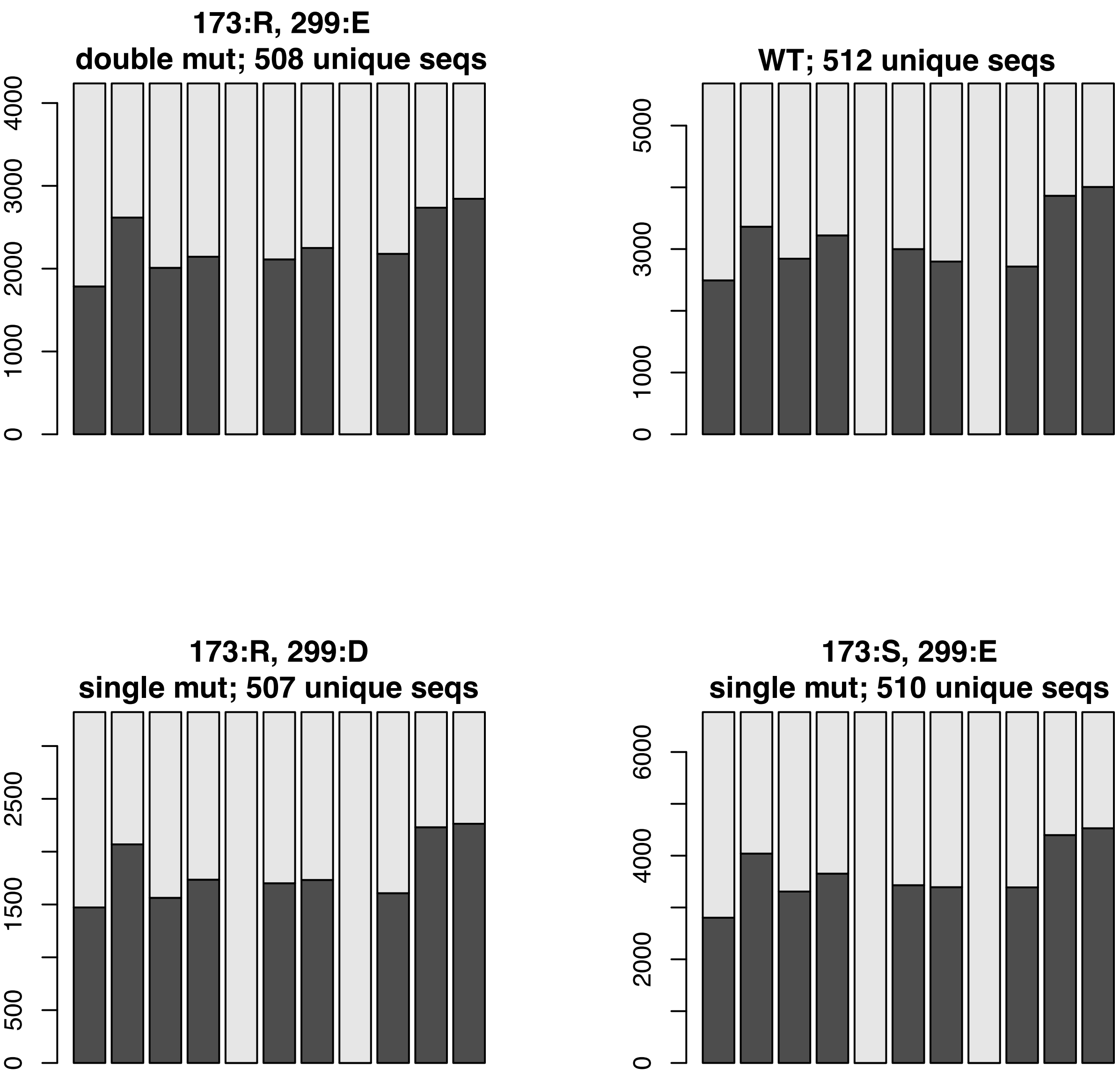
173:R, 299:E
double mut; 508 unique seqs
0
1000
2000
3000
4000
WT; 512 unique seqs
0
1000
3000
5000
173:R, 299:D
single mut; 507 unique seqs
0
500
1500
2500
173:S, 299:E
single mut; 510 unique seqs
0
2000
4000
6000

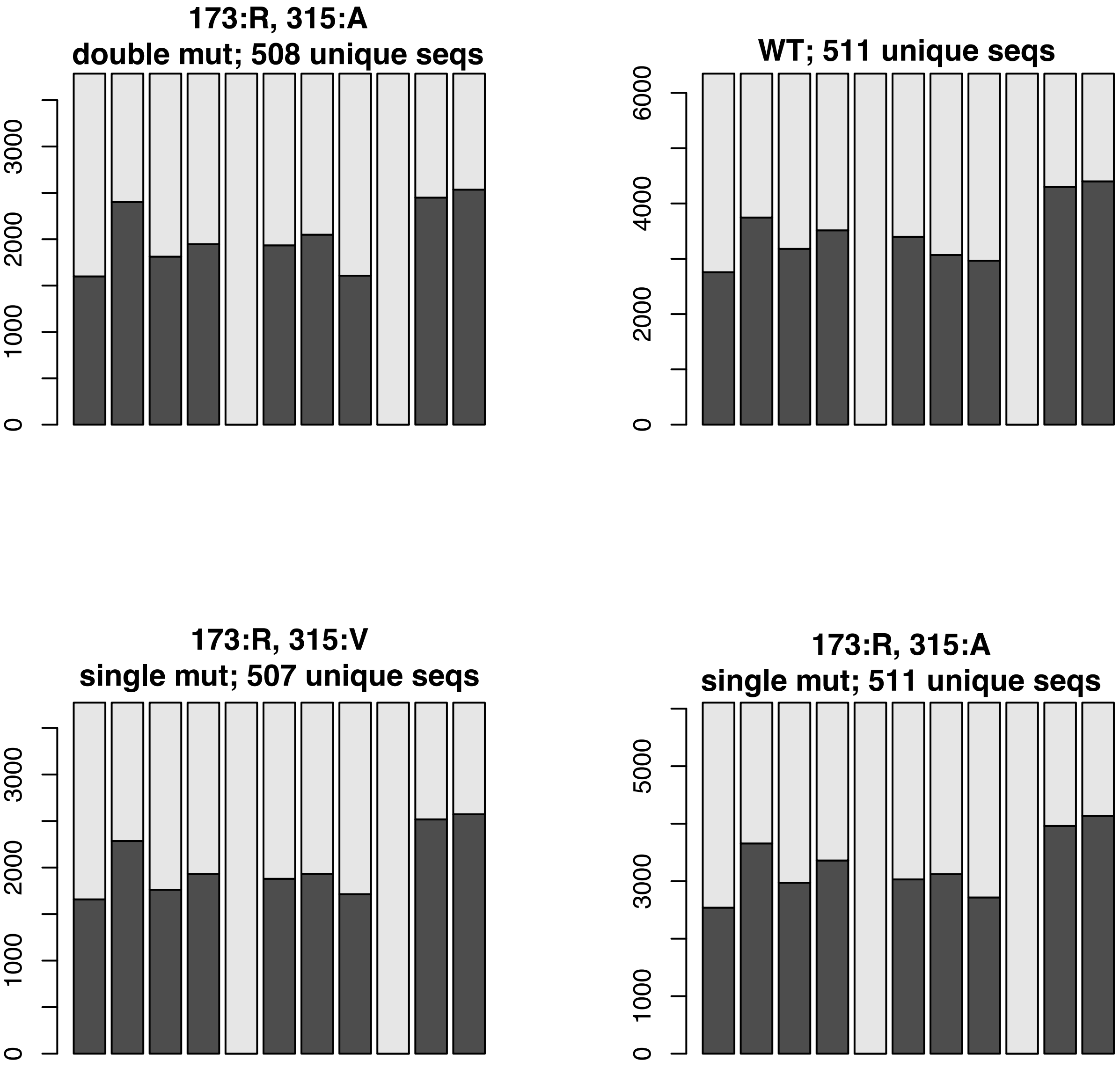

173:R, 315:A
double mut; 508 unique seqs
0
1000
2000
3000
WT; 511 unique seqs
0
2000
4000
6000
173:R, 315:V
single mut; 507 unique seqs
0
1000
2000
3000
173:R, 315:A
single mut; 511 unique seqs
0
1000
3000
5000

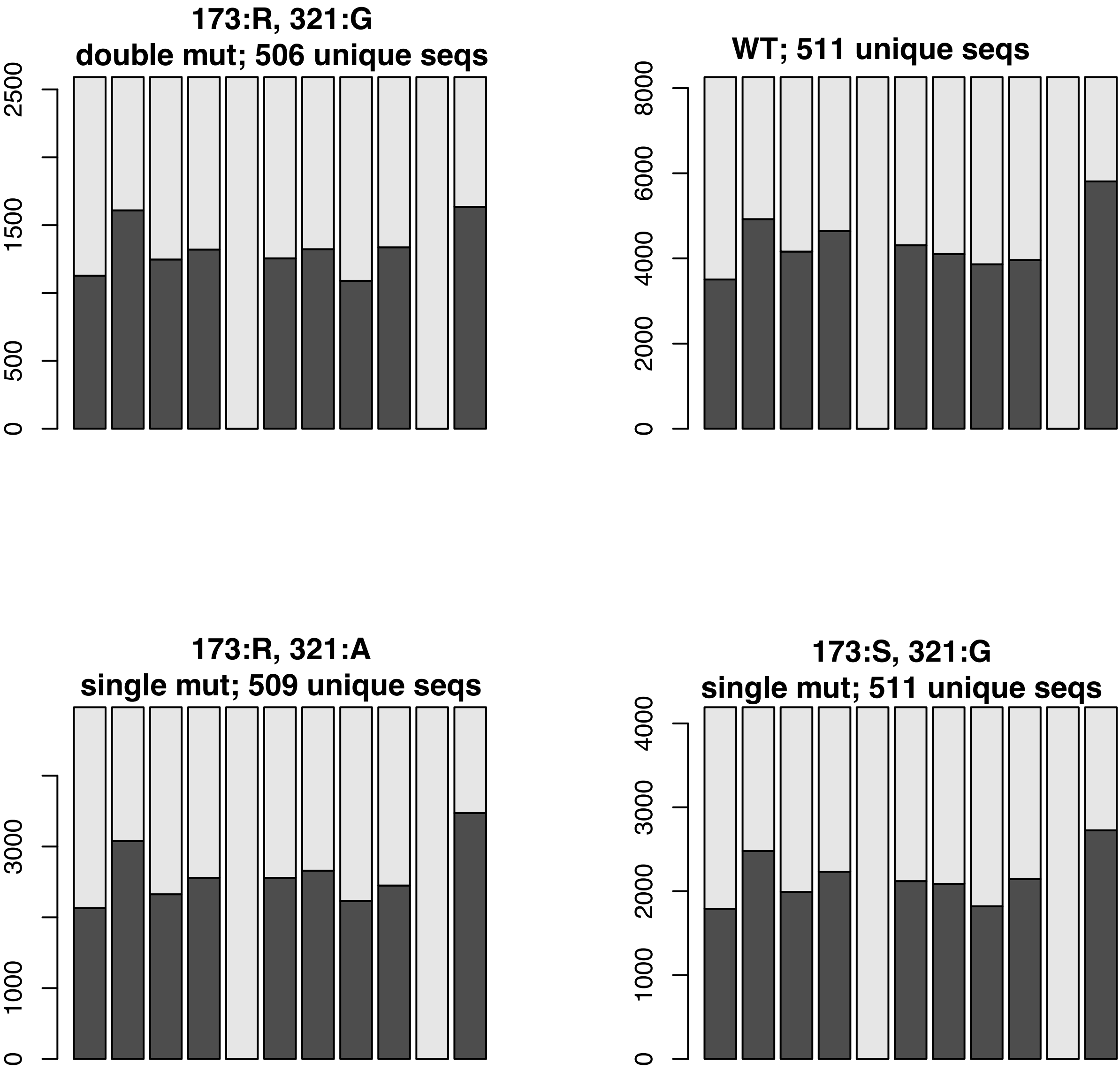
173:R, 321:G
double mut; 506 unique seqs
0
500
1500
2500
WT; 511 unique seqs
0
2000
4000
6000
8000
173:R, 321:A
single mut; 509 unique seqs
0
1000
3000
173:S, 321:G
single mut; 511 unique seqs
0
1000
2000
3000
4000

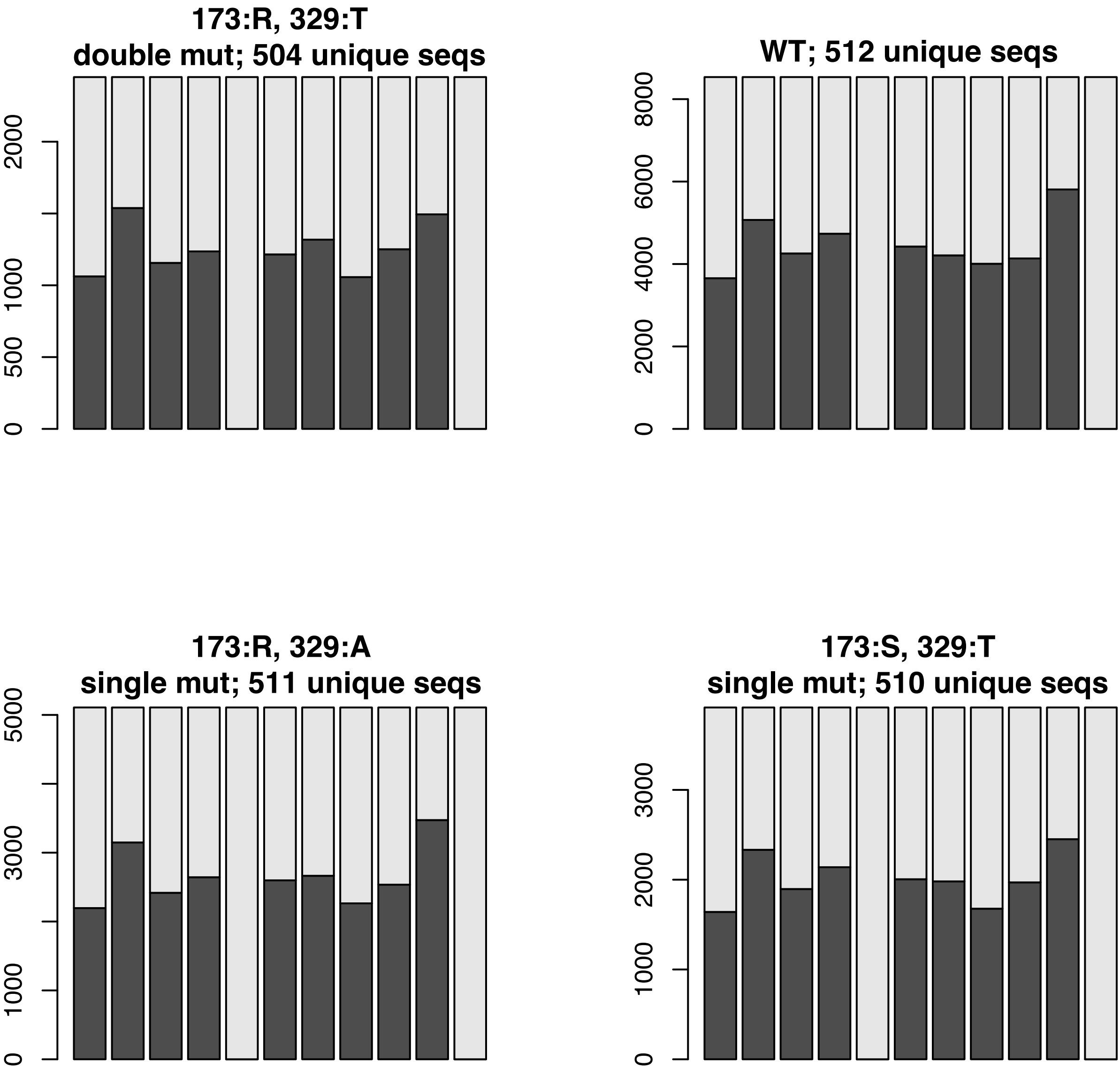

173:R, 329:T
double mut; 504 unique seqs
0
500
1000
2000
WT; 512 unique seqs
0
2000
4000
6000
8000
173:R, 329:A
single mut; 511 unique seqs
0
1000
3000
5000
173:S, 329:T
single mut; 510 unique seqs
0
1000
2000
3000

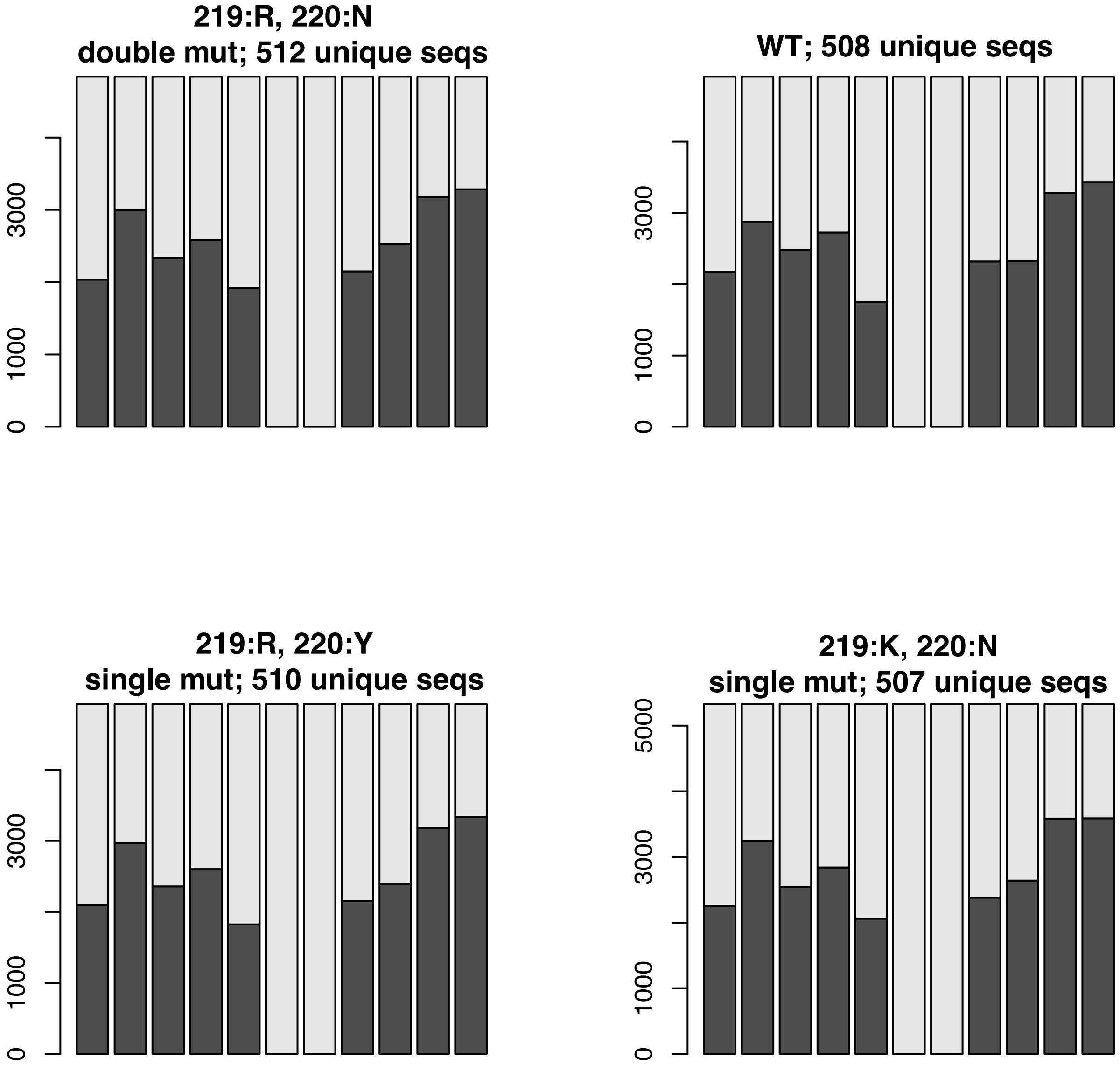
219:R, 220:N
double mut; 512 unique seqs
0
1000
3000
WT; 508 unique seqs
0
1000
3000
219:R, 220:Y
single mut; 510 unique seqs
0
1000
3000
219:K, 220:N
single mut; 507 unique seqs
0
1000
3000
5000

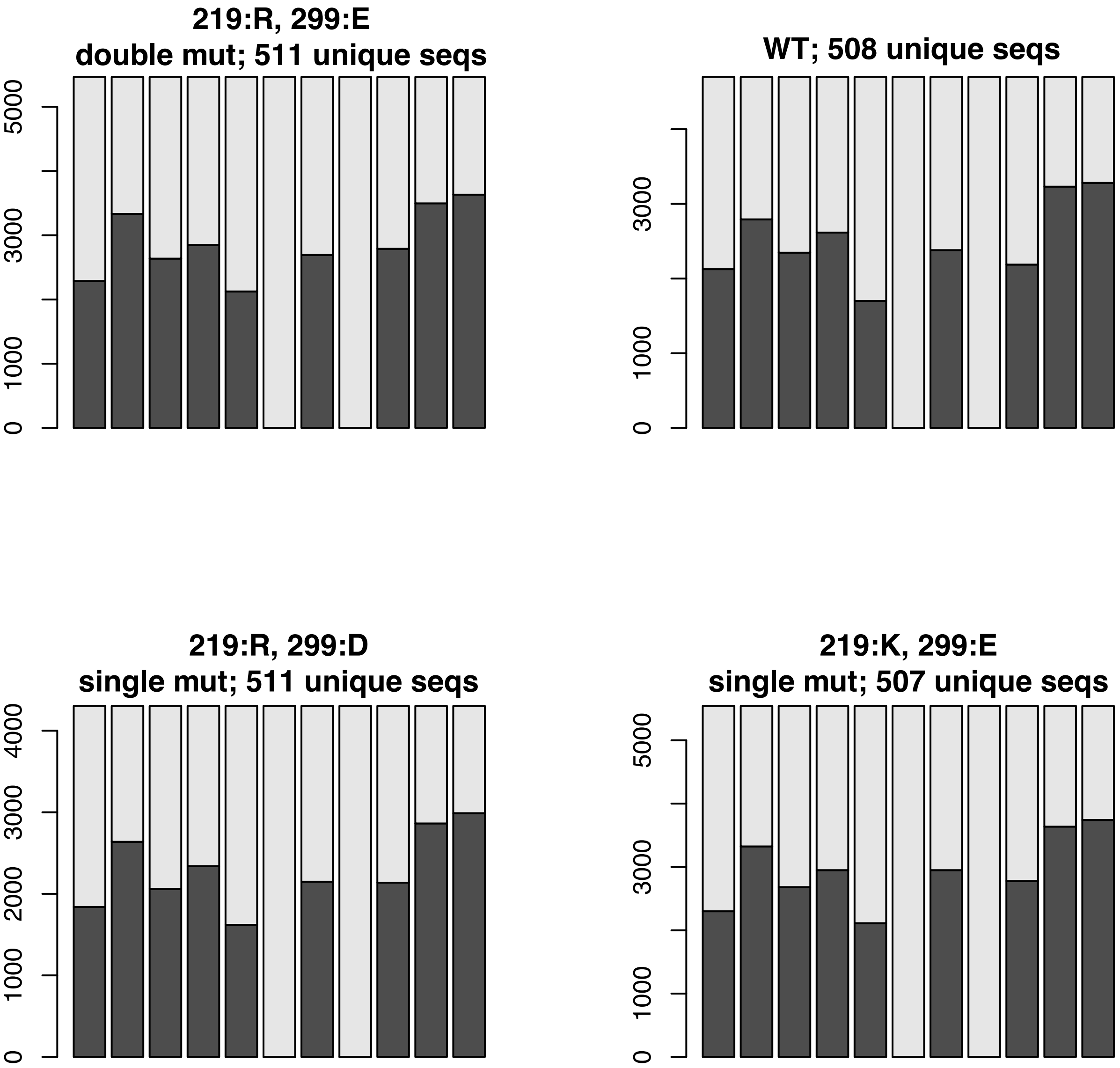
219:R, 299:E
double mut; 511 unique seqs
0
1000
3000
5000
WT; 508 unique seqs
0
1000
3000
219:R, 299:D
single mut; 511 unique seqs
0
1000
2000
3000
4000
219:K, 299:E
single mut; 507 unique seqs
0
1000
3000
5000

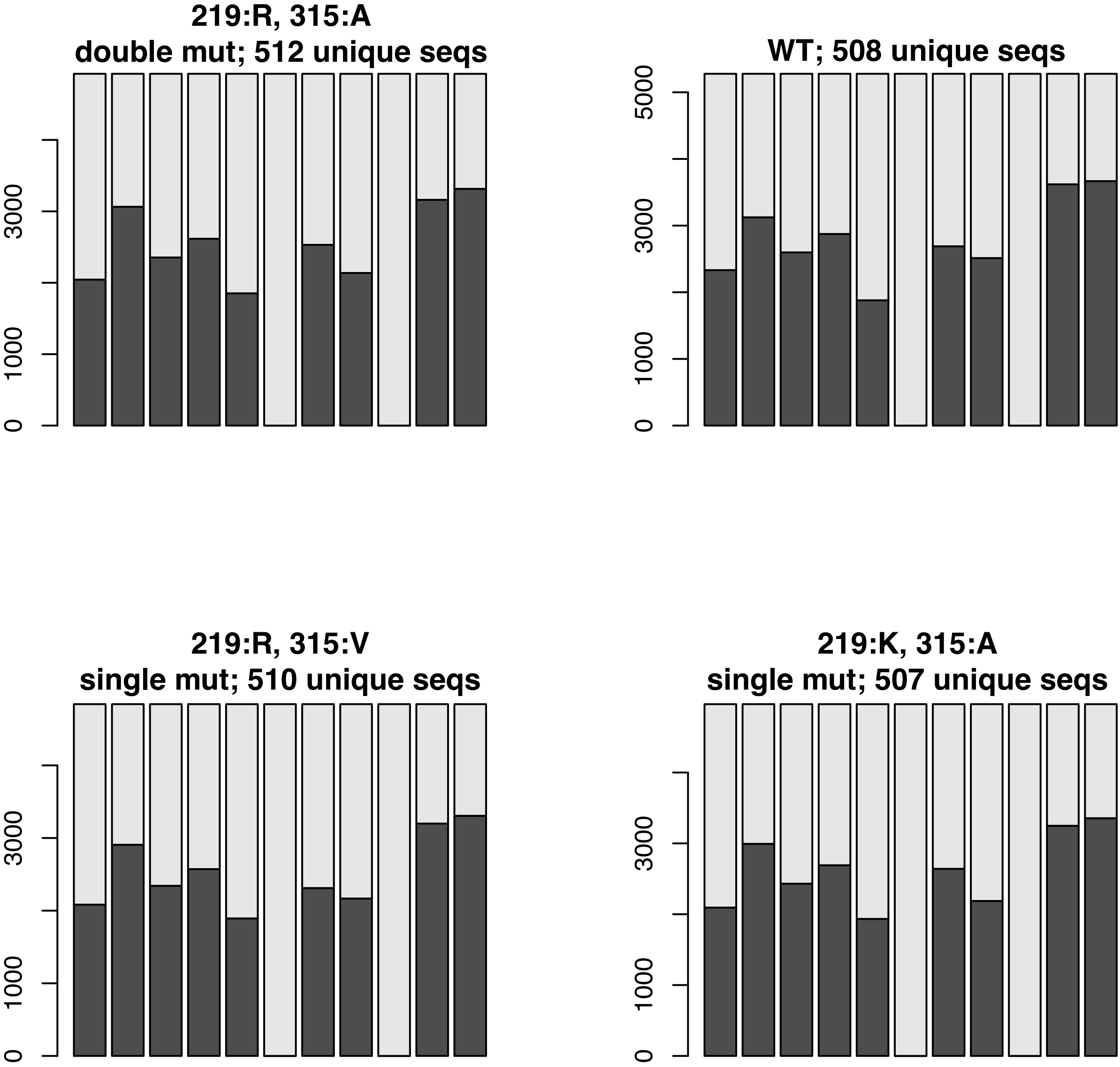
219:R, 315:A
double mut; 512 unique seqs
WT; 508 unique seqs
219:R, 315:V
single mut; 510 unique seqs
219:K, 315:A
single mut; 507 unique seqs
0
1000
3000
5000

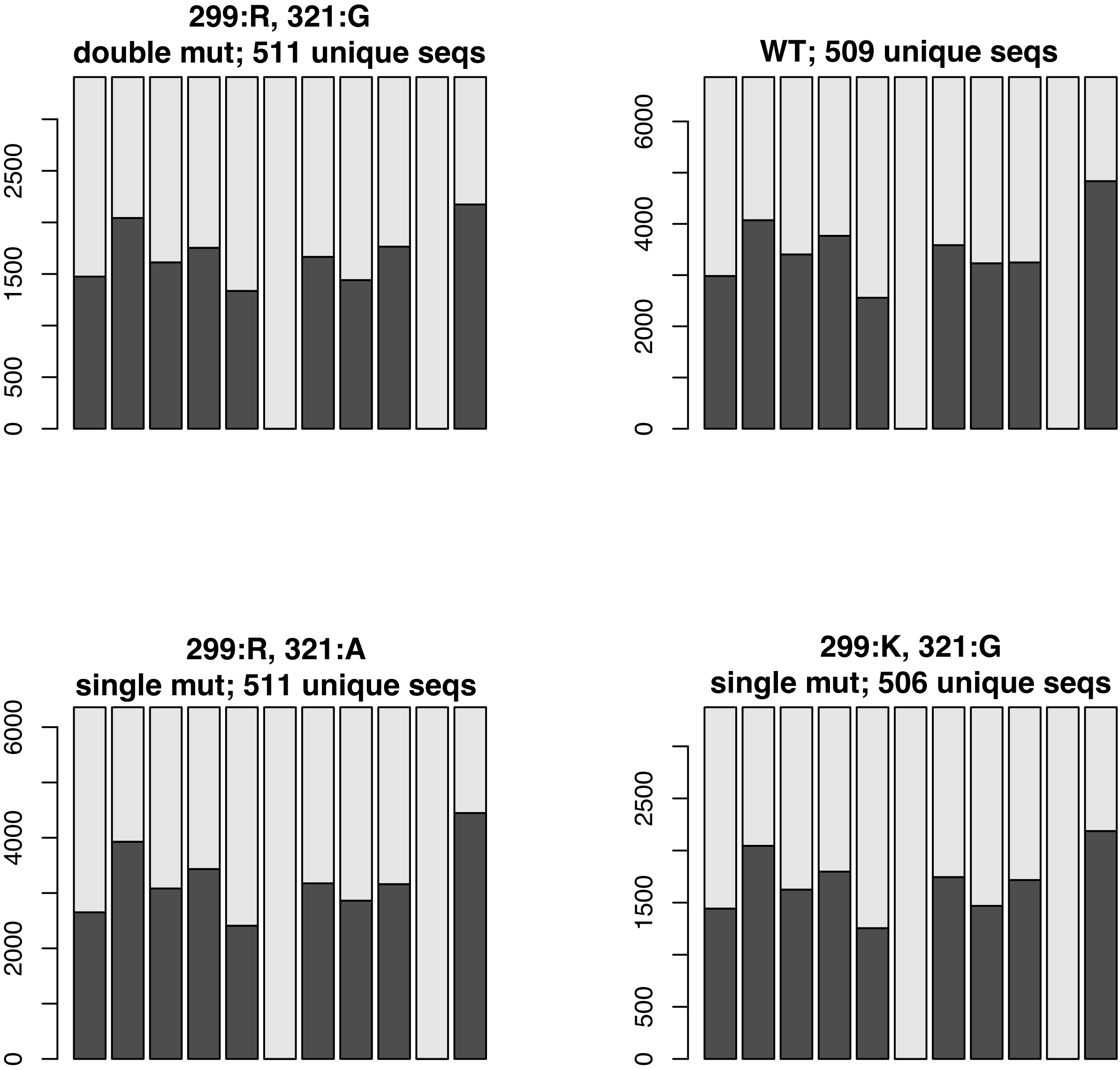
299:R, 321:G
double mut; 511 unique seqs
0
500
1500
2500
WT; 509 unique seqs
0
2000
4000
6000
299:R, 321:A
single mut; 511 unique seqs
0
2000
4000
6000
299:K, 321:G
single mut; 506 unique seqs
0
500
1500
2500

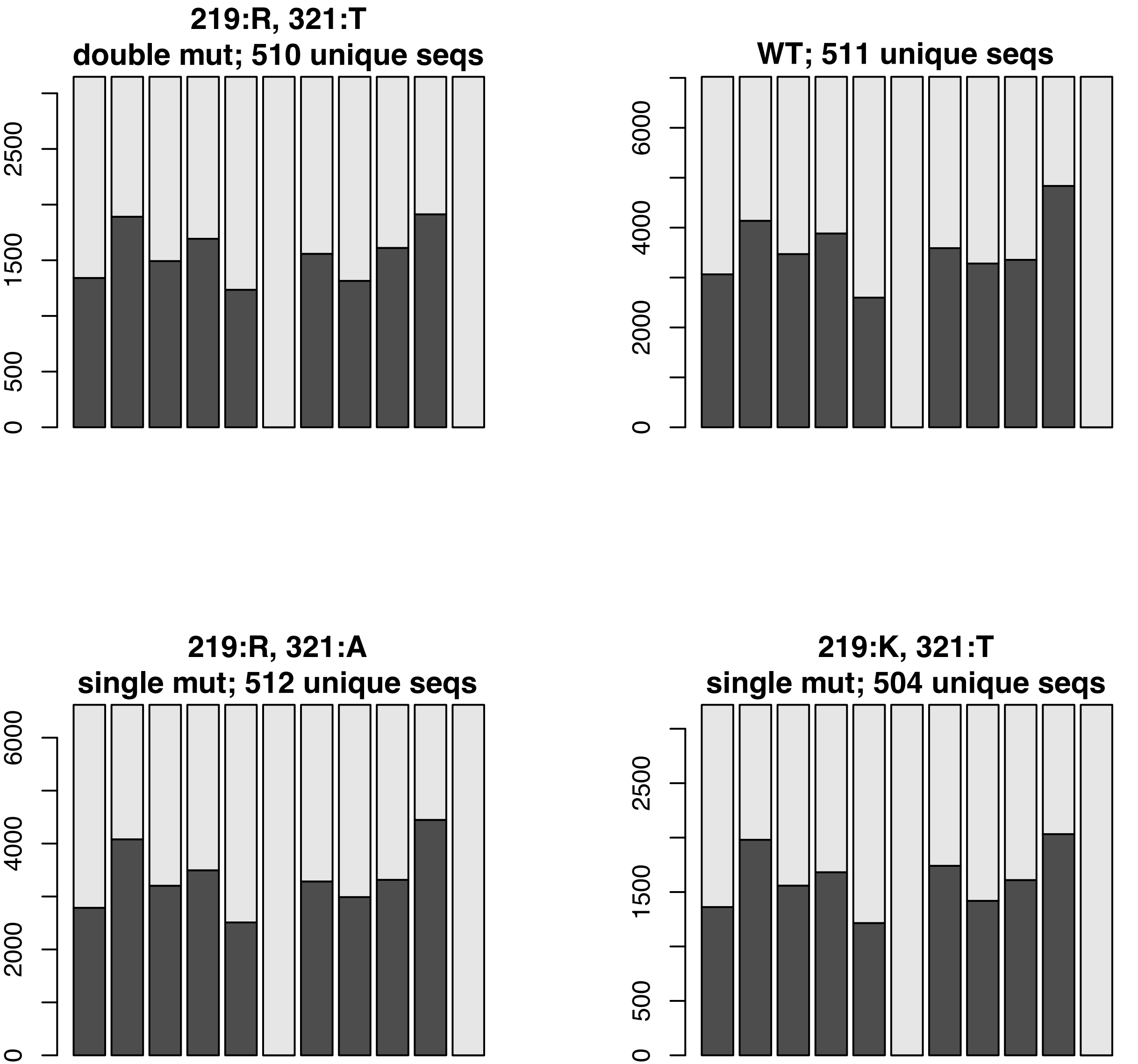
219:R, 321:T
double mut; 510 unique seqs
0
500
1500
2500
WT; 511 unique seqs
0
2000
4000
6000
219:R, 321:A
single mut; 512 unique seqs
0
2000
4000
6000
219:K, 321:T
single mut; 504 unique seqs
0
500
1500
2500

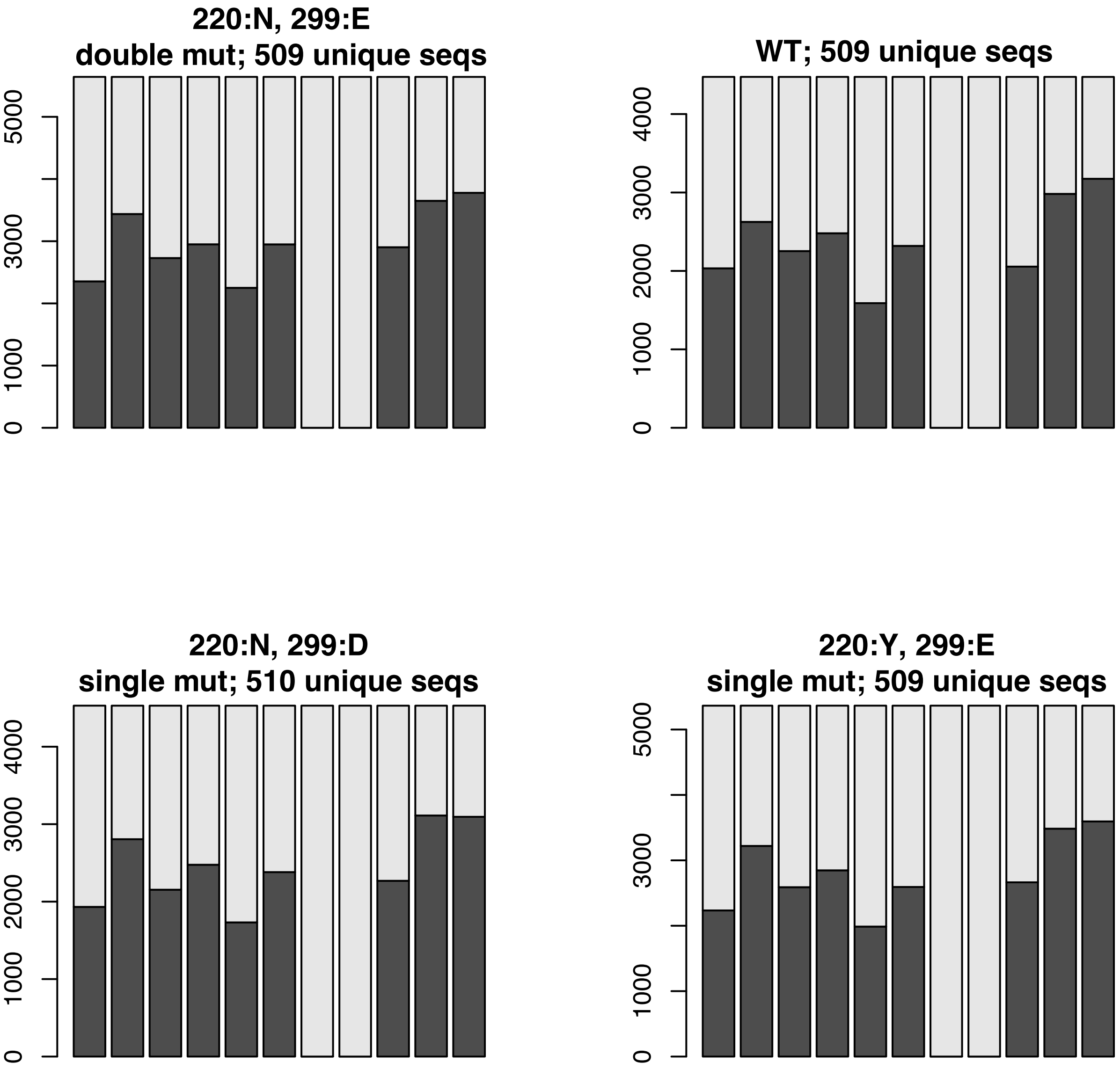
220:N, 299:E
double mut; 509 unique seqs
0
1000
3000
5000
WT; 509 unique seqs
0
1000
2000
3000
4000
220:N, 299:D
single mut; 510 unique seqs
0
1000
2000
3000
4000
220:Y, 299:E
single mut; 509 unique seqs
0
1000
3000
5000

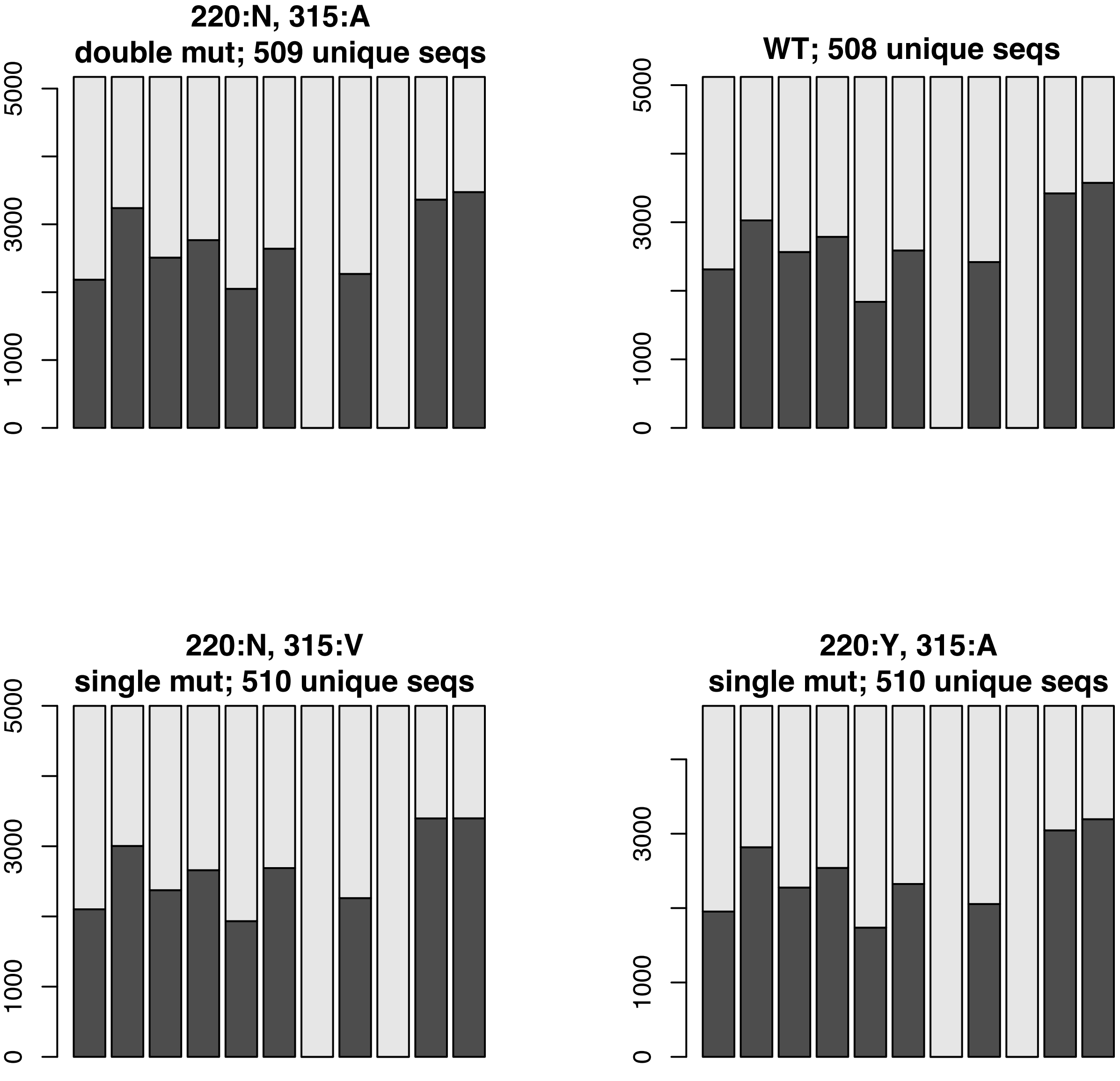

220:N, 315:A
double mut; 509 unique seqs
0
1000
3000
5000
WT; 508 unique seqs
0
1000
3000
5000
220:N, 315:V
single mut; 510 unique seqs
0
1000
3000
5000
220:Y, 315:A
single mut; 510 unique seqs
0
1000
3000

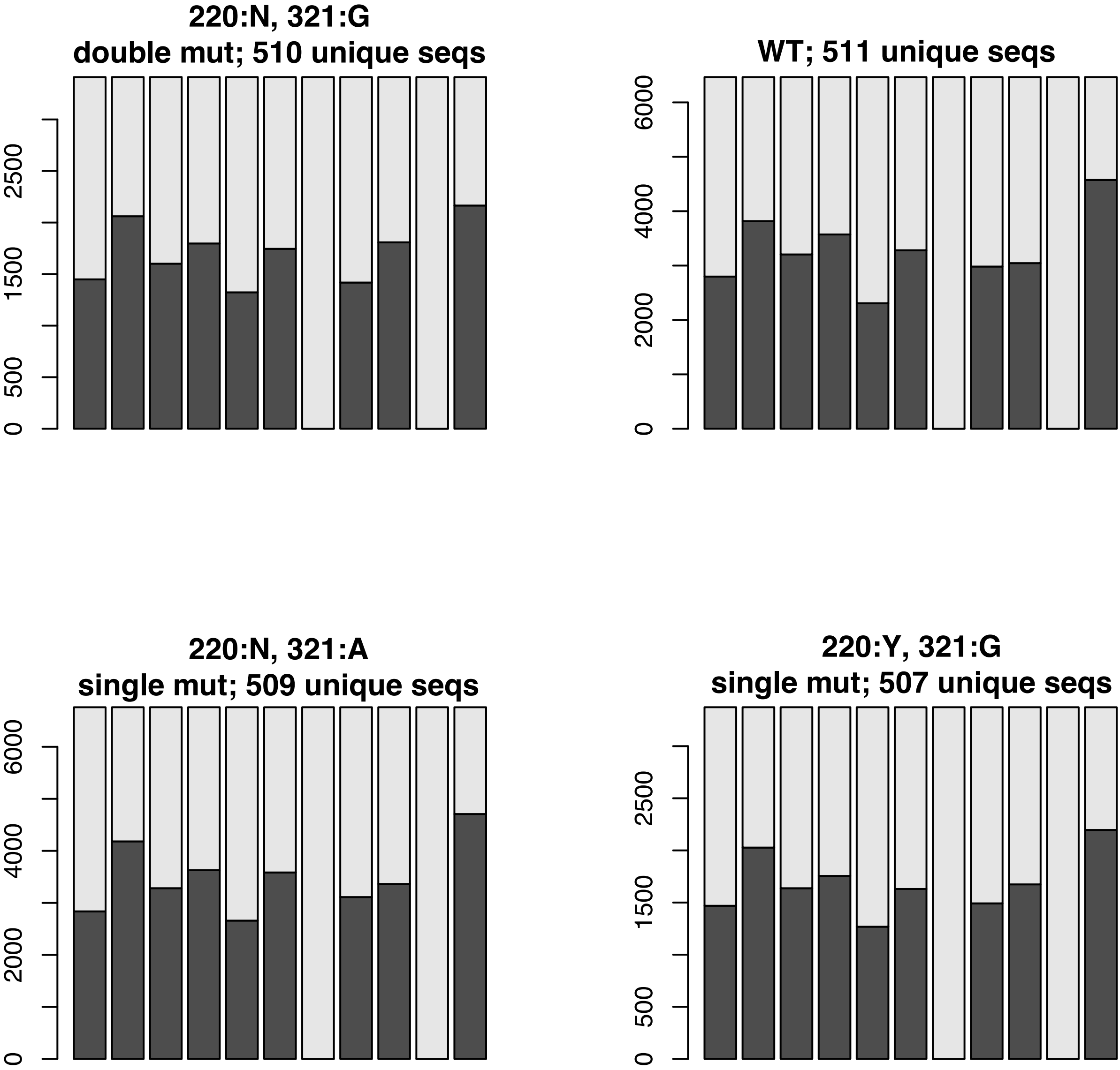
220:N, 321:G
double mut; 510 unique seqs
0
500
1500
2500
WT; 511 unique seqs
0
2000
4000
6000
220:N, 321:A
single mut; 509 unique seqs
0
2000
4000
6000
220:Y, 321:G
single mut; 507 unique seqs
0
500
1500
2500

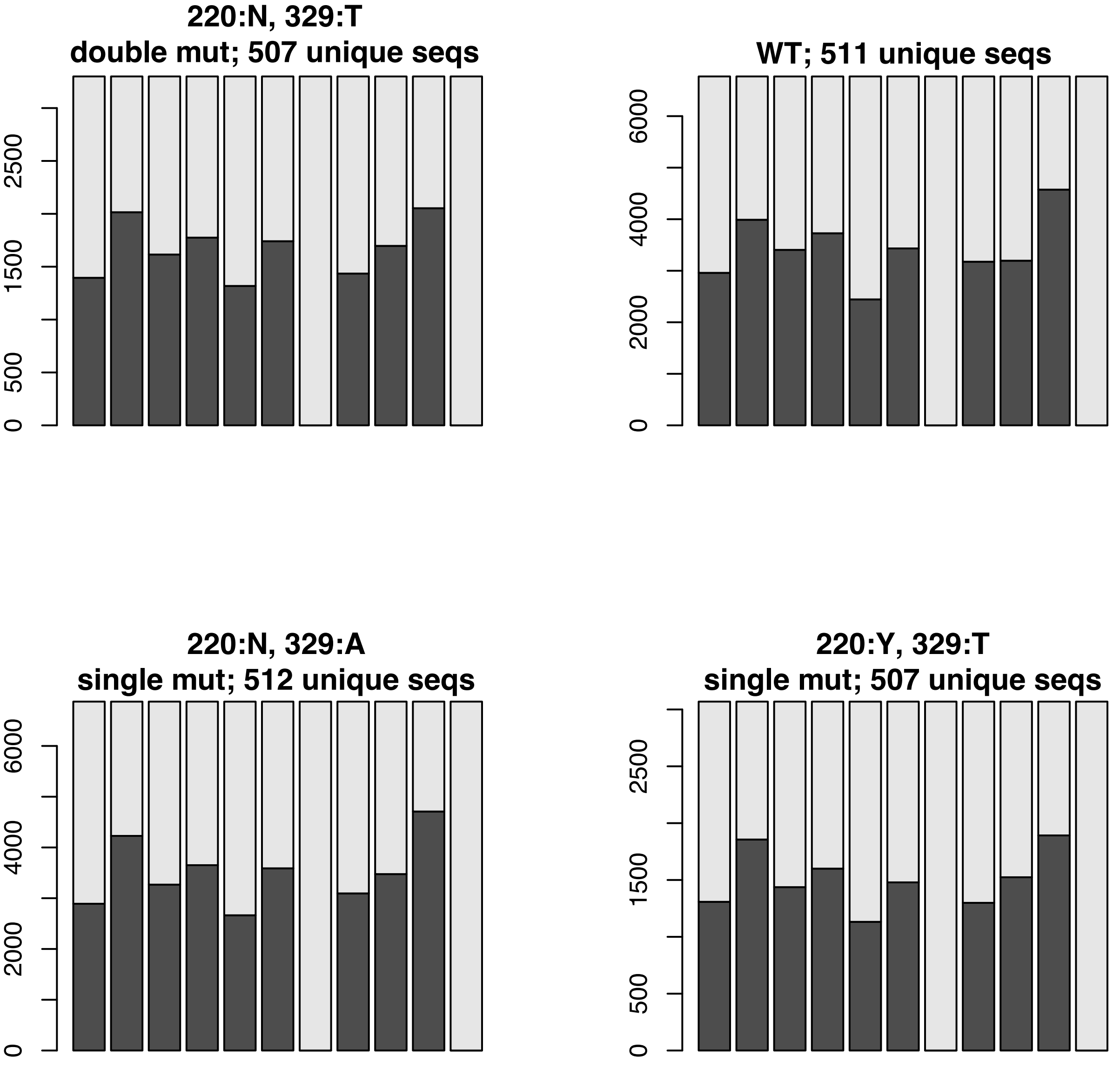
220:N, 329:T
double mut; 507 unique seqs
0
500
1500
2500
WT; 511 unique seqs
0
2000
4000
6000
220:N, 329:A
single mut; 512 unique seqs
0
2000
4000
6000
220:Y, 329:T
single mut; 507 unique seqs
0
500
1500
2500

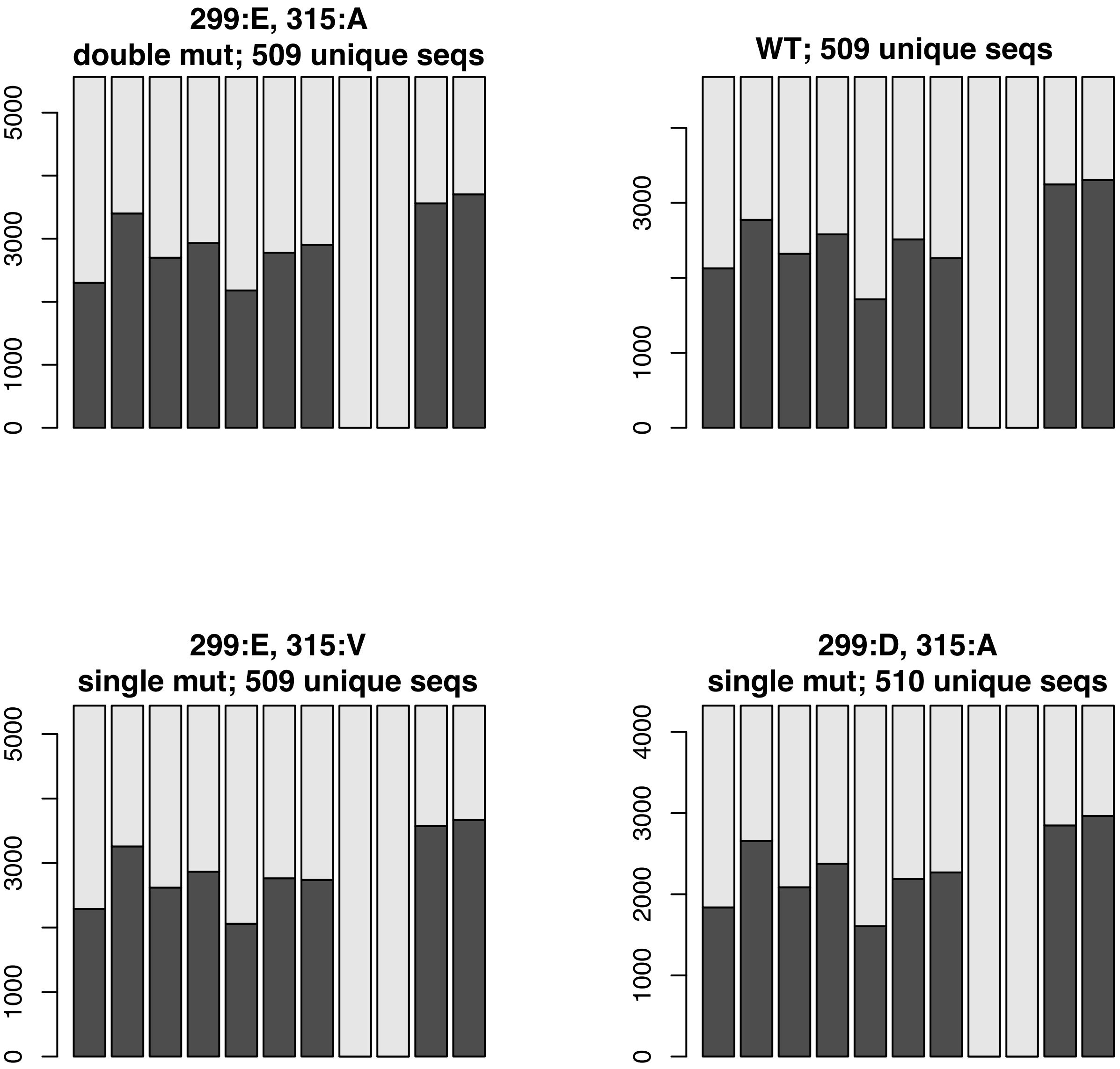

299:E, 315:A
double mut; 509 unique seqs
0
1000
3000
5000
WT; 509 unique seqs
0
1000
3000
299:E, 315:V
single mut; 509 unique seqs
0
1000
3000
5000
299:D, 315:A
single mut; 510 unique seqs
0
1000
2000
3000
4000

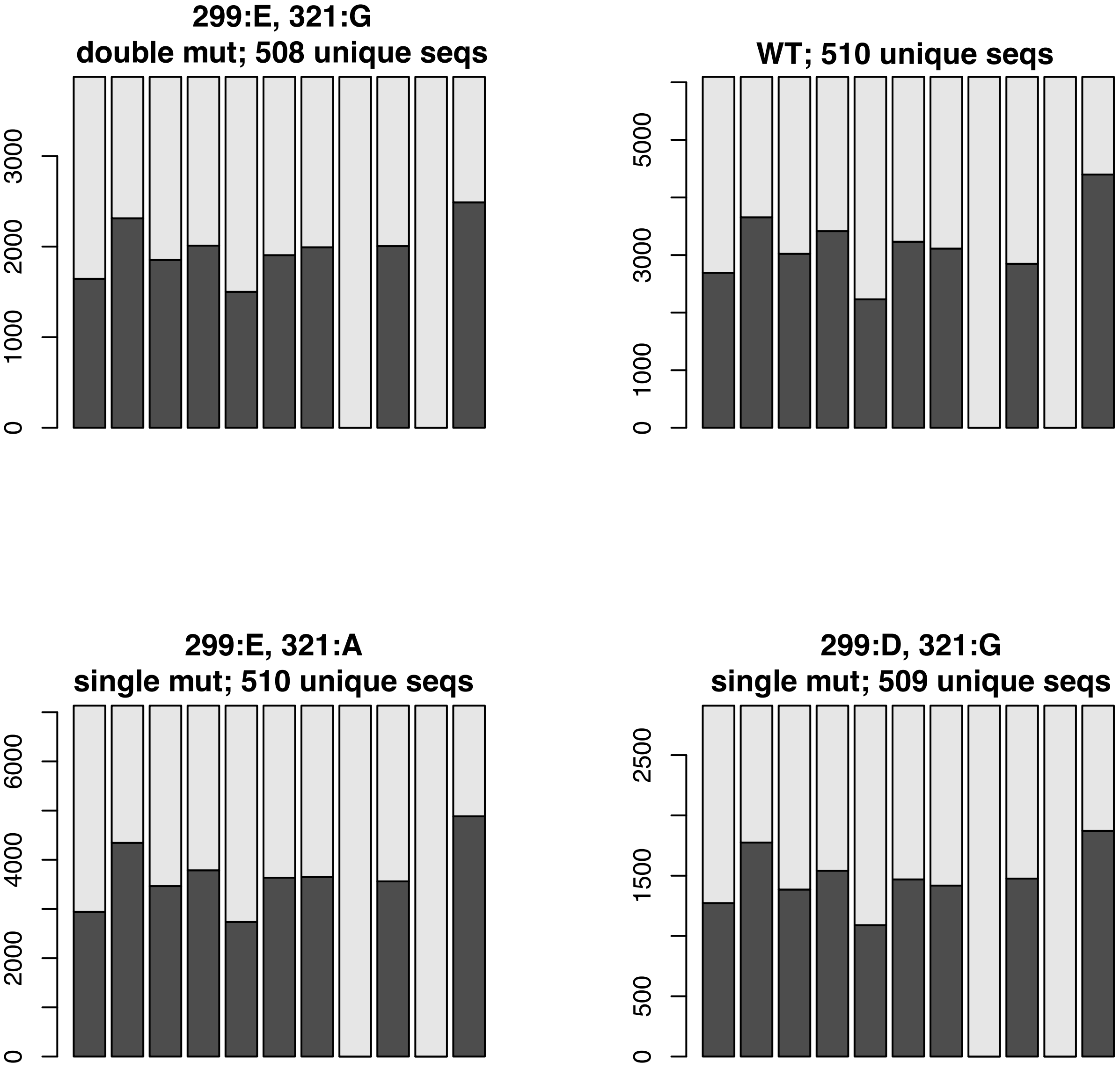
299:E, 321:G
double mut; 508 unique seqs
0
1000
2000
3000
WT; 510 unique seqs
0
1000
3000
5000
299:E, 321:A
single mut; 510 unique seqs
0
2000
4000
6000
299:D, 321:G
single mut; 509 unique seqs
0
500
1500
2500

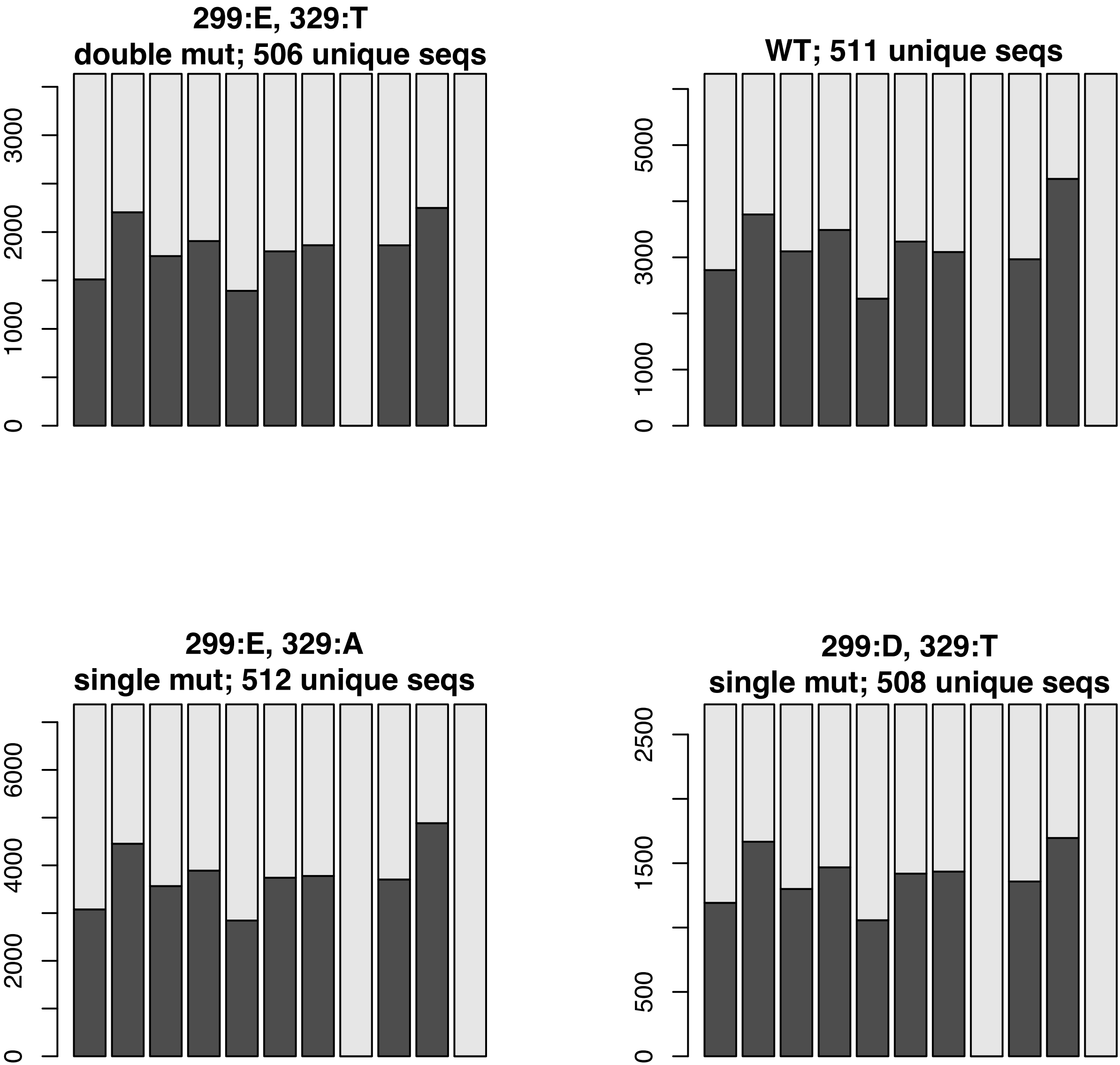
299:E, 329:T
double mut; 506 unique seqs
0
1000
2000
3000
WT; 511 unique seqs
0
1000
3000
5000
299:E, 329:A
single mut; 512 unique seqs
0
2000
4000
6000
299:D, 329:T
single mut; 508 unique seqs
0
500
1500
2500

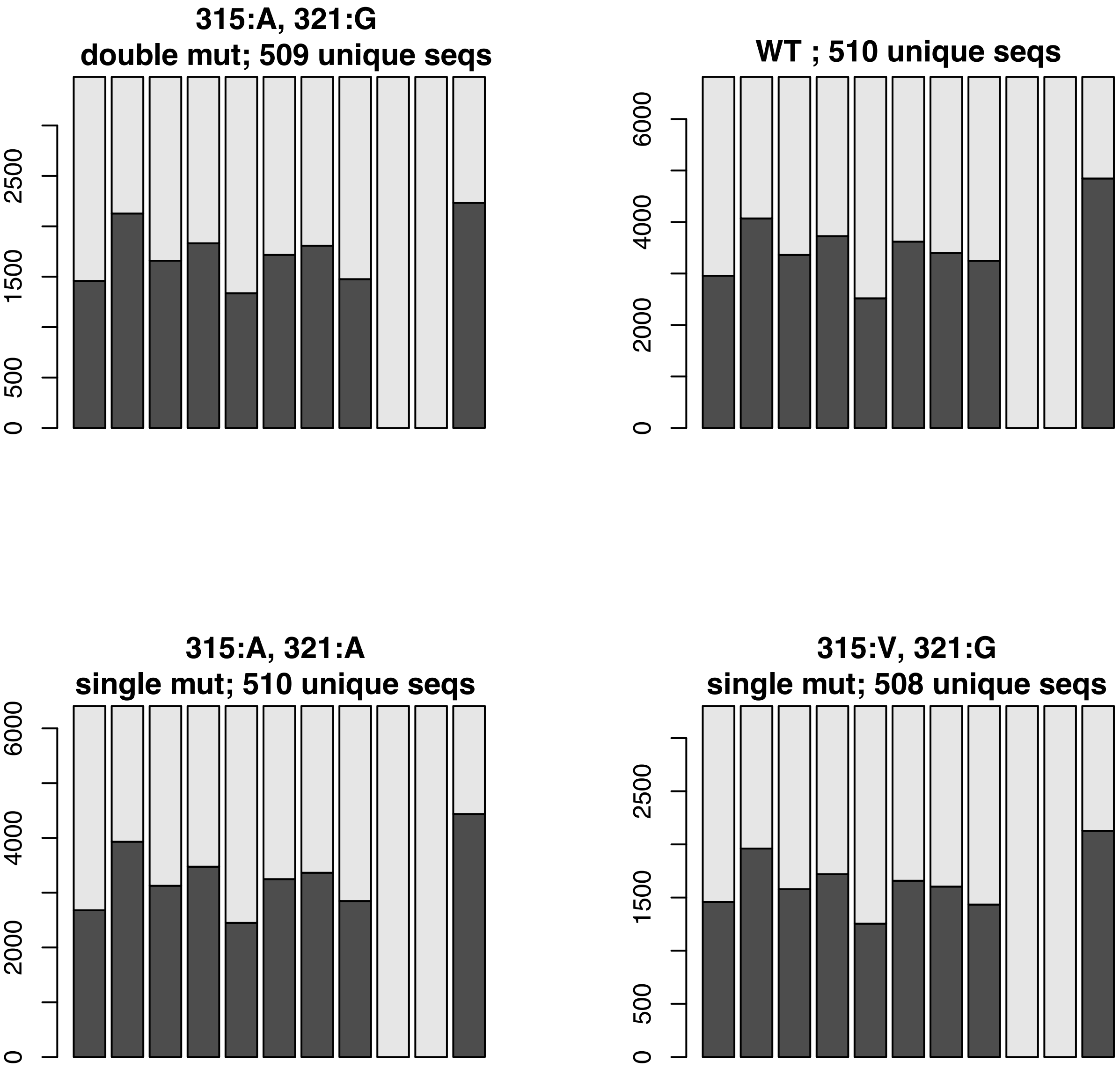
315:A, 321:G
double mut; 509 unique seqs
0
500
1500
2500
WT ; 510 unique seqs
0
2000
4000
6000
315:A, 321:A
single mut; 510 unique seqs
0
2000
4000
6000
315:V, 321:G
single mut; 508 unique seqs
0
500
1500
2500

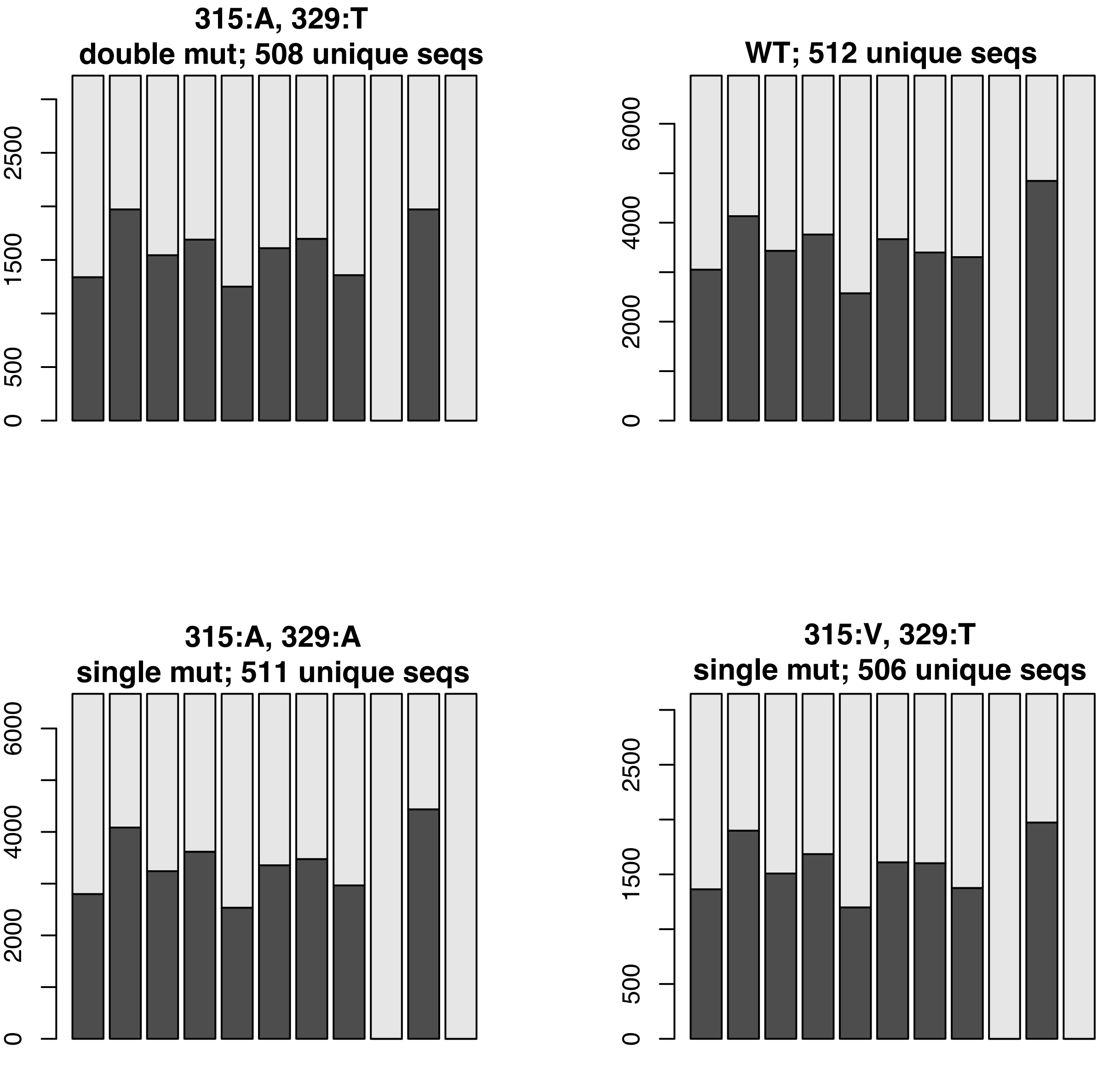

315:A, 329:T
double mut; 508 unique seqs
0
500
1500
2500
WT; 512 unique seqs
0
2000
4000
6000
315:A, 329:A
single mut; 511 unique seqs
0
2000
4000
6000
315:V, 329:T
single mut; 506 unique seqs
0
500
1500
2500

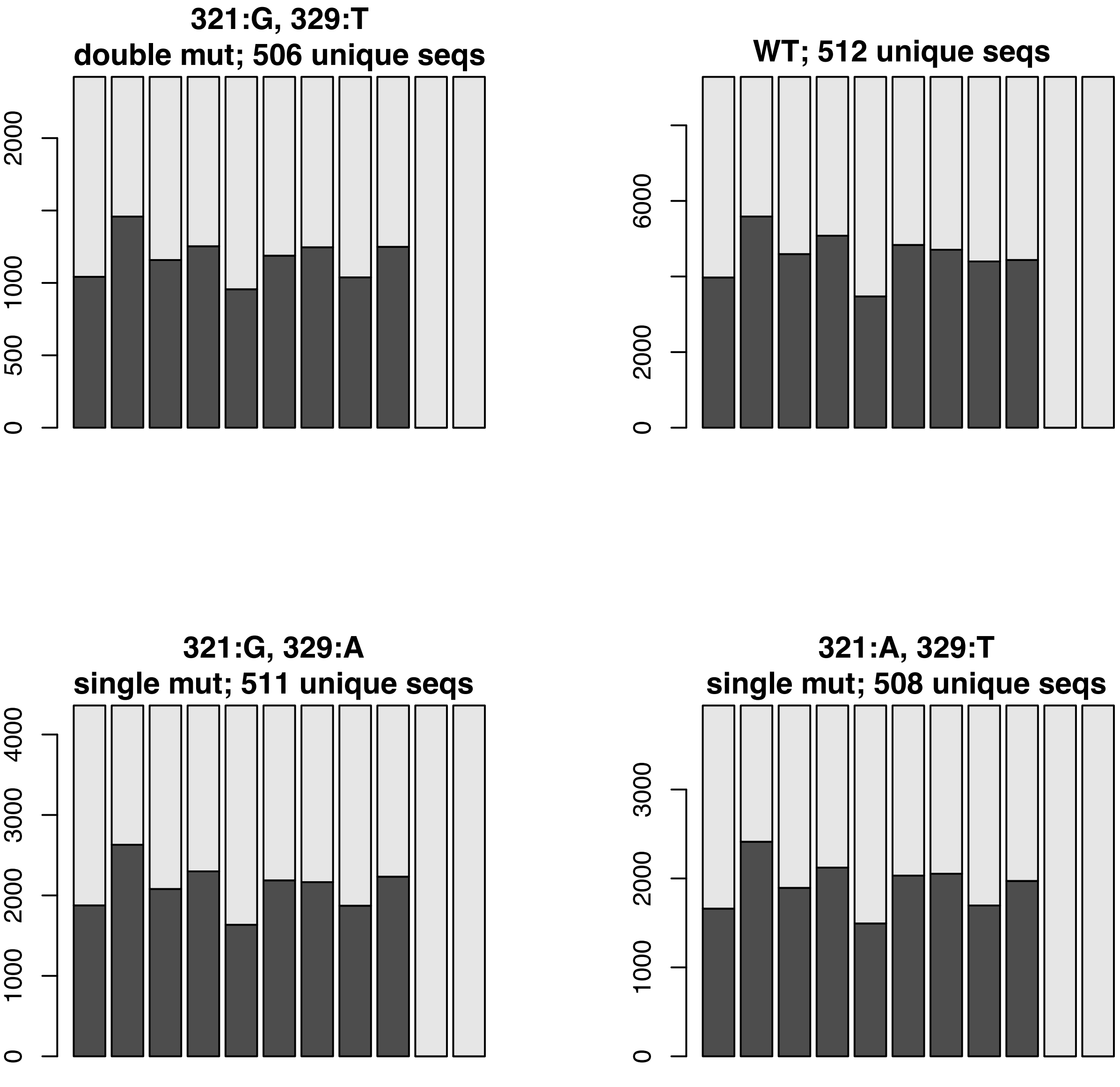

321:G, 329:T
double mut; 506 unique seqs
0
500
1000
2000
WT; 512 unique seqs
0
2000
6000
321:G, 329:A
single mut; 511 unique seqs
0
1000
2000
3000
4000
321:A, 329:T
single mut; 508 unique seqs
0
1000
2000
3000
