# Supplementary material for: Primary and promiscuous functions coexist during evolutionary innovation through whole protein domain acquisitions
Source: eLife. 2020 Dec 15;9:e58061. doi: 10.7554/eLife.58061 (PMC7790495; doi:10.7554/eLife.58061)
Supplement: Supplementary file 5. [file elife-58061-supp5.pdf]

# Fitness Distribution Observed

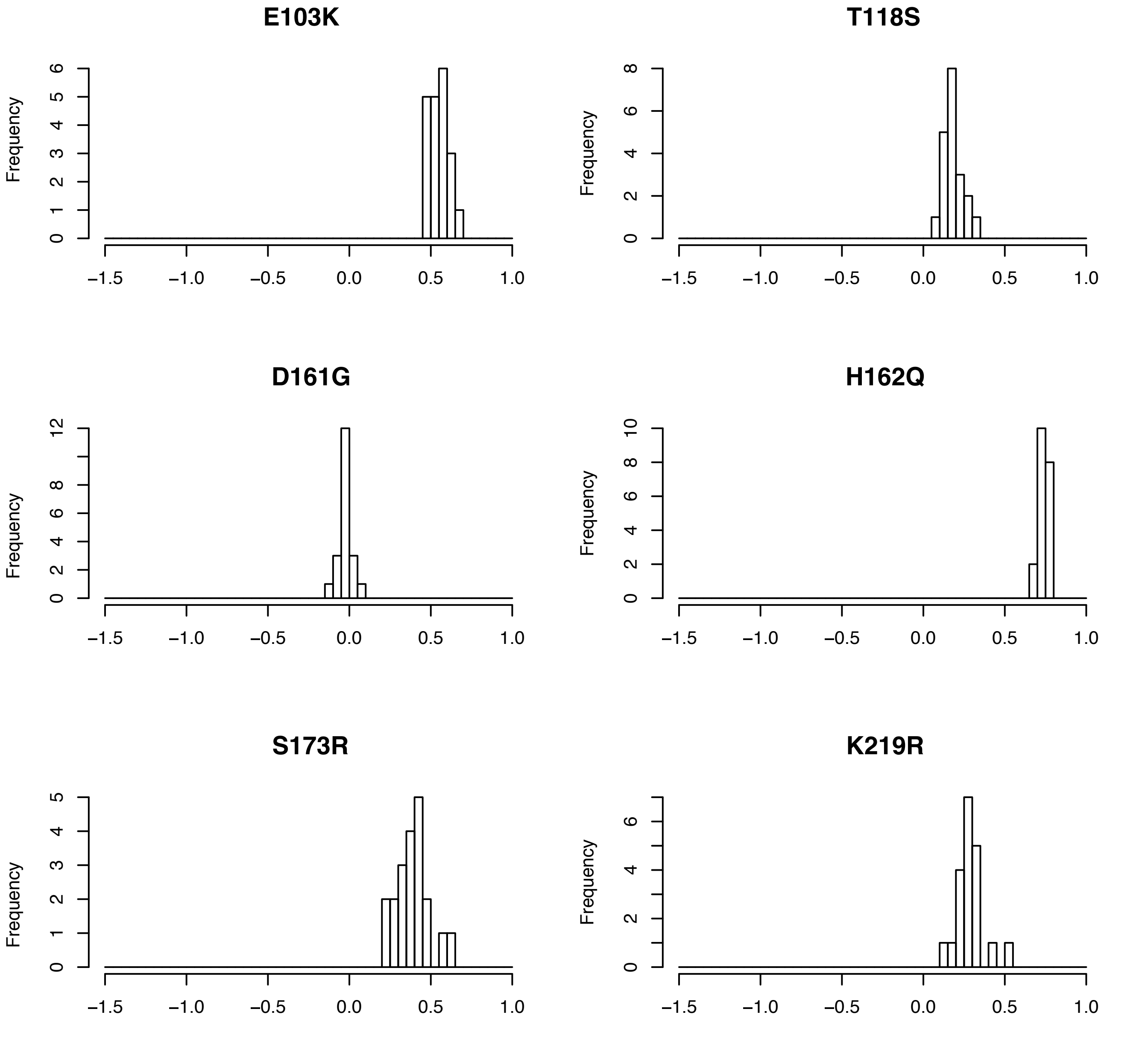

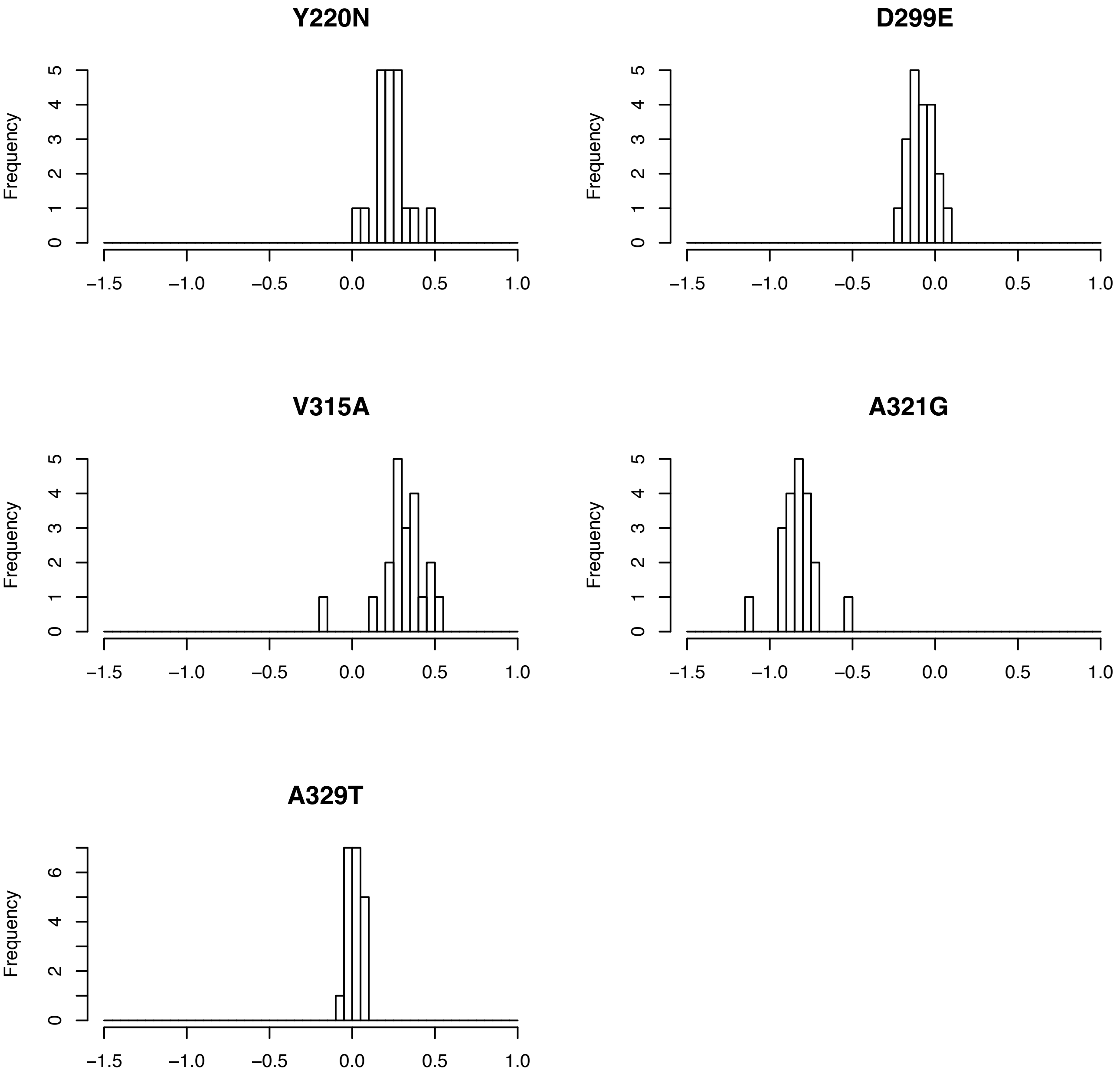
Y220N
D299E
V315A
A321G
A329T
Frequency
0
1
2
3
4
5
6
−1.5
−1.0
−0.5
0.0
0.5
1.0

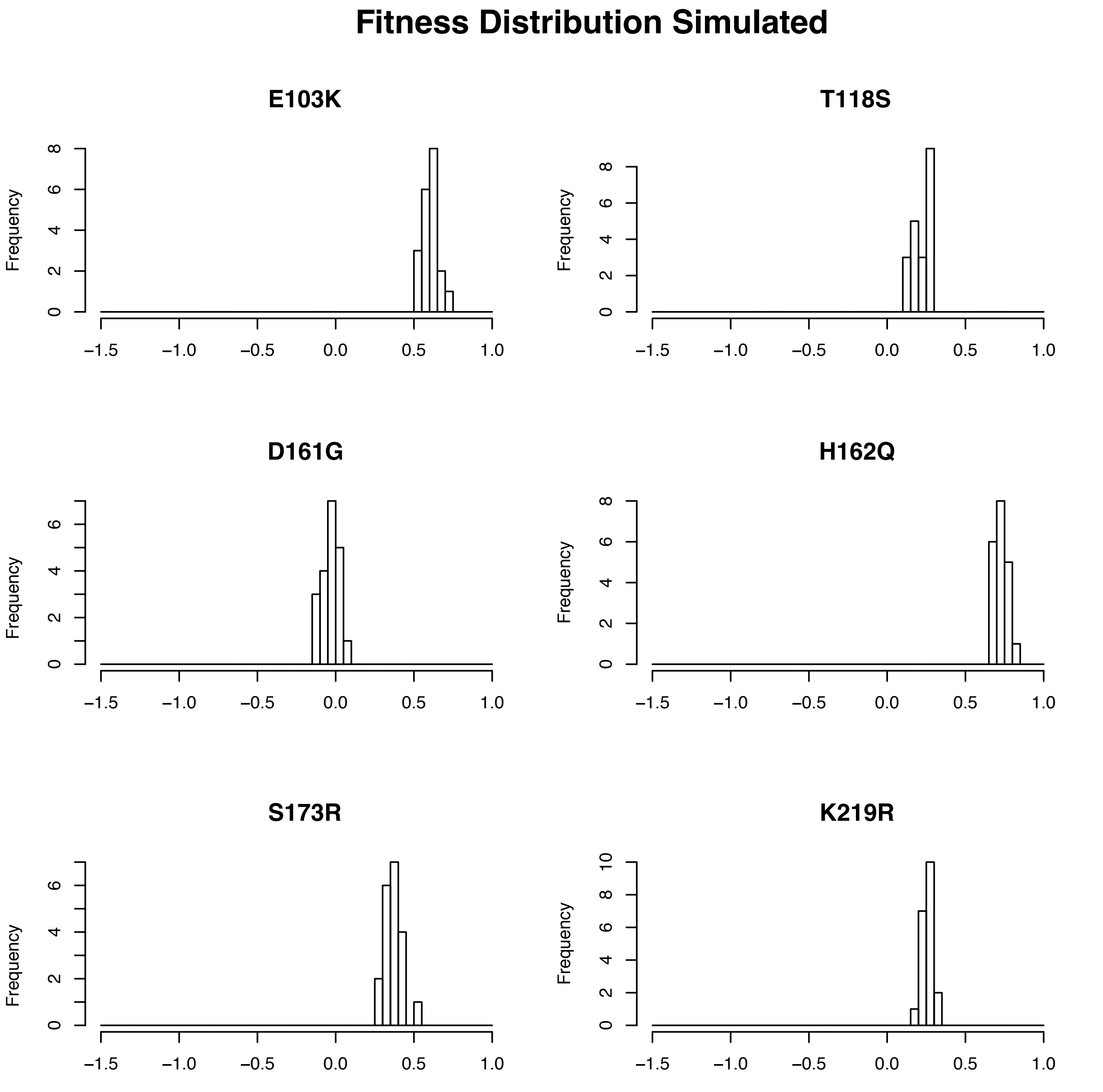

Fitness Distribution Simulated
E103K
T118S
D161G
H162Q
S173R
K219R
Frequency
−1.5
−1.0
−0.5
0.0
0.5
1.0
0
2
4
6
8
10

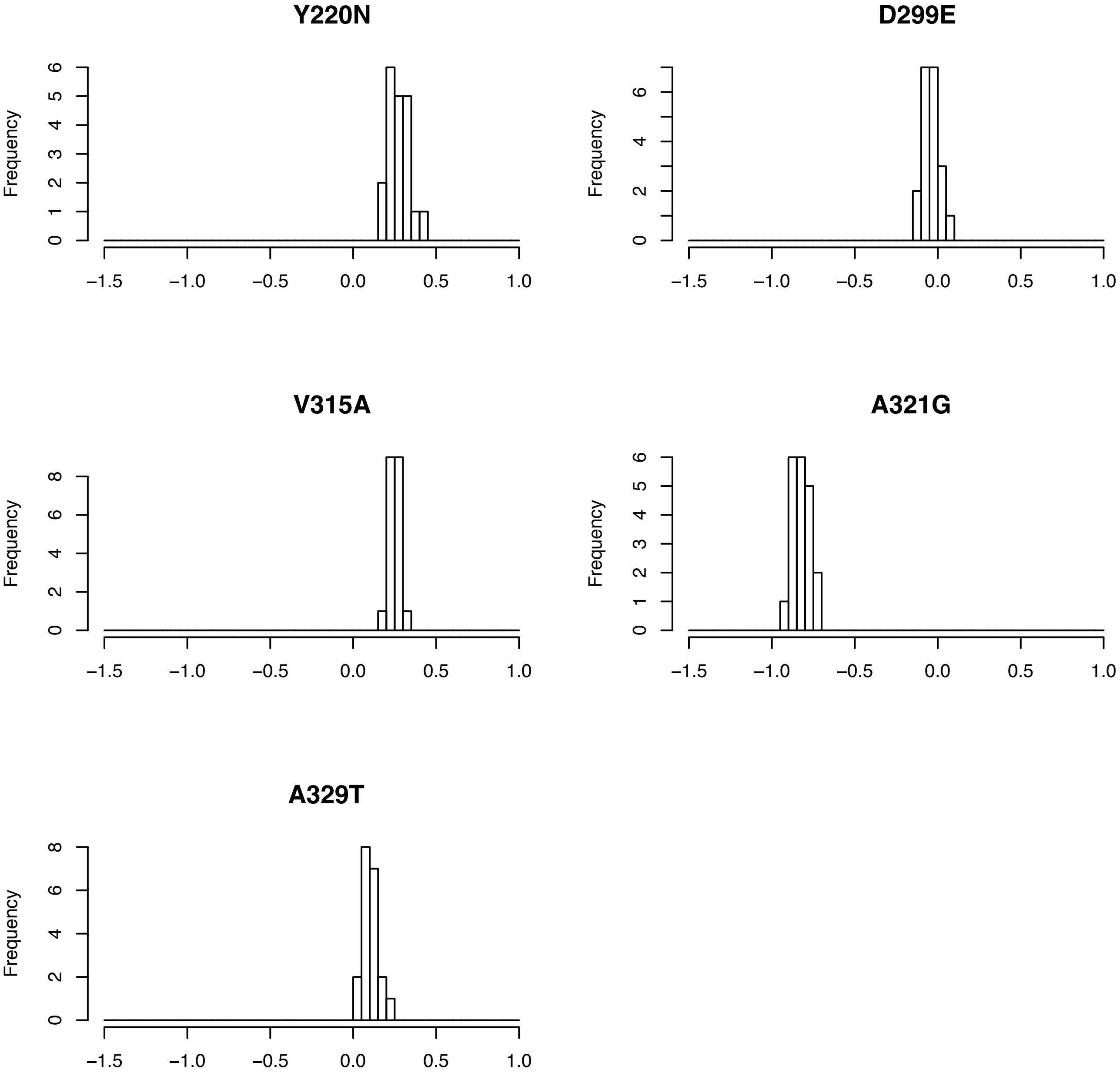
Y220N
D299E
V315A
A321G
A329T
Frequency
−1.5
−1.0
−0.5
0.0
0.5
1.0
