# Supplementary material for: Primary and promiscuous functions coexist during evolutionary innovation through whole protein domain acquisitions
Source: eLife. 2020 Dec 15;9:e58061. doi: 10.7554/eLife.58061 (PMC7790495; doi:10.7554/eLife.58061)
Supplement: Supplementary file 6. — Red lines show epistasis measured from the real data. [file elife-58061-supp6.pdf]

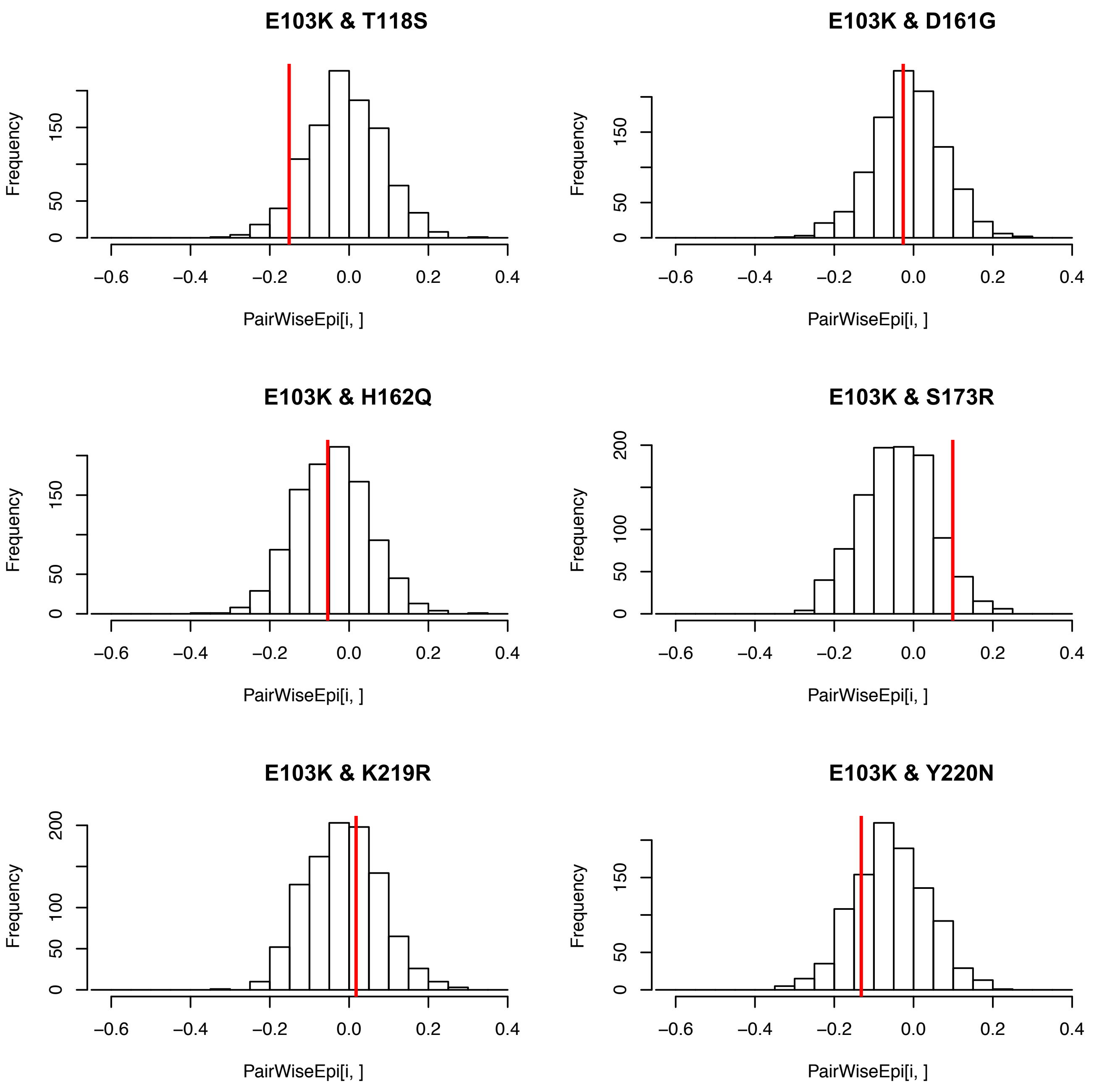
E103K & T118S
Frequency
0
50
150
−0.6
−0.4
−0.2
0.0
0.2
0.4
PairWiseEpi[i,]
E103K & D161G
Frequency
0
50
150
−0.6
−0.4
−0.2
0.0
0.2
0.4
PairWiseEpi[i,]
E103K & H162Q
Frequency
0
50
150
−0.6
−0.4
−0.2
0.0
0.2
0.4
PairWiseEpi[i,]
E103K & S173R
Frequency
0
50
100
200
−0.6
−0.4
−0.2
0.0
0.2
0.4
PairWiseEpi[i,]
E103K & K219R
Frequency
0
50
100
200
−0.6
−0.4
−0.2
0.0
0.2
0.4
PairWiseEpi[i,]
E103K & Y220N
Frequency
0
50
150
−0.6
−0.4
−0.2
0.0
0.2
0.4
PairWiseEpi[i,]

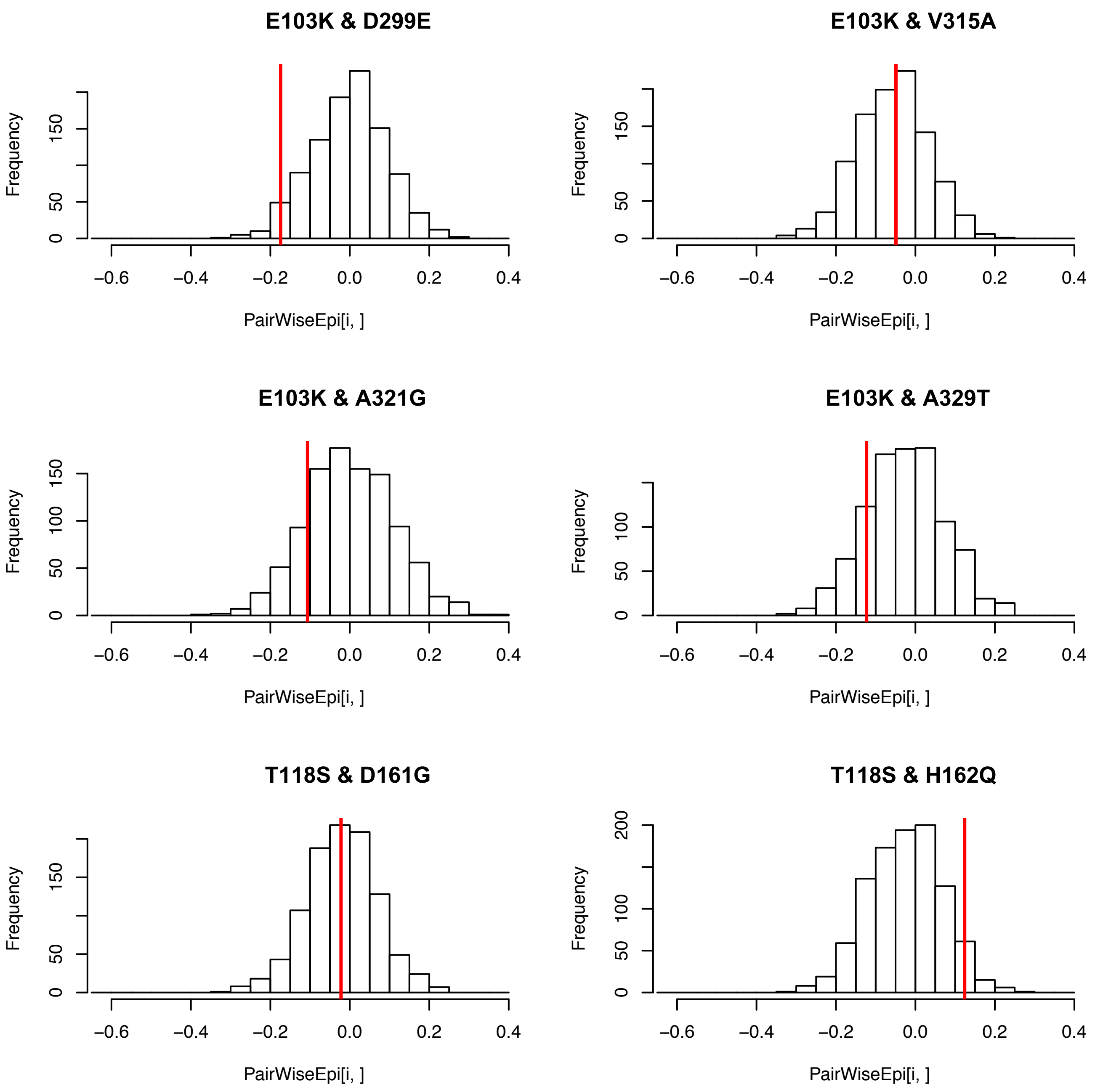
E103K & D299E
E103K & V315A
E103K & A321G
E103K & A329T
T118S & D161G
T118S & H162Q
Frequency
0
50
100
150
200
−0.6
−0.4
−0.2
0.0
0.2
0.4
PairWiseEpi[i,]

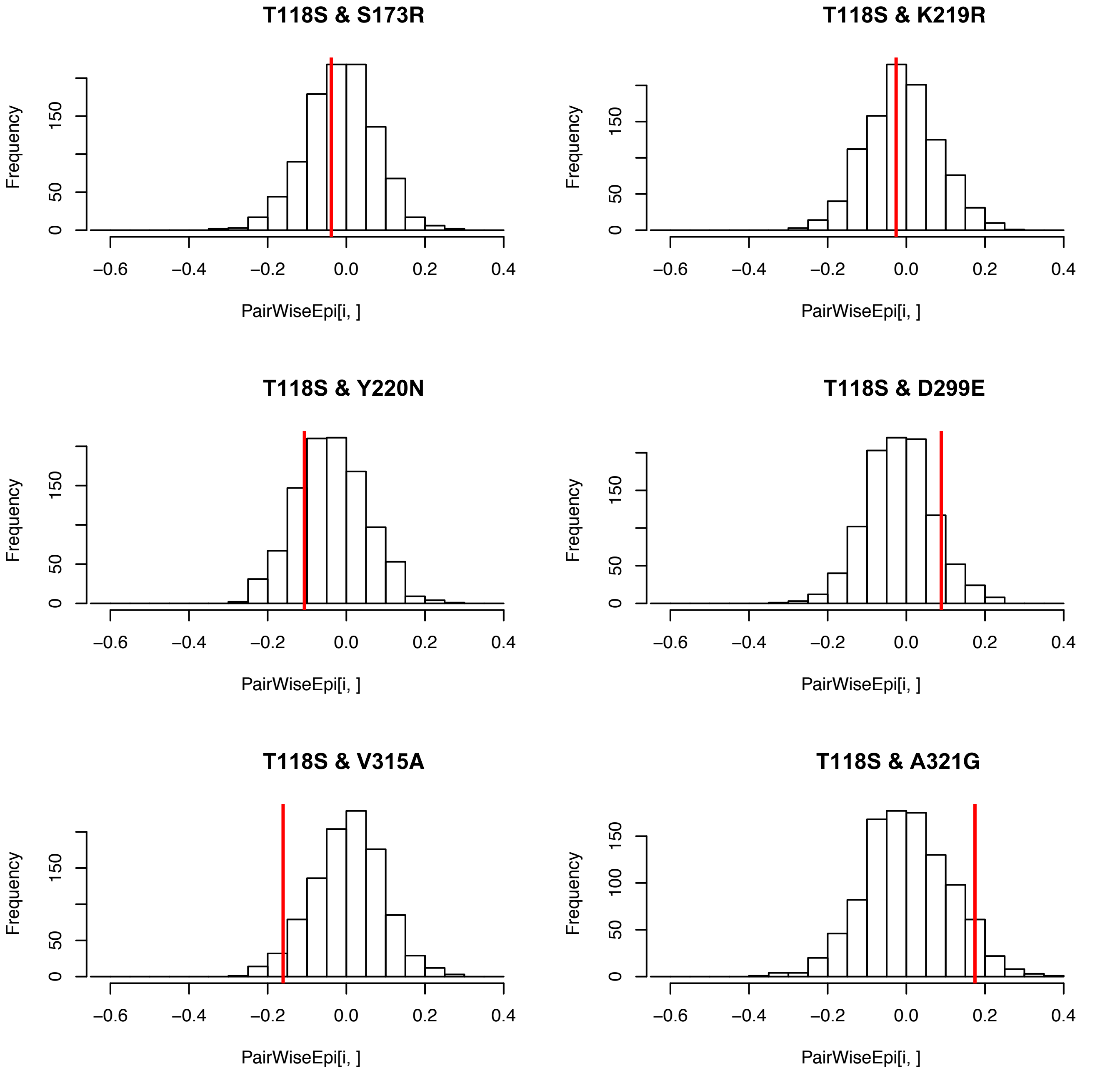
T118S & S173R
T118S & K219R
T118S & Y220N
T118S & D299E
T118S & V315A
T118S & A321G
Frequency
0
50
100
150
−0.6
−0.4
−0.2
0.0
0.2
0.4
PairWiseEpi[i,]

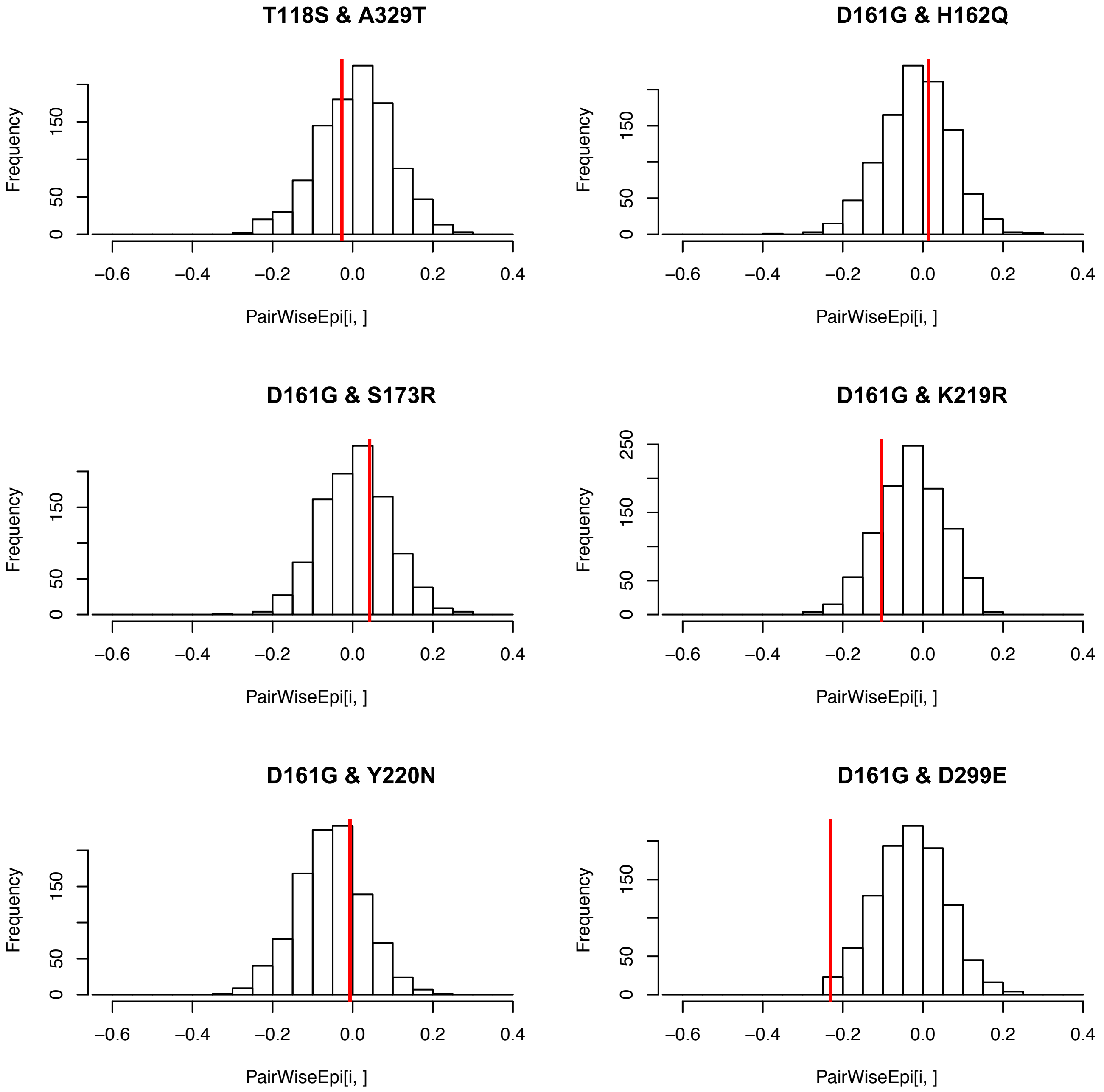
T118S & A329T
D161G & H162Q
D161G & S173R
D161G & K219R
D161G & Y220N
D161G & D299E
Frequency
0
50
150
250
−0.6
−0.4
−0.2
0.0
0.2
0.4
PairWiseEpi[i,]

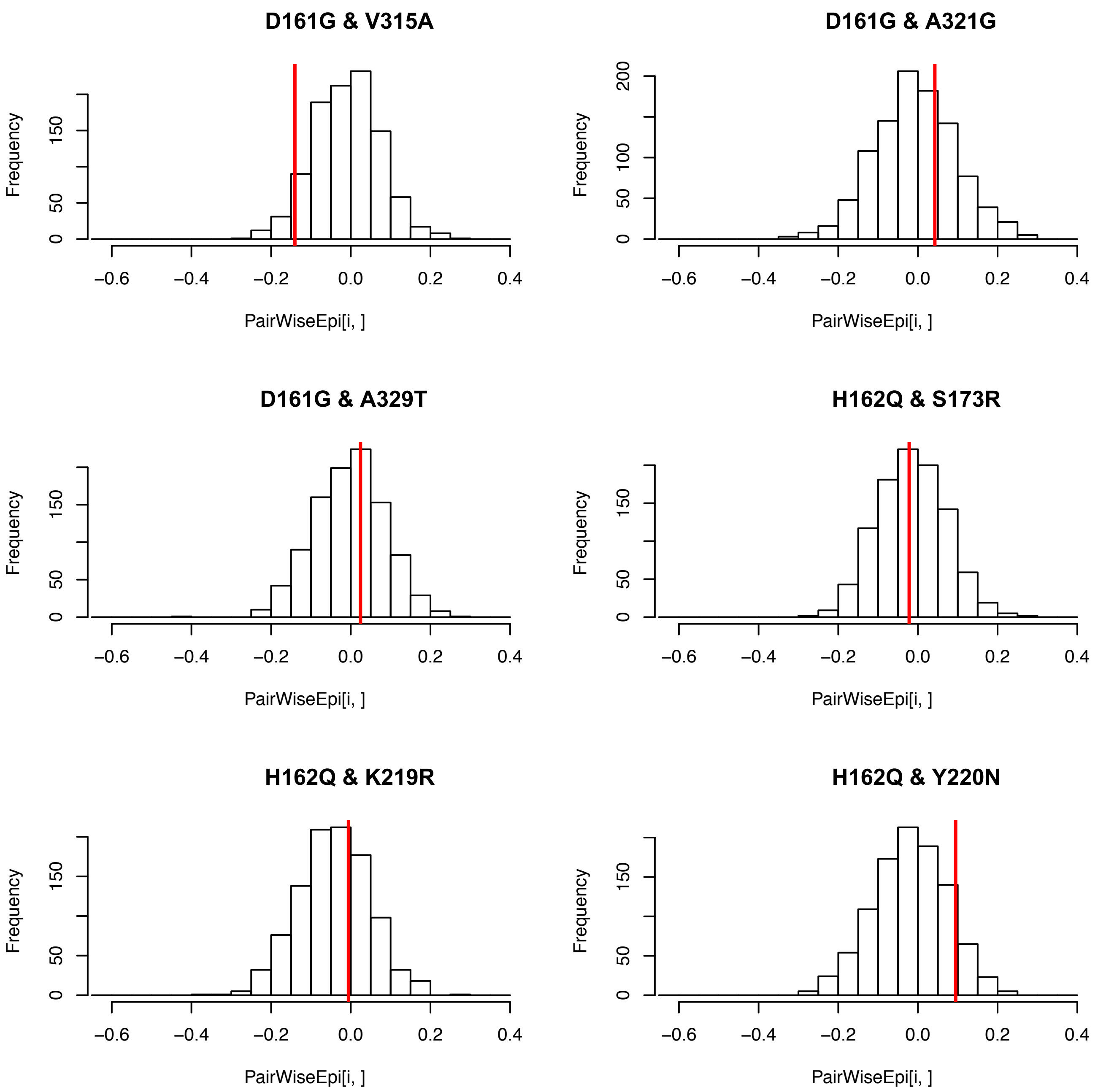
D161G & V315A
D161G & A321G
D161G & A329T
H162Q & S173R
H162Q & K219R
H162Q & Y220N
Frequency
PairWiseEpi[i,]
0
50
100
150
200
−0.6
−0.4
−0.2
0.0
0.2
0.4

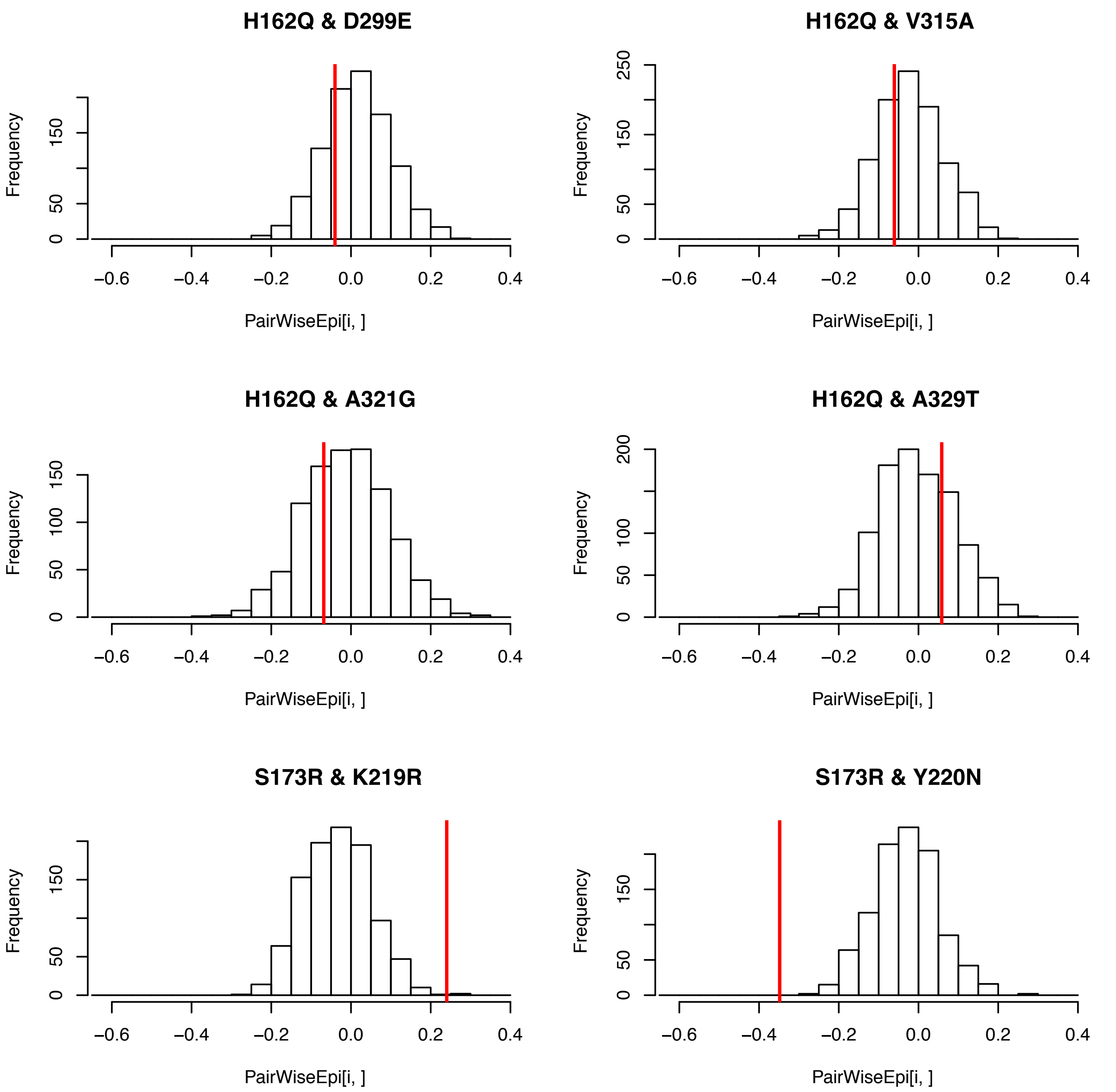

H162Q & D299E
H162Q & V315A
H162Q & A321G
H162Q & A329T
S173R & K219R
S173R & Y220N
Frequency
PairWiseEpi[i,]

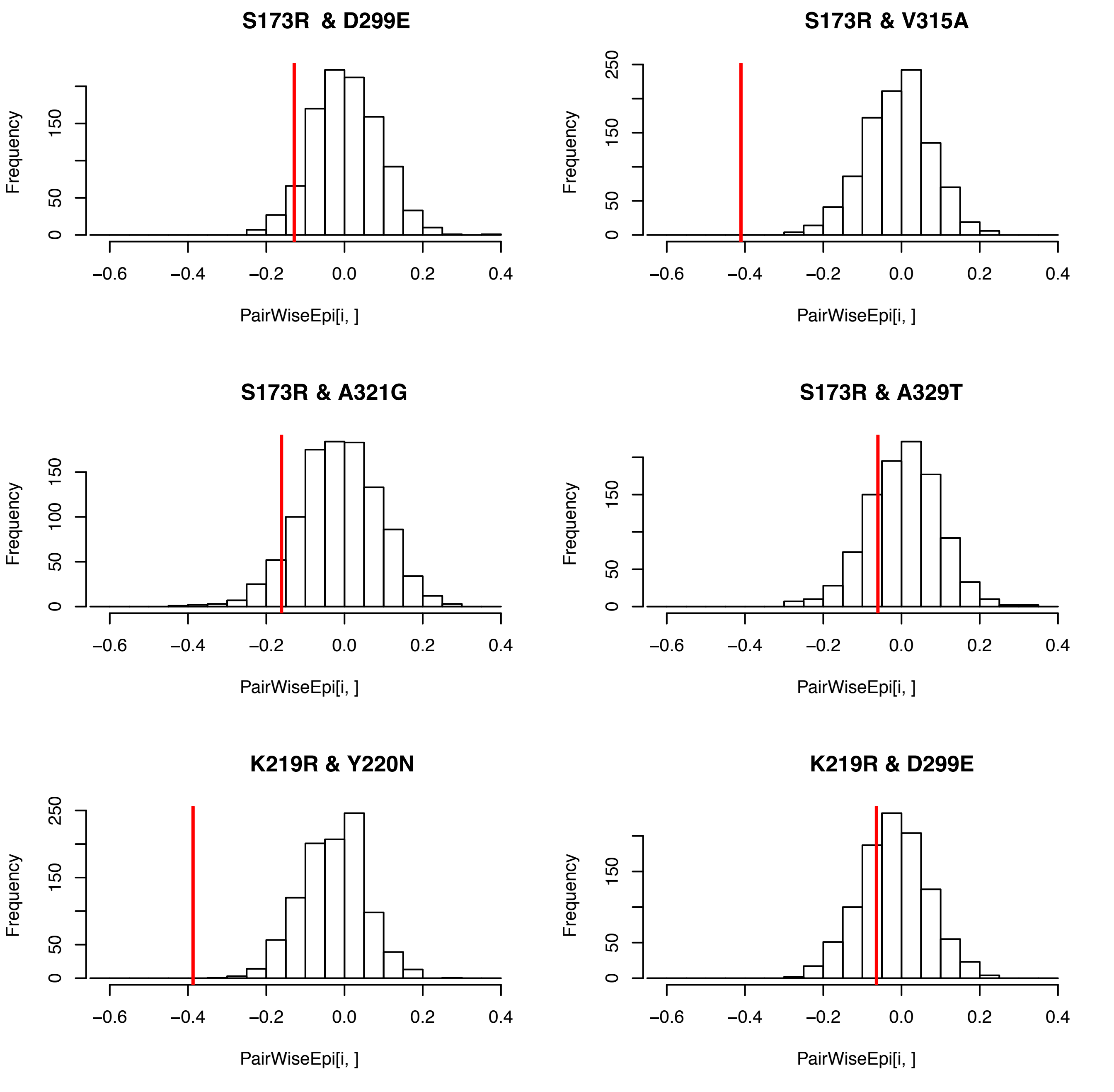
S173R & D299E
S173R & V315A
S173R & A321G
S173R & A329T
K219R & Y220N
K219R & D299E
Frequency
PairWiseEpi[i,]
0
50
150
250
−0.6
−0.4
−0.2
0.0
0.2
0.4

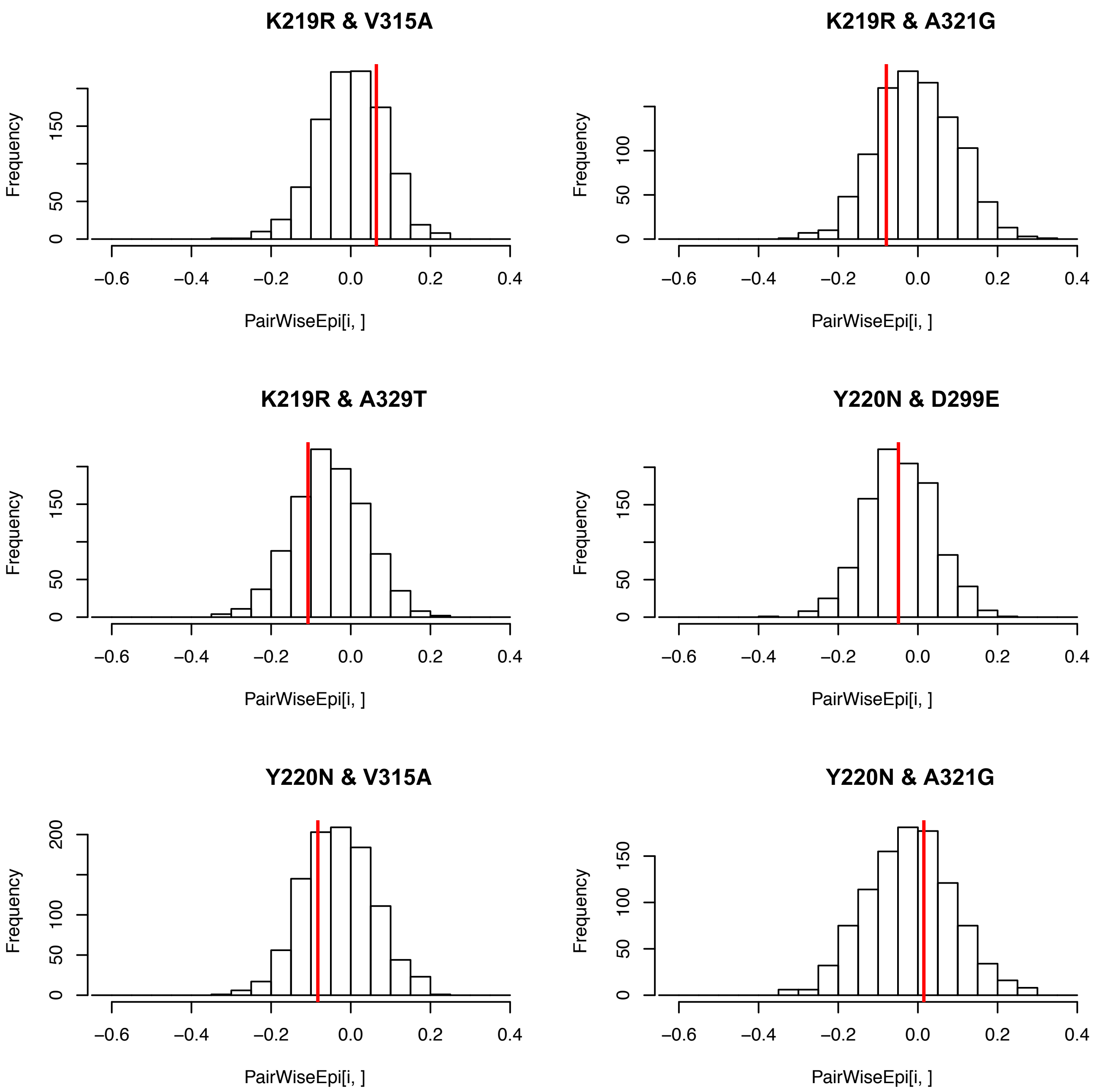
K219R & V315A
K219R & A321G
K219R & A329T
Y220N & D299E
Y220N & V315A
Y220N & A321G
Frequency
PairWiseEpi[i,]
−0.6
−0.4
−0.2
0.0
0.2
0.4
0
50
100
150
200

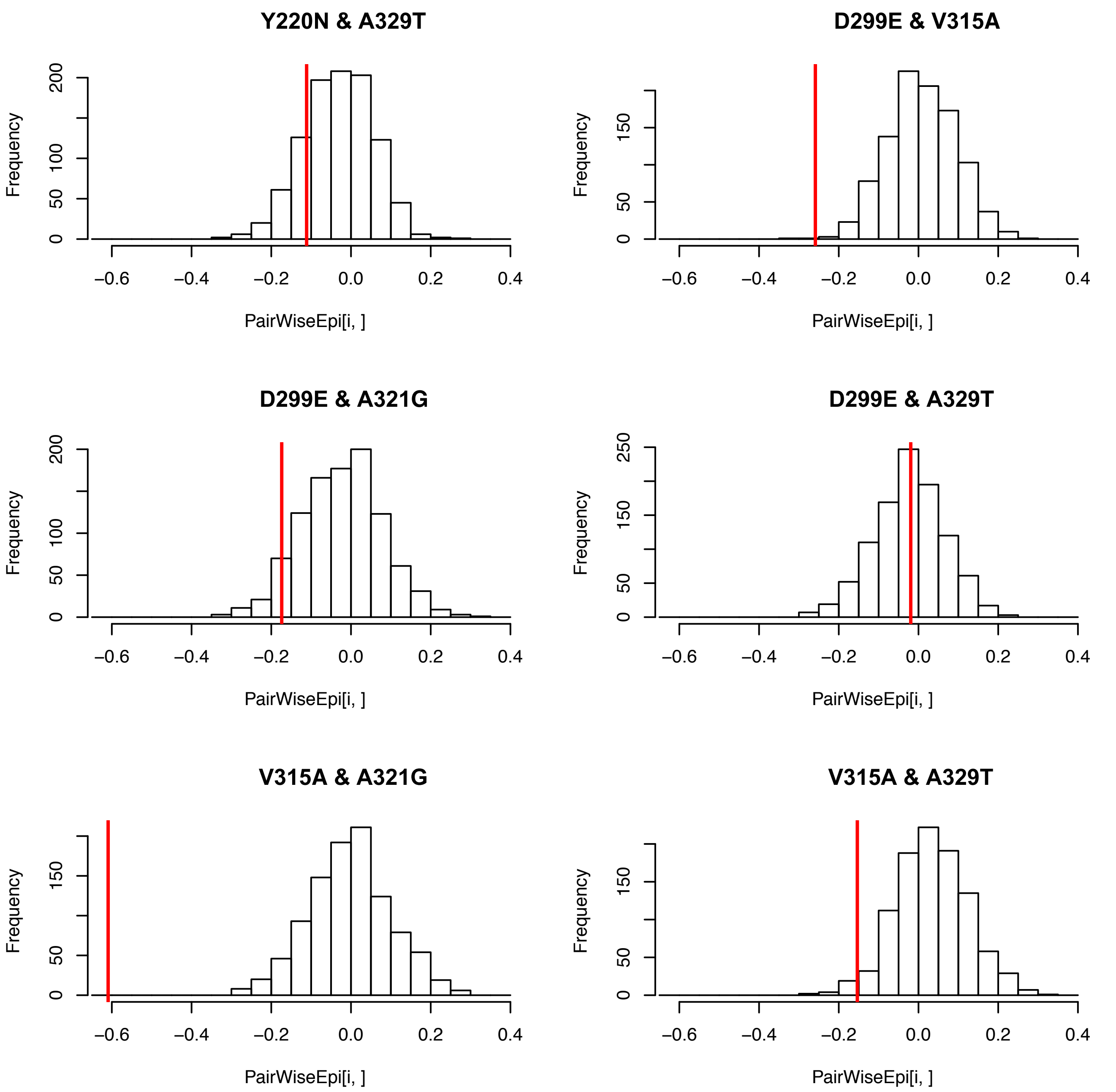

Y220N & A329T
Frequency
0
50
100
200
−0.6
−0.4
−0.2
0.0
0.2
0.4
PairWiseEpi[i,]
D299E & V315A
Frequency
0
50
150
−0.6
−0.4
−0.2
0.0
0.2
0.4
PairWiseEpi[i,]
D299E & A321G
Frequency
0
50
100
200
−0.6
−0.4
−0.2
0.0
0.2
0.4
PairWiseEpi[i,]
D299E & A329T
Frequency
0
50
150
250
−0.6
−0.4
−0.2
0.0
0.2
0.4
PairWiseEpi[i,]
V315A & A321G
Frequency
0
50
150
−0.6
−0.4
−0.2
0.0
0.2
0.4
PairWiseEpi[i,]
V315A & A329T
Frequency
0
50
150
−0.6
−0.4
−0.2
0.0
0.2
0.4
PairWiseEpi[i,]

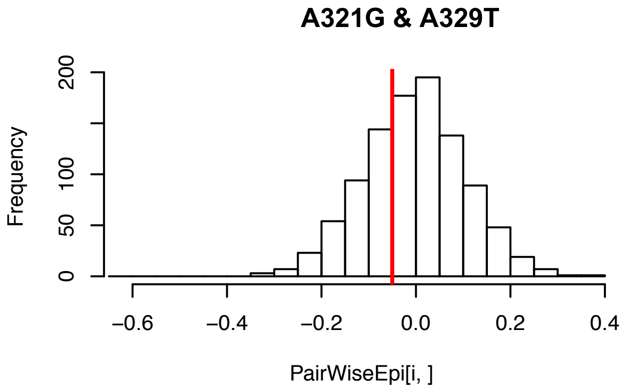

A321G & A329T
Frequency
0
50
100
200
−0.6
−0.4
−0.2
0.0
0.2
0.4
PairWiseEpi[i,]
